# Global prediction of chromatin accessibility using small-cell-number and single-cell RNA-seq

**DOI:** 10.1093/nar/gkz716

**Published:** 2019-08-20

**Authors:** Weiqiang Zhou, Zhicheng Ji, Weixiang Fang, Hongkai Ji

**Affiliations:** Department of Biostatistics, Johns Hopkins University Bloomberg School of Public Health, 615 North Wolfe Street, Baltimore, MD 21205, USA

## Abstract

Conventional high-throughput genomic technologies for mapping regulatory element activities in bulk samples such as ChIP-seq, DNase-seq and FAIRE-seq cannot analyze samples with small numbers of cells. The recently developed low-input and single-cell regulome mapping technologies such as ATAC-seq and single-cell ATAC-seq (scATAC-seq) allow analyses of small-cell-number and single-cell samples, but their signals remain highly discrete or noisy. Compared to these regulome mapping technologies, transcriptome profiling by RNA-seq is more widely used. Transcriptome data in single-cell and small-cell-number samples are more continuous and often less noisy. Here, we show that one can globally predict chromatin accessibility and infer regulatory element activities using RNA-seq. Genome-wide chromatin accessibility predicted by RNA-seq from 30 cells can offer better accuracy than ATAC-seq from 500 cells. Predictions based on single-cell RNA-seq (scRNA-seq) can more accurately reconstruct bulk chromatin accessibility than using scATAC-seq. Integrating ATAC-seq with predictions from RNA-seq increases the power and value of both methods. Thus, transcriptome-based prediction provides a new tool for decoding gene regulatory circuitry in samples with limited cell numbers.

## INTRODUCTION

Decoding gene regulatory network in developmental systems and precious clinical samples often requires measuring transcriptome (i.e. genes’ transcriptional activities) and regulome (i.e. regulatory element activities) in samples with small numbers of cells or in single cells. While significant progress has been made to measure transcriptome in single-cell (1,2) and in small-cell-number (3) samples using RNA sequencing (RNA-seq), accurately measuring regulome in single-cell and small-cell-number samples remains a challenge.

Conventional high-throughput regulome mapping technologies such as chromatin immunoprecipitation followed by sequencing (ChIP-seq) (4), sequencing of DNase I hypersensitive sites (DNase-seq) (5), and Formaldehyde-Assisted Isolation of Regulatory Elements coupled with sequencing (FAIRE-seq) (6) require large amounts of input material (∼10^6^ cells). These ‘bulk’ technologies cannot analyze samples with small numbers of cells. The state-of-the-art low-input technology, assay for transposase-accessible chromatin using sequencing (ATAC-seq), can analyze chromatin accessibility in bulk samples with 500–50 000 cells (7). However, ATAC-seq data are noisy when the cell number is <500. Similarly, other recent low-input methods, such as microfluidic oscillatory washing-based ChIP-seq (MOWChIP-seq) for measuring histone modifications (8), also remain noisy when the cell number is below a few hundreds.

**Figure 1.**
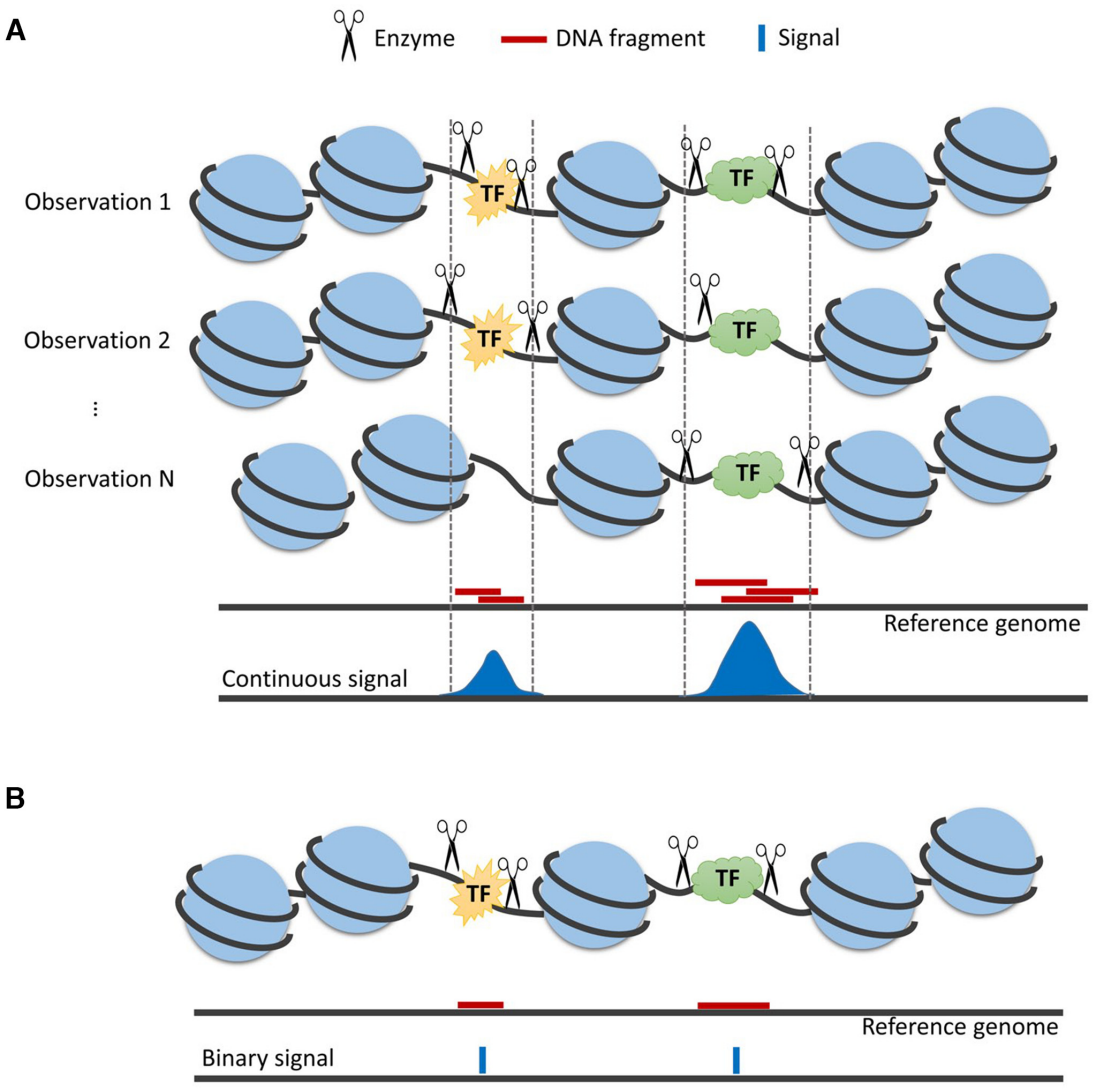
Limitations of existing single-cell regulome mapping technologies. (**A**) Molecular events such as TF-DNA binding and dissociation are stochastic. For instance, a TF may bind to DNA at one time point and then dissociate from DNA at the next time point. If one observes a cell at a random time point, there is certain probability to observe TF binding, and there is also certain probability to observe no binding. Similarly, if hypothetically one could apply Tn5 treatment in ATAC-seq to the same cell at multiple random time points, Tn5 insertion may occur at some time points but not others. Chromatin accessibility reflects the probability that such molecular events occur at a genomic locus. Regions with higher accessibility tend to have higher probabilities to host such molecular events. Since probability is a continuous measure, chromatin accessibility is arguably a continuous signal. (**B**) Current single-cell regulome mapping technologies cannot repeatedly measure the same cell multiple times. Instead, they can only measure a cell once since the cell is destroyed during the experiment. Moreover, each genomic locus only has two alleles that can be assayed. As a result, the measurements (i.e. event counts) are highly discrete. Such discrete signals obtained at a single time point are not adequate for describing the continuum of chromatin accessibility (i.e. the probability that molecular events occur at each locus). For instance, observing TF binding at a particular time point is not sufficient for describing the average steady-state TF binding behavior of a cell over time since TF binding may not occur at other time points.

Recently, single-cell ATAC-seq (9,10) (scATAC-seq) has been invented to analyze individual cells. Nevertheless, signals from scATAC-seq are sparse. In a typical dataset, each cell has 10^3^–10^5^ sequence reads. In contrast, the human genome contains 10^6^–10^7^*cis*-regulatory elements (CREs). Thus, in a typical cell, most CREs receive no read. Data from scATAC-seq are intrinsically discrete since each genomic locus only has up to two copies of chromatin that can be assayed within a cell (9–11). This is different from single-cell RNA-seq (scRNA-seq) data which are more continuous because a gene can have multiple assayable transcripts in a cell. Also, each scATAC-seq sample only provides a snapshot of chromatin accessibility of a cell at the time when it is assayed and destroyed. However, chromatin accessibility as a surrogate for regulatory element activity is arguably a continuous signal. This is because molecular events such as transcription factor-DNA binding and dissociation are stochastic over time, and the overall activity of a regulatory element in a single cell is determined by the probability, which is a continuous measure, that such stochastic events occur if one were to repeatedly observe the same cell at random time points. The discrete and sparse signal measured by scATAC-seq at a single time point cannot accurately describe this continuum of chromatin accessibility at each regulatory element (Figure 1).

Besides scATAC-seq, single-cell ChIP-seq (scChIP-seq) (12), single-cell DNase-seq (scDNase-seq) (13) and single-cell transposome hypersensitive site sequencing (scTHS-seq) (14) have also been developed for analyzing regulomes in individual cells. Similar to scATAC-seq, these methods destroy cells during the assay and therefore cannot monitor cells continuously over time. Data produced by these technologies are also sparse and have narrow dynamic range which is not enough for accurately describing the continuous steady-state regulatory element activities within a single cell. For example, scChIP-seq data in (12) contained 500–10 000 reads per cell, compared to 10^3^–10^5^ reads per cell for scATAC-seq data.

The above single-cell technologies cannot measure transcriptome and regulome simultaneously in the same cell. A few methods have recently been developed to analyze chromatin accessibility along with other genomic signals in the same cell. Examples include single-cell nucleosome occupancy and methylome-sequencing (scNOMe-seq) (15) and single-cell nucleosome, methylation and transcription sequencing (scNMT-seq) (16). However, these multi-omics methods have lower throughout to analyze cells and they do not provide the throughput comparable to scRNA-seq and scATAC-seq to analyze massive numbers of cells. Single-cell combinatorial indexing-based co-assay (sci-CAR) is another method that jointly profiles chromatin accessibility and mRNA (17). This method is capable of analyzing thousands of cells, but its co-assayed scATAC-seq yields a median of ∼1400 reads per cell, which is ∼10 times lower than the typical read count per cell (∼10^4^) of the standard single-assayed scATAC-seq. Similar to scATAC-seq, all these multi-omic technologies cannot monitor cells continuously over time, and their measurements also have high noises.

Currently, among all existing single-cell methods, scATAC-seq is the most popular method for single-cell regulome mapping due to its relatively simple and robust protocol and unparalleled throughput to handle large numbers of cells. scRNA-seq, on the other hand, is the most popular method for single-cell transcriptome mapping. These two methods measure either regulome or transcriptome but not both. However, because of their ability to analyze massive numbers of cells, they are used by the Human Cell Atlas (HCA) (18) project as two major tools for mapping human regulomes and transcriptomes at single-cell resolution.

Recently, we found that chromatin accessibility measured by DNase I hypersensitivity (DH) in a bulk sample can be predicted with good accuracy using the sample’s gene expression profile measured by Affymetrix exon array, and we have developed a big data regression approach, BIRD, to handle this challenging high-dimensional prediction problem (19). We have shown that genome-wide chromatin accessibility predicted from gene expression is practically useful in many applications including predicting transcription factor (TF) binding sites (TFBSs) and differential regulatory element activities between different biological conditions (19). Gene expression is the most widely measured high-throughput functional genomic data type. The number of gene expression samples in public databases is orders of magnitude larger than the number of available ChIP-seq, DNase-seq, FAIRE-seq, and ATAC-seq samples. Predicting regulatory element activities using gene expression, therefore, can provide a cost-effective approach to significantly expanding the number of biological samples and studies with regulome information (19). Such information can then be used to guide hypothesis generation and subsequent functional studies (e.g. to determine which regulatory elements to knock out in a functional study). Prediction is useful not only when the experimental regulome data are unavailable but also when such data are available. For example, the predicted chromatin accessibility may serve as pseudo-replicates to improve the signal-to-noise ratio of the experimental DNase-seq and ChIP-seq data.

The study in (19) was limited to using Affymetrix exon array data as predictors. It remains unknown whether RNA-seq, the state-of-the-art transcriptome profiling technology much more widely used than exon arrays, can be used to predict regulome. Moreover, RNA-seq offers unprecedented power for measuring transcriptome in small-cell-number and single-cell samples. However, no previous study has explored the possibility of using single-cell or low-input gene expression data to predict regulome. If such prediction is feasible, it has important practical implications. It can significantly increase the value of the rapidly growing single-cell and low-input gene expression data. For example, scRNA-seq is the most widely used single-cell functional genomic technology, but most scRNA-seq datasets are generated without accompanying single-cell data for other data modalities. In fact, the Phase I of Human Cell Atlas (18) aims to profile 30–100 million cells, most of which will be analyzed by scRNA-seq but not other single-cell genomic technologies. Prediction will not only allow one to use these RNA-seq data to study regulome, but may also provide a solution to simultaneously mapping transcriptome and regulome in the same cell for a massive number of cells. It can also serve as a bridge to integrate both data types to improve signal accuracy. For this reason, studying RNA-seq based prediction is highly practically relevant.

**Figure 2.**
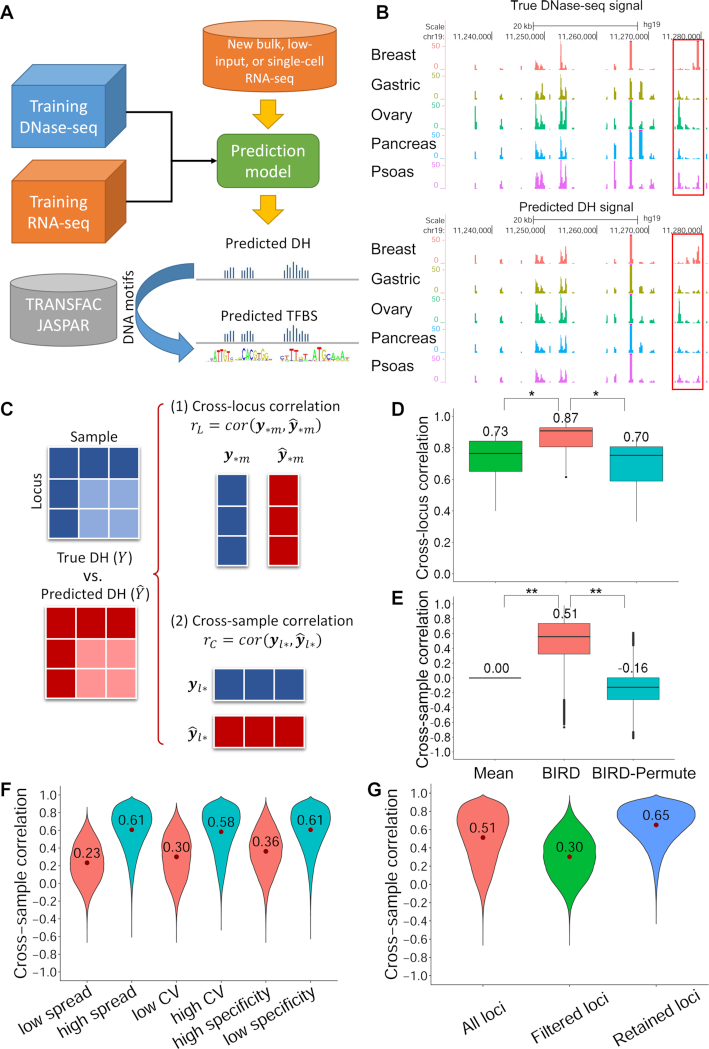
Overview of the study design and BIRD prediction performance using bulk RNA-seq. (**A**) Study design. Bulk DNase-seq and RNA-seq data from Roadmap Epigenomics or ENCODE are used to train BIRD prediction models which are then applied to new RNA-seq samples to predict DH. The predicted DH can be coupled with DNA motifs to predict TFBSs. (**B**) An example of true and predicted DH signals across five different samples. Each track is a sample. Regions highlighted with boxes demonstrate that the predicted DH captures the true DH variation. (**C**) Statistics used to evaluate prediction accuracy. (**D**) Distribution and mean of the prediction-truth correlation *r*_L_ from all samples for three prediction methods: BIRD, random prediction models (‘BIRD-permute’) and the mean DH profile of training data (‘Mean’) in leave-one-cell-type-out cross-validation. *Two-sided Wilcoxon signed-rank test *P*-values < 10^−12^ for comparing two methods (*n* = 70 for each test). (**E**) Distribution and mean of the prediction-truth correlation *r*_C_ for comparing different methods. **Two-sided Wilcoxon signed-rank test *P*-values < 10^−15^ for comparing two methods (*n* = 1 136 465 for each test). (**F**) Distribution and mean of *r*_C_ for different categories of loci. Regulatory elements were divided into two groups (low or high) based on different factors (Supplementary Methods): max-min spread of DH signal, coefficient of variation (CV), or cell-type-specificity. (**G**) Distribution of *r*_C_ for all loci, loci filtered out by factors in (F) (i.e. low spread, low CV or high specificity), and the remaining loci.

Here we investigate the feasibility of predicting regulome using bulk, small-cell-number, and single-cell RNA-seq data (Figure 2A). Although many other functional genomic data types such as histone modification ChIP-seq or DNA methylation data also correlate with and therefore may be used to predict chromatin accessibility (20,21), we focus on using and only using RNA-seq to make predictions because the other data types such as ChIP-seq are much less available and technologies for measuring them in single-cell or low-input samples are either very noisy or unavailable. Thus, prediction based on RNA-seq alone, which has not been explored by previous studies (19,20), has a significantly broader range of applications.

## MATERIALS AND METHODS

### Overall study design

We used matched DNase-seq and RNA-seq samples generated by the Roadmap Epigenomics project (22) (also called Epigenome Roadmap) and the ENCODE project (23) from diverse human cell types to train models for predicting a sample’s genome-wide DH profile using its RNA-seq profile. The models were trained using our recently developed Big Data Regression for predicting DNase I hypersensitivity (BIRD) approach (19) (Supplementary Methods). We then applied the prediction models to independent test cell types not in the training data to predict their DH profiles using bulk, small-cell-number, or single-cell RNA-seq data (Figure 2A). The predicted DH was coupled with transcription factor DNA binding motif information to predict TFBSs. To evaluate the regulome prediction performance, we used DNase-seq, ATAC-seq and TF ChIP-seq data from the test cell types as the gold standard.

### DNase-seq data processing

The aligned DNase-seq data (alignment based on hg19) from 70 Roadmap Epigenomics samples (Dataset S1, representing 30 different cell types) were downloaded from ftp://ftp.genboree.org/EpigenomeAtlas/Current-Release/experiment-sample/Chromatin_Accessibility/ (22). These data were used in the BIRD analysis of bulk RNA-seq, small-cell-number RNA-seq and scRNA-seq for GM12878 and H1.

The aligned DNase-seq data (alignment based on hg19) from 167 ENCODE samples (Dataset S1, representing 74 cell types) were downloaded from https://www.encodeproject.org/ (23). These data were used to train the latest BIRD models in the bone marrow scRNA-seq analysis.

The aligned DNase-seq data for test cell types GM12878 and H1 were downloaded from the ENCODE project (23) (http://hgdownload.cse.ucsc.edu/goldenPath/hg19/encodeDCC/wgEncodeUwDnase).

Details for processing these data are described in Supplementary Methods. Since most genomic loci are noise rather than regulatory elements, we filtered genomic loci to exclude those without strong DH signal in any training DNase-seq sample. The filtering was done similar to (19) (see Supplementary Methods for details).

### Bulk RNA-seq data processing

The aligned RNA-seq data (alignment based on hg19) from the 70 Epigenome Roadmap samples were downloaded from ftp://ftp.genboree.org/EpigenomeAtlas/Current-Release/experiment-sample/mRNA-Seq/. For the 167 ENCODE bulk RNA-seq samples, the ENCODE released gene expression values (FPKM) were downloaded from https://www.encodeproject.org/. GM12878 bulk RNA-seq data (GSM958728) and low-input RNA-seq data from samples with 30 and 100 cells (GSM1087858, GSM1087859, GSM1087856, GSM1087857) were downloaded from GEO. For evaluating the impacts of batch effects, we also obtained bulk RNA-seq data from three different labs for GM12878 (GSM1663002, GSM3612677, GSM958728) and H1 (GSM2680565, GSM1494448, GSM915328). Details for processing these data are described in Supplementary Methods.

### BIRD

The BIRD algorithm was described in detail and systematically evaluated in Zhou *et al.* (19) for exon arrays. For readers’ convenience, it is reviewed in Supplementary Methods andSupplementary Figure S1a-b. BIRD software and trained models (i.e. Epigenome Roadmap model based on 70 samples and ENCODE model based on 167 samples) are available at https://github.com/WeiqiangZhou/BIRD and https://github.com/WeiqiangZhou/BIRD-model.

### Prediction performance evaluation

Prediction performance was evaluated using correlation between the predicted and true signals across all genomic loci within each sample (*r*_L_) and across all samples at each genomic locus (*r*_C_). Let $\hat{y}_{lm}$ be the predicted DH level of locus *l* ( = 1, ..., *L*) in test sample *m* ( = 1, ..., *M*). Let $y_{lm}$be the true DH level measured by DNase-seq. *r*_L_ is the Pearson’s correlation between the predicted signals $\hat{\mathbf {y}}_{*m} = (\hat{y}_{1m},...,\hat{y}_{Lm})^T$ and the true signals $\mathbf {y}_{*m} = (y_{1m},...,y_{Lm})^T$ across different loci for each test sample *m*. *r*_C_ is the Pearson’s correlation between the predicted signals $\hat{\mathbf {y}}_{l*} = (\hat{y}_{l1},...,\hat{y}_{lM})^T$ and the true signals $\mathbf {y}_{l*} = (y_{l1},...,y_{lM})^T$ across different samples for each genomic locus *l*.

### Leave-one-out cross-validation

Leave-one-cell-type-out cross-validation was used to evaluate BIRD prediction accuracy when bulk RNA-seq data were used as predictors (see details in Supplementary Methods).

### Random prediction models by permutation

To construct random prediction models, sample labels of the DNase-seq data in the training dataset were shuffled. This permutation broke the connection between DNase-seq and RNA-seq samples. Then, BIRD was trained by the permuted training dataset and applied to predict DH in the test dataset. The permutation was performed in each fold of the leave-one-out cross-validation and the prediction performance was then evaluated by *r*_L_ and *r*_C_. Note that our permutation did not perturb the locus effects of DH profile. Therefore, predictions from the random prediction models mostly captured the average DH level of each genomic locus across different cell types in the training dataset.

### Wilcoxon signed-rank test

Two-sided Wilcoxon signed-rank test (24) was performed to obtain *P*-values for comparing prediction accuracy of BIRD, random prediction models and the mean DH profile (Figure 2D and E). In order to test whether two methods perform equally, the paired *r*_L_ values from these two methods for each test sample was obtained. Then the *r*_L_ pairs from all samples were used for Wilcoxon signed-rank test. Similarly, the paired *r*_C_ values from these two methods for each locus was obtained. Then the *r*_C_ pairs from all loci were used for Wilcoxon signed-rank test.

### Bulk ATAC-seq data processing

ATAC-seq data for GM12878 with 50 000 and 500 cells were obtained from GEO (GSE47753). ATAC-seq data for hematopoietic cell types (CMP, GMP, MEP) in the bone marrow example were downloaded from GEO (GSE74912). Details for data processing are described in Supplementary Methods.

### Transcription factor binding site prediction

BIRD predicted chromatin accessibility was coupled with DNA motifs to predict binding sites for 16 TFs in GM12878 cells and 10 TFs in H1 cells. Similarly, we predicted binding activities of three lineage-specific TFs in the bone marrow scRNA-seq analysis. Details are described in Supplementary Methods.

### Histone modification ChIP-seq and MOWChIP-seq data processing

H3K27ac and H3K4me3 MOWChIP-seq data for GM12878 with 100 and 600 cells were obtained from GEO (GSE65516: GSM1666202, GSM1666203, GSM1666204, GSM1666205, GSM1666206, GSM1666207, GSM1666208, GSM1666209). Data processing details are described in Supplementary Methods.

### Applying BIRD to single-cell RNA-seq data

The scRNA-seq data used in this study and the platforms used to generate these data are summarized in Supplementary Table S1. The data generated using the 10x Genomics or other similar droplet-based scRNA-seq platforms are highly sparse. For these data, we first apply scVI (25), a deep-learning based imputation method, to reconstruct cells’ gene expression values. BIRD is then applied to quantile normalize (26) the scVI-imputed gene expression profiles with the training RNA-seq data and then predict chromatin accessibility. For such sparse scRNA-seq data, our analyses in Supplementary Figure S10 show that imputation can greatly improve the prediction accuracy compared to applying BIRD without imputation.

For scRNA-seq data generated by other platforms that are relatively low throughput in terms of cell number but with denser data per cell (e.g. Fluidigm, micropipette), we directly use the non-imputed gene expression values as the input for BIRD. BIRD will quantile normalize the scRNA-seq gene expression profiles with the training RNA-seq data and then predict chromatin accessibility. For these data, our analyses in Supplementary Figure S7 show that imputation does not clearly improve the prediction accuracy.

### Predicting chromatin accessibility in GM12878 and H1 using single-cell RNA-seq data

We downloaded two datasets from GEO: (1) GM12878 scRNA-seq data (GSE44618, 28 cells in total), (2) H1 scRNA-seq data (GSE64016, 62 cells in total). For these samples, reads were mapped to human genome hg19 using Tophat (27). Gene expression values were then computed using Cufflinks (28) in the same way as how we processed the Epigenome Roadmap RNA-seq data.

For each dataset, we randomly sampled *k* cells (*k* = 1, 5, 10, 20, 28 for GM12878; *k* = 1, 5, 10, 20, 50, 62 for H1) and calculated their average gene expression profile. The average gene expression profile was then used as the input for BIRD to predict the DH profile. For each *k* (except for *k* = 1 and 28 for GM12878, and *k* = 1 and 62 for H1), the random sampling was repeated 10 times. The mean and standard deviation (SD) of the results from the 10 analyses were shown in Figure 5C, D and Supplementary Figure S6. For *k* = 1, the analysis was performed for every single cell.

### Measuring chromatin accessibility in GM12878 and H1 using single-cell ATAC-seq data

The scATAC-seq data used in this study and the platforms used to generate these data are also summarized in Supplementary Table S1. Two scATAC-seq datasets for GM12878 were obtained. Dataset 1 (ATAC1) was obtained from GEO (GSM1647121). This dataset was a mixture of human GM12878 cells and mouse Patski cells. Paired-end reads were trimmed by Trimmomatic (29) to remove adaptor content and aligned to human genome hg19 using bowtie2 (30) with parameter -X 2000. PCR duplicates were removed using Picard (http://broadinstitute.github.io/picard/). The aligned reads were then assigned to individual cells based on the barcode information and only GM12878 cells were retained for subsequent analyses. For each cell, bin-level fragment coverage was obtained for each genomic locus (i.e. 200 bp bin), and bin fragment coverage was normalized in the same way as the bulk ATAC-seq data. The scATAC-seq data are highly discrete. According to the original report describing this data (9), the sequencing has reached saturation and the median value of total read counts per cell was 2503. We identified GM12878 cells (*n* = 222) with more than 500 non-zero-coverage loci and used them for subsequent analyses.

Dataset 2 (i.e. ATAC2) was obtained from GEO (GSE65360). This dataset contains GM12878 scATAC-seq for 384 single cells. For each single cell, paired-end reads were trimmed by Trimmomatic to remove adaptor content and aligned to human genome hg19 using bowtie2 with parameter -X 2000. PCR duplicates were removed using Picard. Then, bin-level fragment coverage for each cell was computed, normalized and transformed in the same way as scATAC-seq dataset 1. Finally, 340 cells with >500 non-zero-coverage loci were retained for subsequent analyses.

For the scATAC-seq dataset 1, we randomly sampled a group of *k* cells (*k* = 1, 5, 10, 20, 28, 50, 100, 222) and calculated their average scATAC-seq profile (i.e. average of the normalized bin fragment coverage). The average profile was then log2-transformed after adding a pseudocount of 1. For each *k* (except for *k* = 1 and 222), we repeated the random sampling 10 times. The mean and SD of results from the 10 analyses were shown in Figure 5C. For *k* = 1, the analysis was performed on every single cell. The same analysis was also performed for the scATAC-seq dataset 2 with *k* cells (*k* = 1, 5, 10, 20, 28, 50, 100, 222, 340).

For H1, a scATAC-seq dataset with 96 cells was downloaded from GEO (GSE65360) and processed in the same way as the GM12878 scATAC-seq dataset 2 (i.e. ATAC2). We retained 90 cells with >500 non-zero-coverage loci for subsequent analysis. We performed similar analysis with *k* cells (*k* = 1, 5, 10, 20, 50, 62) as above. In all H1 related analyses, BIRD model was retrained using bulk DNase-seq and RNA-seq data from 65 samples after excluding all H1 related samples from the 70 Epigenome Roadmap samples.

### Hybrid prediction based on combining single-cell RNA-seq and single-cell ATAC-seq

For the hybrid approach in the GM12878 example, we randomly sampled *x* (*x* = 22, 72, 194, 312) cells from the scATAC-seq dataset 2. Dataset 2 was used since it had higher data quality than dataset 1 based on analyses in Figure 5C. We obtained the average scATAC-seq profile of the sampled cells using the protocol described above. We also obtained BIRD-predicted DH from pooled scRNA-seq using 28 cells. The average of the ATAC-seq profile and BIRD predicted DH profile was then computed. The total number of cells used by this hybrid approach was *k* = *x* + 28 (i.e. *k* = 50, 100, 222, 340). In Figure 5C, this hybrid approach was compared to pooled scATAC-seq using the same number of cells. For the hybrid approach, the sampling of cells from scATAC-seq was repeated 10 times. The mean and SD of results from the 10 analyses were shown in Figure 5C.

### Predicting differential chromatin accessibility between GM12878 and H1 using single-cell RNA-seq

BIRD prediction model was trained using 65 Epigenome Roadmap samples after excluding all GM12878 or H1 related samples (i.e. GM12878 and H1 were not in the training data). For each cell type (i.e. GM12878 and H1), we randomly sampled *k* cells (*k* = 1, 5, 10, 20) and calculated the average gene expression profile. Then, BIRD was applied to predict DH of each cell type using the corresponding average gene expression profile. For comparison, the same number of cells were pooled from scATAC-seq data for GM12878 and H1. To evaluate the performance of different methods for predicting differential chromatin accessibility, differential signals obtained from bulk DNase-seq data between GM12878 and H1 were used as the gold standard (i.e. true difference). The performance of each method (i.e. BIRD and scATAC-seq) for predicting differential signals was calculated as the Pearson’s correlation between the predicted difference and true difference in DH values of the two cell types. The analysis was applied to all DHSs and differential DHSs, respectively. Differential DHSs were defined as DHSs with |true log_2_-scale DH difference between the two cell types| > 1, after filtering out loci with log2 DH level smaller than 1 in both cell types. The random sampling was repeated 10 times for each *k* and the results were shown in Figure 7A and B. The scatterplots in Figure 7C–E compare the BIRD predicted differential signals based on pooling 1, 5 and 20 cells from scRNA-seq with the true differential signals. The scatterplots in Figure 7F–H compare the differential signals obtained by pooling 1, 5 and 20 cells from scATAC-seq with the true differential signals.

### Analyses of the bone marrow single-cell RNA-seq data

The 10x Genomics human bone marrow scRNA-seq data from two different donors (BM1 and BM6) were downloaded from the Human Cell Atlas website (https://preview.data.humancellatlas.org/). This dataset was added during the revision of this article. During this period, BIRD has been updated by retraining the model using the 167 ENCODE DNase-seq and RNA-seq samples. The latest BIRD trained using the ENCODE data therefore was used here. We applied scVI (25) to the scRNA-seq data to impute gene expression. BIRD was then applied to the imputed gene expression to make predictions.

For evaluation, because the cell type label for each cell is unknown, we used the bulk RNA-seq data from the fluorescence-activated cell sorting (FACS) purified hematopoietic cell types (31) (GEO: GSE74246) to computationally annotate cells’ cell type. Three cell types (CMP, GMP, MEP) were found to be unambiguously and consistently annotated in both donors and had scATAC-seq and bulk ATAC-seq data for evaluation. Thus, they were used for our benchmark analysis.

In order to compare BIRD and scATAC-seq, bulk ATAC-seq data (31) from CMP, GMP and MEP were downloaded from GEO (GSE74912) as the gold standard. scATAC-seq data (32) for the same cell types were downloaded from GEO (GSE96772) and were processed similar to the scATAC-seq dataset 2 (i.e. ATAC2) in the previous sections. The benchmark comparison was conducted similar to the analysis of GM12878 and H1 scRNA-seq data.

Details of the data processing and analysis for this example are described in Supplementary Methods.

## RESULTS

### Predicting chromatin accessibility using bulk RNA-seq

We begin with evaluating the feasibility of using bulk RNA-seq to predict DH. We downloaded DNase-seq and matching RNA-seq data for 70 human samples representing 30 different cell types (Dataset S1) from the Roadmap Epigenomics project (22). After preprocessing and normalization, 37 335 transcripts with expression measurements from RNA-seq and 1 136 465 genomic loci with DH measurements from DNase-seq were obtained and served as predictors and responses, respectively (Materials and Methods). Our goal is to predict DH at these 1 136 465 loci, also referred to as DNase I hypersensitive sites (DHSs), using the 37 335 predictors. We evaluate the prediction using leave-one-out cross-validation. In each fold of the cross-validation, the 30 cell types were partitioned into a training dataset consisting of 29 cell types and a test dataset consisting of 1 cell type. BIRD (19) (Supplementary Figure S1A and B) prediction models were trained using samples in the training data and then applied to RNA-seq samples in the test data to predict DH. Prediction performance was evaluated by comparing the predicted DH values with the true DH values measured by DNase-seq. Figure 2B shows that the predicted DH values were able to capture the observed variation in the real DNase-seq data.

In order to more systematically evaluate the prediction accuracy, we computed two types of correlation: (i) Pearson’s correlation between the predicted and true DH values across all genomic loci within each sample (‘cross-locus correlation’ *r*_L_), and (ii) Pearson’s correlation between the predicted and true DH values across all samples at each genomic locus (‘cross-sample correlation’ *r*_C_) (Figure 2C). As a control, we trained random prediction models (‘BIRD-Permute’) by permuting the link between the DNase-seq and RNA-seq samples in the training data and then applied these random prediction models to the test data.

Comparisons between BIRD and BIRD-Permute show that BIRD prediction based on RNA-seq significantly outperformed random prediction models (Figure 2D and E). The mean cross-locus and cross-sample correlation between the BIRD-predicted and true DH values were *r*_L_ = 0.87 and *r*_C_ = 0.51 respectively (Figure 2D and E). These results are consistent with our previously reported prediction accuracy based on exon arrays where the mean *r*_L_ and *r*_C_ were 0.82 and 0.50 respectively (19).

Note that *r*_L_ has a larger mean than *r*_C_. As discussed in (19), this is because the baseline DH levels of different regulatory elements are different. Some loci are more active than others in most cell types (Supplementary Figure S1C). Due to these locus-effects, one can predict cross-locus DH variation in a new sample to a large extent by simply using the mean DH profile (‘Mean’) across training samples (Figure 2D, mean *r*_L_ = 0.73). However, the mean DH profile cannot predict how DH varies across samples (Figure 2E, *r*_C_ = 0 since *r*_C_ is computed within each locus and not affected by locus-effects). The permutation used in BIRD-Permute does not disrupt the locus-effects. Thus, BIRD-Permute also has relatively large *r*_L_ and relatively small *r*_C_ (Figure 2D and E: mean *r*_L_ versus *r*_C_ = 0.70 versus -0.16). In summary, for BIRD-permute and the mean DH approach, *r*_L_ and *r*_*C*_ reflect the ability of locus-effects to predict DH variation across loci and samples respectively. For BIRD, sample-dependent transcriptome information is used to make prediction. Thus, its *r*_L_ represents the joint effect of two factors: (i) how well the sample-independent locus-effects explain DH variation, and (ii) how well the sample-dependent gene expression information predicts changes of DH across samples. By contrast, the *r*_C_ of BIRD only reflects its ability to predict changes of DH across samples. Since the main difference between *r*_L_ and *r*_C_ is the inclusion and exclusion of the locus-effects which influence all compared methods in a similar way, using *r*_L_ or *r*_C_ does not change the relative ranking of different methods (i.e. whether BIRD is better than the other methods or not). For this reason, in our subsequent analyses (e.g. small-cell-number and single-cell analyses) where *r*_C_ cannot be computed due to lack of multiple test samples, one can still rely on *r*_L_ to determine which method is better for predicting changes of DH across samples.

Consistent with the observations in (19), cross-sample prediction accuracy varies substantially among different loci. While 5% of loci had *r*_C_ <0, 57% and 23% of loci had *r*_C_ >0.5 and >0.75 respectively. Thus, DH can be predicted with moderate to high accuracy for a substantial fraction of loci. Loci with narrow signal range (characterized by the difference between the maximal and minimal DH values), low signal variability (characterized by coefficient of variation (CV)), or high cell-type-specificity (characterized by the number of cell types in which the locus is active or inactive) tend to have lower *r*_C_ (Figure 2F, Supplementary Methods). Excluding these loci would increase cross-sample prediction accuracy. For instance, when we filtered out loci with low signal range, low CV and high cell-type-specificity using the predicted DH in test samples, the mean *r*_C_ for the remaining loci increased from 0.51 to 0.65, and 80% and 37% of loci had *r*_C_ >0.5 and >0.75 respectively (Figure 2G).

Next, we compared BIRD prediction with predictions made by ChromImpute (20). BIRD prediction was based on RNA-seq data only. By contrast, ChromImpute used multiple functional genomic data types jointly as predictors. These predictors were selected by ChromImpute as the Epigenome Roadmap data types most informative for predicting DH (20). They include multiple histone modifications such as H3K4me1, H3K4me3, H3K36me3, H3K27me3, H3K9me3, H3K27ac and H3K9ac. Since many of these histone modifications overlap with DNase I hypersensitive sites (DHSs) in the genome and they are more directly correlated with chromatin states, one would expect that they are more informative than RNA-seq for predicting DH. However, BIRD predictions based on RNA-seq alone were only slightly less accurate than ChromImpute predictions based on multiple functional genomic data types. Both these methods substantially outperformed predictions based on the mean DH profile of training samples (Supplementary Methods, Supplementary Figure S2). Since gene expression data are easier to collect than ChIP-seq data for multiple histone modifications, using RNA-seq alone to make predictions would have a much broader range of applications in practice.

We also assessed how batch effects may affect BIRD prediction accuracy. For GM12878 and H1, we collected bulk RNA-seq samples generated by three different labs, respectively. Supplementary Figure S3 shows that RNA-seq samples from different labs produced similar prediction accuracy. The difference in BIRD prediction accuracy between labs was much smaller than the difference in prediction accuracy between BIRD and other methods (Supplementary Figure S3), suggesting that BIRD is relatively robust to batch effects.

Collectively, analyses in this section show that predicting chromatin accessibility using bulk RNA-seq is feasible. The prediction accuracy based on RNA-seq was highly consistent with our previously reported prediction accuracy based on exon arrays (19). As demonstrated in (19), predictions with this accuracy are practically useful for a variety of applications including predicting TFBSs, predicting differential regulatory activities, and turning publicly available gene expression data into a regulome database. Details of these applications can be found in (19). In the interest of space, similar analyses will not be repeated here. Below we will instead focus on evaluating the possibility of making prediction using low-input and single-cell RNA-seq data which has not been studied before.

### Predicting chromatin accessibility using Small-cell-number RNA-seq

**Figure 3.**
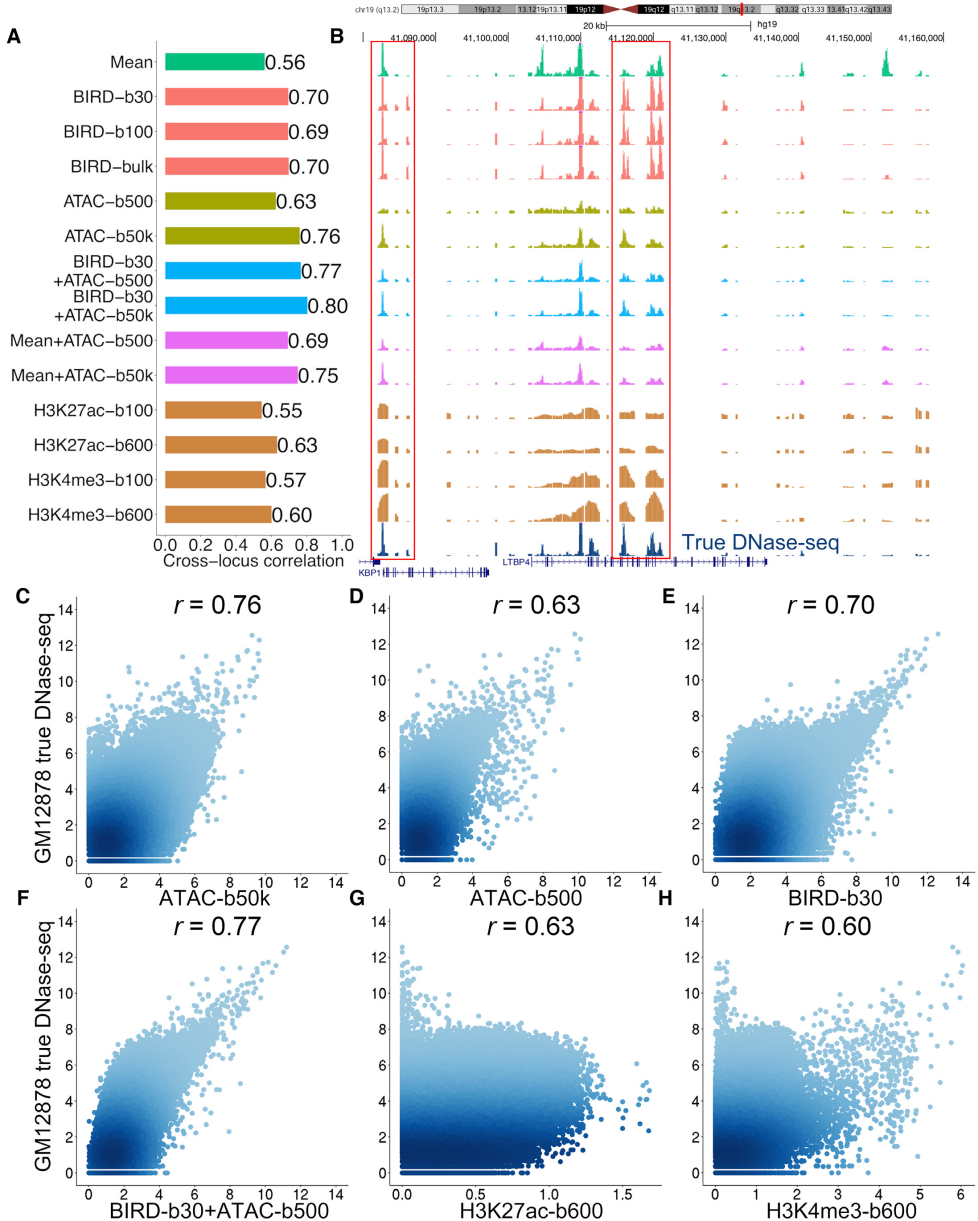
Predicting DH using small-cell-number RNA-seq data. (**A**) Correlation between the bulk GM12878 DNase-seq signal and chromatin accessibility predicted or measured by different methods. ‘Mean’: mean DH profile of training samples. ‘BIRD-b30’, ‘BIRD-b100’: BIRD-predicted DH based on small-cell-number RNA-seq samples with 30 and 100 cells. ‘BIRD-bulk’: BIRD-predicted DH based on bulk RNA-seq. ‘ATAC-b500’, ‘ATAC-b50k’: ATAC-seq with 500 and 50 000 cells. ‘BIRD-b30+ATAC-b500’, ‘BIRD-b30+ATAC-b50k’: average of BIRD-predicted DH from 30 cells and ATAC-seq from 500 or 50 000 cells. ‘Mean+ATAC-b500’, ‘Mean+ATAC-b50k’: average of mean DH profile of training samples and ATAC-seq from 500 or 50 000 cells. ‘H3K27ac-b100’, ‘H3K27ac-b600’, ‘H3K4me3-b100’ and ‘H3K4me3-b600’: MOWChIP-seq for histone modification H3K27ac or H3K4me3 with 100 or 600 cells. (**B**) An example that compares chromatin accessibility predicted or measured by different methods. True bulk DNase-seq signal is shown on the bottom track as a reference. Regions highlighted by boxes illustrate that BIRD predicted DH better than ‘Mean’ and ‘ATAC-b500’. (**C**–**H**) Scatterplots comparing true bulk DNase-seq signal with chromatin accessibility predicted or measured by ATAC-b50k, ATAC-b500, BIRD-b30, BIRD-b30+ATAC-b500, H3K27ac-b600 and H3K4me3-b600. Each dot is a genomic locus. Pearson’s correlation coefficient is shown on top of each plot.

Our next question is whether BIRD trained using bulk RNA-seq data can be applied to RNA-seq generated from small-cell-number samples to predict DH. We obtained published RNA-seq data from low-input GM12878 lymphoblastoid samples with 30 and 100 cells as well as bulk samples (3). BIRD trained using the Epigenome Roadmap data were applied to each test sample. GM12878 was not in the training samples. For evaluation, we did not have the true chromatin accessibility profile for each low-input sample. However, according to statistical theory, if cells in a small-cell-number sample are randomly drawn from a bulk cell population, the mean DH profile of the small-cell-number sample and that of the bulk sample should have the same expectation. Therefore, one can use the bulk GM12878 DNase-seq data from the ENCODE (23) as the ‘truth’. Based on this gold standard, we compared BIRD predictions with GM12878 ATAC-seq from 500 and 50 000 cells. It turns out that ATAC-seq from 50 000 cells (‘ATAC-b50k’) showed the highest correlation with the true DNase-seq signal (Figure 3A and B, *r*_L_ = 0.76). Surprisingly, however, BIRD-predicted DH signals from 30 and 100 cells consistently predicted the truth better than ATAC-seq from 500 cells (Figure 3A and B, *r*_L_ = 0.63, 0.70 and 0.69 for ‘ATAC-b500’, ‘BIRD-b30’ and ‘BIRD-b100’). Note that using the mean DH profile from the training data alone was able to predict DH to certain degree (Figure 3A and B, *r*_L_ = 0.56 for ‘Mean’). The prediction accuracy of BIRD based on a small number of cells was similar to that based on bulk RNA-seq (Figure 3A and B, *r*_L_ = 0.70 for ‘BIRD-bulk’). Figure 3B provides an example illustrating signals from different methods.

Interestingly, combining the ATAC-seq signal from 500 cells and the BIRD-predicted DH from 30 cells by average (530 cells used in total) allowed one to better predict the gold standard DNase-seq signal (Figure 3A and B, ‘BIRD-b30+ATAC-b500’, *r*_L_ = 0.77). Using only 530 cells, the combined signal achieved slightly better accuracy than ATAC-seq using 50 000 cells (*r*_L_ = 0.76), and it was better than using BIRD-b30 (*r*_L_ = 0.70) or ATAC-b500 (*r*_L_ = 0.63) alone (Figure 3C–F). Similarly, by averaging ATAC-seq from 50 000 cells and BIRD predictions from 30 cells, we were able to predict the gold standard better than ATAC-b50k (Figure 3A and B, *r*_L_ = 0.80 for ‘BIRD-b30+ATAC-b50k’). The same improvement was not observed when the BIRD prediction was replaced by the prediction based on the mean DH profile (Figure 3A and B, *r*_*L*_ = 0.69 and 0.75 for ‘Mean+ATAC-b500’ and ‘Mean+ATAC-b50k’). These results show that DH predicted from small-cell-number RNA-seq can be integrated with small-cell-number ATAC-seq data (BIRD+ATAC-seq) to obtain better signal.

We repeated the above evaluation by using ATAC-seq from 50 000 cells to replace bulk DNase-seq to serve as the gold standard. Similar conclusions were obtained (Supplementary Figure S4). Unlike the DNase-seq gold standard which came from a study different from the studies that generated the test ATAC-seq and RNA-seq data, the ATAC-50k gold standard was collected from the same study as the ATAC-b500 data (RNA-seq was from a different study). Thus, the ATAC-50k gold standard should intrinsically favor ATAC-b500 over BIRD due to potential lab effects. Despite this, BIRD predictions based on 30 and 100 cells performed close to ATAC-b500 in this comparison, and BIRD-b30+ATAC-b500 outperformed ATAC-b500 (Supplementary Figure S4).

### Predicting transcription factor binding sites (TFBSs) using Small-cell-number RNA-seq

**Figure 4.**
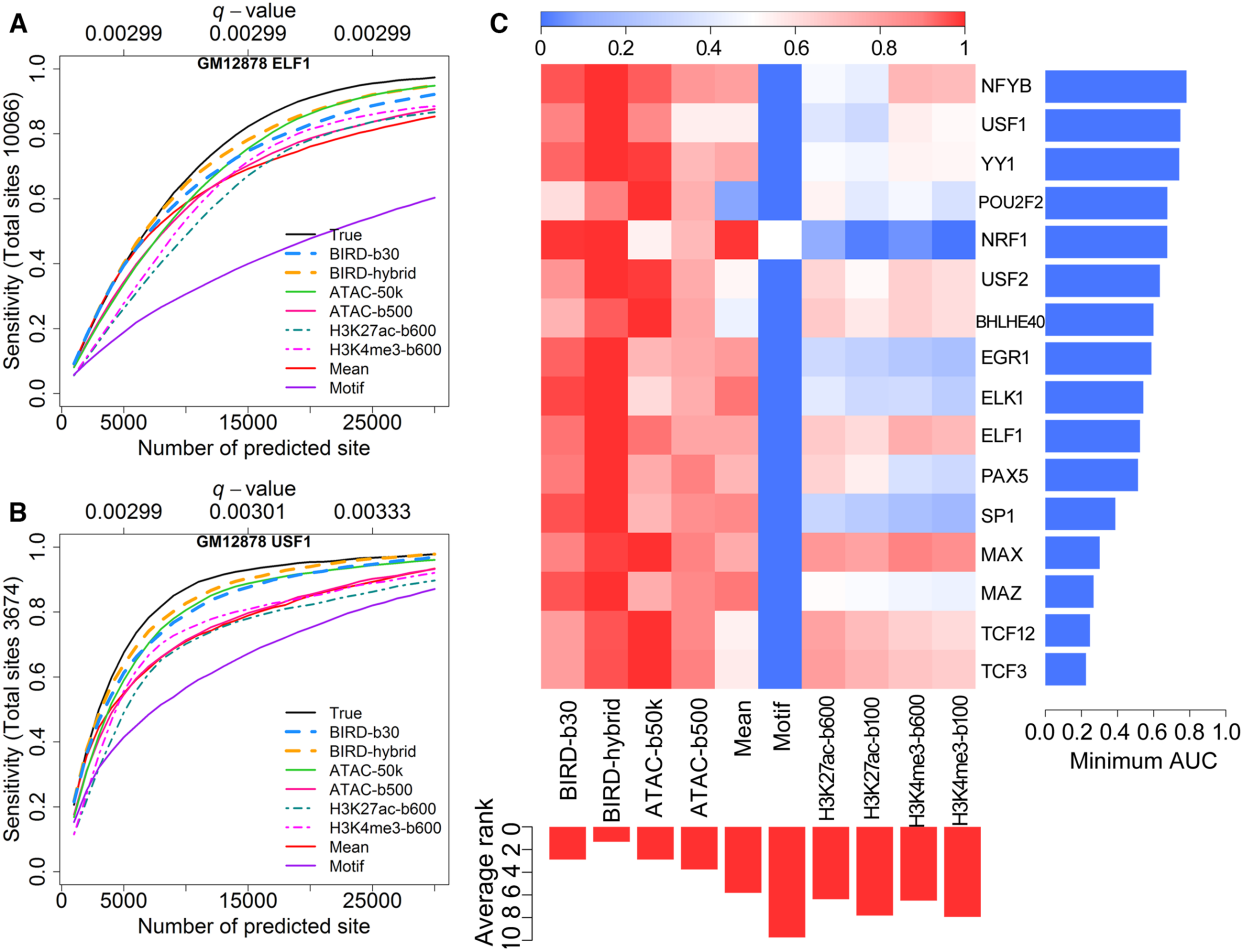
Predicting TFBSs using small-cell-number RNA-seq data. (**A**, **B**) Sensitivity-rank curve for predicting ELF1 and USF1 binding sites in GM12878 using true DNase-seq (‘True’), ATAC-seq from 500 or 50 000 cells (‘ATAC-b500’, ‘ATAC-b50k’), mean DH profile of training samples (‘Mean’), motif mapping score (‘Motif’), BIRD-predicted DH using 30 cells (‘BIRD-b30’), the average of BIRD-predicted DH using 30 cells and ATAC-seq using 500 cells (‘BIRD-hybrid’), and MOWChIP-seq for H3K27ac and H3K4me3 using 600 cells (‘H3K27ac-b600’, ‘H3K4me3-b600’). The performance for MOWChIP-seq using 100 cells was generally worse than using 600 cells and hence is not shown here for clarity of display. The *q*-values for BIRD-b30 predictions are shown on the top of each plot. (C) Scaled area under the curve (AUC) for different methods in TFBS prediction (Supplementary Methods). Each row is a TF, and each column is a method. For each TF, different methods are ranked based on the AUC value, and the worst AUC value of all methods is shown on the right using a blue bar. The average rank of each method across all TFs is shown on the bottom using a red bar. Smaller rank means better performance.

We further evaluated whether coupling DH predicted using low-input RNA-seq with DNA motif information can predict TFBSs. We predicted TFBSs for 16 TFs in GM12878. The DNA motif of each TF was mapped to the genome, and motif sites with high predicted DH were identified and ranked as predicted TFBSs. For each TF, the corresponding ChIP-seq data were obtained from ENCODE (23). Motif-containing ChIP-seq peaks were used as the gold standard to evaluate the prediction accuracy (Supplementary Methods). For comparison, we made predictions using BIRD-b30, BIRD-hybrid (i.e. ‘BIRD-b30+ATAC-b500’), ATAC-b50k, ATAC-b500, mean DH profile of the training samples (‘Mean’), true DNase-seq (‘True’, positive control), and motif site alone (‘Motif’, negative control). Figure 4A and B shows the fraction of gold standard TFBSs (i.e. sensitivity) that were discovered by the top predicted sites. BIRD recovered a substantial fraction of the true TFBSs. Take USF1 as an example. The top 5000 predictions by BIRD-b30 (DHS *q*-value < 0.003, Supplementary Methods) recovered 61% of the gold standard USF1 binding sites (Figure 4B). As a comparison, the top 5000 predictions based on true DNase-seq (positive control) and motif only (negative control) covered 68% and 41% of the gold standard USF1 binding sites respectively. For the other methods, this percentage was 59% for ATAC-50k, 64% for BIRD-hybrid, 55% for ATAC-500, and 55% for the mean DH profile.

To facilitate method comparison, we calculated the area under the curve (AUC) for each method, normalized by dividing the AUC of the ‘True’ DNase-seq (Figure 4C, Supplementary Methods). Comparison of the normalized AUC shows that BIRD-b30 outperformed the mean DH and motif only methods in all 16 tested TFs. Furthermore, BIRD-b30 outperformed ATAC-b500 in 11 of 16 TFs (Figure 4C). Interestingly, BIRD-hybrid (BIRD-b30+ATAC-b500) outperformed ATAC-b500 in all 16 TFs, and outperformed ATAC-b50k in 11 of 16 TFs. Thus, DH predicted by BIRD from 30 cells more accurately predicted TFBSs than ATAC-seq from 500 cells, and combining BIRD predictions based on low-input RNA-seq with low-input ATAC-seq better predicted TFBSs than bulk ATAC-seq.

### A comparison of BIRD and Low-input technologies ATAC-seq and MOWChIP-seq

Next, we compared DH predicted by BIRD using 30 cells, ATAC-seq, and histone modification H3K27ac and H3K4me3 profiles measured by MOWChIP-seq using 100 and 600 GM12878 cells. Since signal span of histone modifications is different from that of DNase-seq and ATAC-seq due to nucleosome displacement around TFBSs (33), we first optimized the parameter for analyzing MOWChIP-seq data (Supplementary Methods, Supplementary Figure S5A–C). The comparisons below are based on the optimal MOWChIP-seq performance. It was observed that predictions or measurements for each data type correlated better with the bulk data from the same data type than the bulk data from other data types (Supplementary Figure S5D). For instance, H3K27ac MOWChIP-seq using 100 and 600 cells (H3K27ac-b100 and H3K27ac-b600) performed better than BIRD-b30 when H3K27ac bulk ChIP-seq was used as gold standard for evaluation, but the same MOWChIP-seq data performed worse than BIRD-30 when bulk DNase-seq was used as gold standard (Supplementary Figure S5D, Figure 3A, B, G, H). This suggests that there were substantial differences among data types, making a fair comparison difficult.

For predicting TFBSs, however, the comparison was based on TF ChIP-seq and therefore relatively fair. Both BIRD-b30 and ATAC-b500 substantially outperformed MOWChIP-seq in terms of the overall performance in all 16 tested TFs (Figure 4). Among the MOWChIP-seq data, H3K27ac-b600 had the best overall performance for predicting TFBSs (Figure 4C). BIRD-b30, ATAC-b500, and BIRD-hybrid (BIRD-b30+ATAC-b500) outperformed H3K27ac-b600 in all 16 tested TFs.

### Predicting chromatin accessibility and TFBSs Using Single-cell RNA-seq

**Figure 5.**
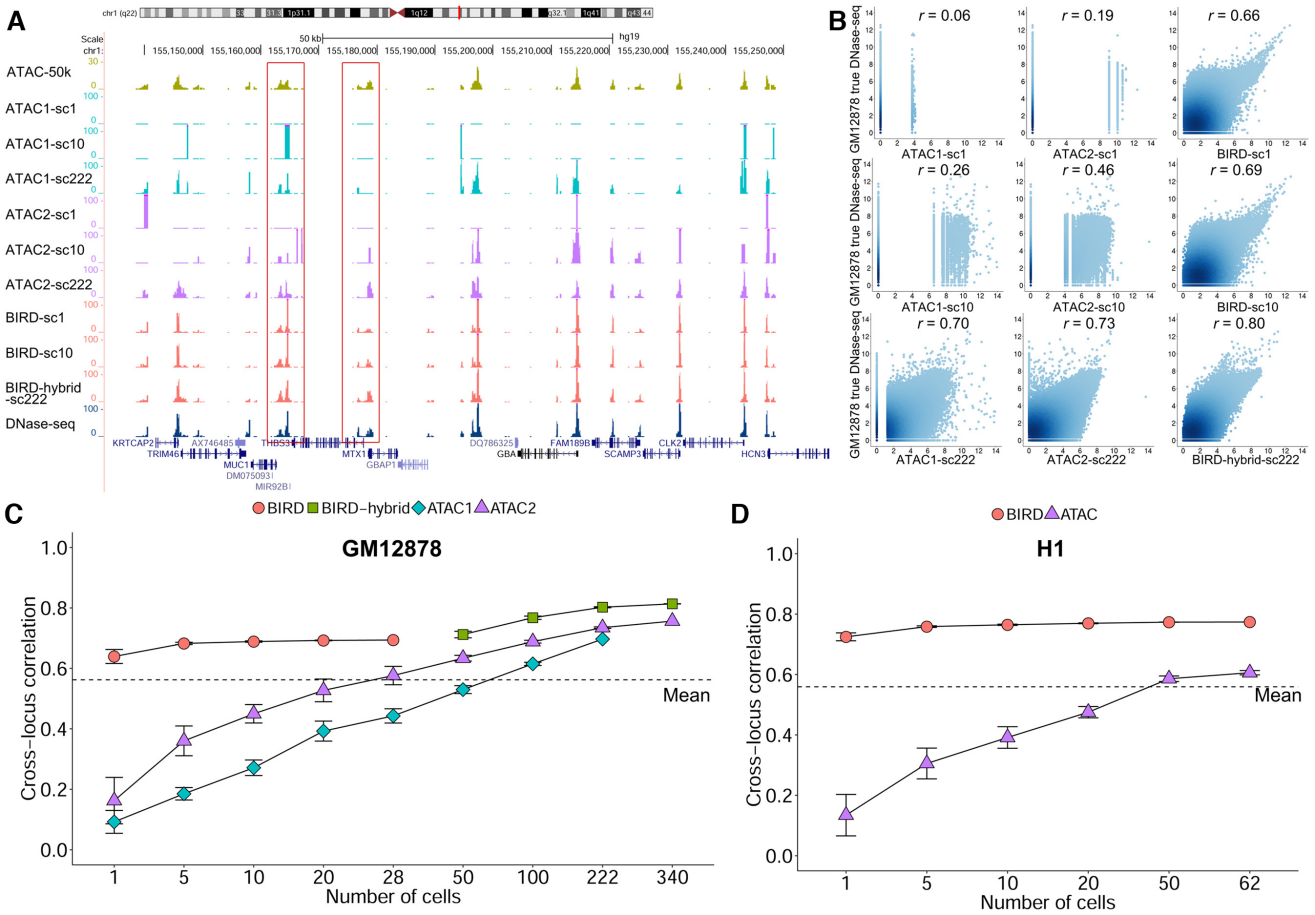
Predicting chromatin accessibility using single-cell RNA-seq data. (**A**) An example comparing chromatin accessibility reported by different single-cell methods for GM12878. ‘ATAC1-sc1’, ‘ATAC1-sc10’ and ‘ATAC1-sc222’: single-cell ATAC-seq from 1 cell, pooled 10 or 222 cells using scATAC-seq dataset 1. ‘ATAC2-sc1’, ‘ATAC2-sc10’ and ‘ATAC2-sc222’: single-cell ATAC-seq from 1 cell, pooled 10 or 222 cells using scATAC-seq dataset 2. ‘BIRD-sc1’, ‘BIRD-sc10’: BIRD-predicted DH based on single-cell RNA-seq data from 1 cell or pooled 10 cells. ‘BIRD-hybrid-sc222’: the average of BIRD-predicted DH with 28 cells and single-cell ATAC-seq from 194 cells using scATAC-seq dataset 2. As references, bulk ATAC-seq from 50 000 cells (‘ATAC-b50k’) and DNase-seq are shown on the top and bottom respectively. (**B**) Scatterplots comparing true bulk DNase-seq signal with chromatin accessibility obtained by ATAC1, ATAC2 and BIRD (or BIRD-hybrid for 222 cells) using 1 cell, pooled 10 or 222 cells for GM12878. Each dot is a genomic locus. Pearson’s correlation coefficient is shown on top of each plot. (**C**) Pearson’s correlation between the true bulk DNase-seq signal and chromatin accessibility obtained by different single-cell methods for GM12878. The correlation is shown as a function of the pooled cell number. ‘ATAC1’: scATAC-seq dataset 1. ‘ATAC2’: scATAC-seq dataset 2. ‘BIRD’: BIRD-predicted DH using pooled single-cell RNA-seq. ‘BIRD-hybrid’: the average of BIRD-predictions based on 28 cells and pooled ATAC-seq from scATAC-seq dataset 2 (here x-axis is the total number of cells used by scRNA-seq and scATAC-seq). (**D**) Pearson’s correlation between the true bulk DNase-seq signal and chromatin accessibility obtained by BIRD or scATAC-seq for H1. In (C) and (D), prediction performance using the mean DH profile of training samples (‘Mean’) is shown as a dashed line. Error bars are standard deviation based on 10 independent samplings of cells (Methods).

We proceeded to ask whether one can use single-cell RNA-seq (scRNA-seq) data to predict DH. We first analyzed a scRNA-seq dataset with 28 single cells for GM12878. In this dataset (3), single cells were isolated using a glass micropipette (Supplementary Table S1). After calculating gene expression for each cell, we pooled *k* (*k* = 1, 5, 10, 20, 28) cells randomly drawn from the dataset together and used their average expression profile to predict DH based on BIRD models trained from the Epigenome Roadmap bulk RNA-seq data. For comparison, we analyzed published single-cell ATAC-seq data in GM12878 generated by two different laboratories (‘ATAC1’ (9): 222 cells obtained using combinatorial cellular indexing; ‘ATAC2’ (10): 340 cells obtained using Fluidigm C1 microfluidics chips) (Supplementary Table S1). We computed average scATAC-seq profile for *k* (*k* = 1, 5, 10, 20, 28, 50, 100, 222, 340) cells randomly drawn from each dataset respectively. Figure 5A–C shows the performance of different methods evaluated using bulk DNase-seq as the gold standard. Holding the cell number the same, BIRD based on pooled scRNA-seq was consistently better than pooled scATAC-seq for predicting bulk DNase-seq (Figure 5B and C). Interestingly, BIRD predictions were always more accurate than predictions based on the mean DH profile, while pooled scATAC-seq using ≤50 cells from ATAC1 or ≤20 cells from ATAC2 were less accurate than predictions based on the mean DH profile (Figure 5C). Prediction accuracy increased as more cells were pooled together. BIRD predictions based on a single cell was comparable to pooling 100 cells from ATAC1 or pooling 50 cells from ATAC2. The results remained similar when the gold standard was changed to bulk ATAC-seq data from 50 000 or 500 cells (Supplementary Figure S6).

We also combined BIRD predictions based on pooling scRNA-seq from 28 cells with the pooled scATAC-seq profile from *x* cells (*x* = 22, 72, 194, 312) by taking the average of the two profiles (‘BIRD-hybrid’). We then compared BIRD-hybrid with pooled scATAC-seq data using the same number of cells (i.e. *k* = 28 + *x* = 50, 100, 222, 340). BIRD-hybrid also outperformed pooled scATAC-seq (Figure 5A–C, Supplementary Figure S6).

We applied BIRD to another scRNA-seq dataset with 62 human embryonic stem cells (H1) (34). In this dataset, single cells were collected using Fluidigm C1 microfluidics chips (Supplementary Table S1). H1 was not in the training data. We pooled *k* = 1, 5, 10, 20, 50, and 62 cells for analysis. For comparison, we analyzed scATAC-seq data in H1 from (10) by pooling the same number of cells (Figure 5D, ‘ATAC’). These scATAC-seq cells were also obtained using Fluidigm C1 microfluidics chips. Bulk DNase-seq data in H1 was used as the gold standard for evaluation. BIRD predictions were consistently better than the mean DH profile and scATAC-seq by pooling the same number of cells (Figure 5D).

**Figure 6.**
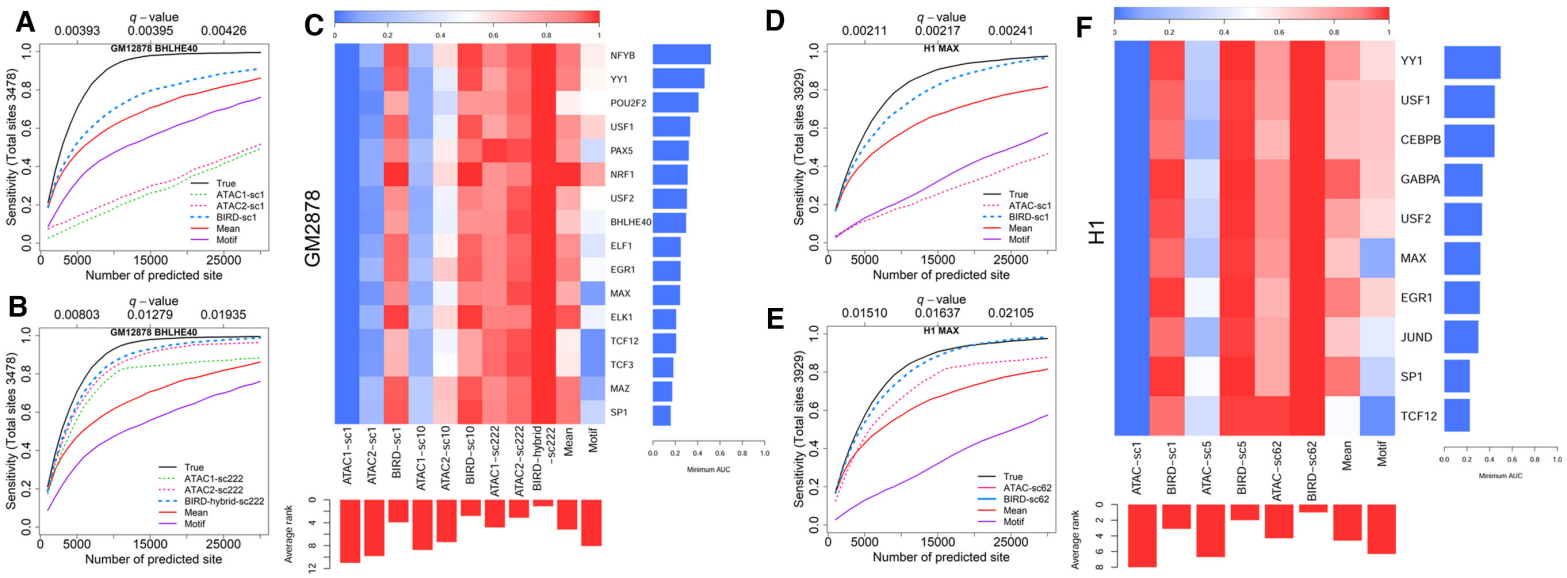
TFBS prediction using single-cell RNA-seq data. Predictions are made using true DNase-seq (‘True’), mean DH profile of training samples (‘Mean’), motif mapping score (‘Motif’), BIRD, and scATAC-seq (‘ATAC’) based on a single cell or pooling different number of cells. ‘ATAC1’ and ‘ATAC2’ correspond to two different scATAC-seq datasets. (**A**, **B**) Sensitivity-rank curve for predicting BHLHE40 binding sites in GM12878 using 1 cell and 222 cells respectively. (**C**) Scaled area under the curve (AUC) for different methods that predict TFBS in GM12878. Each row is a TF, and each column is a method. For each TF, different methods are ranked based on the AUC value, and the worst AUC value of all methods is shown on the right using a blue bar. The average rank of each method across all TFs is shown on the bottom using a red bar. Smaller rank means better performance. (**D**, **E**) Sensitivity-rank curve for predicting MAX binding sites in H1 using 1 cell and 62 cells respectively. (**F**) Scaled AUC for different methods that predict TFBS in H1. The *q*-values shown in (A)-(B) are calculated based on BIRD-sc1 and BIRD-hybrid-sc222 predictions in GM12878 respectively. *q*-values shown in (D) and (E) are calculated based on BIRD-sc1 and BIRD-sc62 predictions in H1 respectively.

To test whether predictions from scRNA-seq can predict TFBSs in a similar fashion as small-cell-number RNA-seq, we analyzed 16 TFs in GM12878 and 10 TFs in H1 (Figure 6). Once again, BIRD and BIRD-hybrid performed better than pooled scATAC-seq. For predicting TFBSs in GM12878, when using 1 cell or 10 cells, BIRD prediction outperformed ATAC1 and ATAC2 in all 16 TFs, and it outperformed mean DH in 15 of 16 TFs. Using 222 cells, BIRD-hybrid also outperformed ATAC1, ATAC2 in all 16 TFs, and it outperformed mean DH in 15 tested TFs (Figure 6C). For predicting TFBSs in H1, when using 1 cell, 5 cells, or 62 cells, BIRD prediction outperformed ATAC and Mean in all 10 tested TFs (Figure 6F).

**Figure 7.**
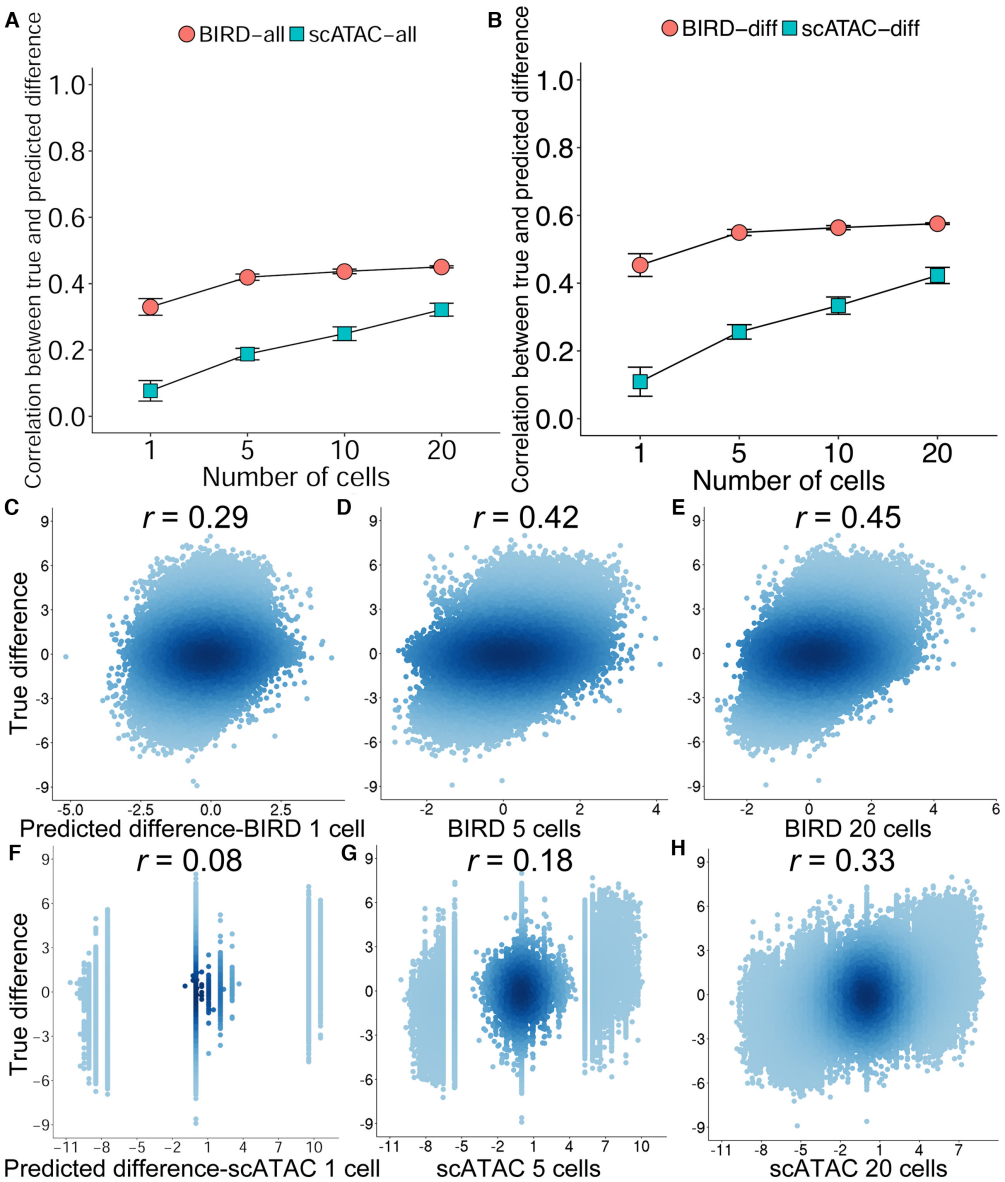
Predicting differential chromatin accessibility using single-cell RNA-seq data. (**A**) Pearson’s correlation between the true bulk GM12878-H1 differential DNase-seq signals and the BIRD-predicted (‘BIRD-all’) or scATAC-seq-measured (‘scATAC-all’) differential signals across all DHSs by using different number of cells. (**B**) Pearson’s correlation between the true and predicted differential signals at differential DHSs (‘BIRD-diff’ versus ‘scATAC-diff’). In (A) and (B), the average correlation and standard deviation from 10 independent random samplings of cells are shown for each method. (**C**–**E**) Scatterplots comparing the true bulk differential signals with the BIRD-predicted differential signals based on 1, 5 and 20 cells. (**F**–**H**) Scatterplots comparing the true bulk differential signals with the scATAC-seq-measured differential signals based on 1, 5 and 20 cells. *r*: Pearson’s correlation.

We then asked whether scRNA-seq can be used to predict differential chromatin accessibility between different cell types. In order to compare with scATAC-seq, this analysis requires cell types for which scRNA-seq, scATAC-seq, and gold standard (e.g. bulk DNase-seq) are all available. Few cell types currently have all these data types. Since bulk DNase-seq, scRNA-seq, and scATAC-seq were all available for GM12878 and H1, we conducted analysis using these two cell types. We pooled scRNA-seq data from *k* = 1, 5, 10 and 20 cells for each cell type and then predicted differential chromatin accessibility between GM12878 and H1 using BIRD. For comparison, we computed differential chromatin accessibility by pooling the same number of scATAC-seq cells from (10) (i.e. ATAC2). Differential signals between GM12878 and H1 computed using their bulk DNase-seq data were used as the gold standard. Figure 7A, C–H shows the correlation between the gold standard bulk differential signal and the BIRD-predicted or scATAC-seq derived differential signals across all loci. Using one single cell, BIRD is substantially better than scATAC-seq in predicting bulk differential signal (Figure 7C, F). As the cell number increased, scATAC-seq signals remained highly discrete for small cell number, whereas BIRD-predicted differential signals were continuous and showed better correlation with the bulk differential signal (Figure 7C–H). When the evaluation was focused on differential loci instead of all loci, both BIRD and scATAC-seq showed better performance, and BIRD still outperformed scATAC-seq (Figure 7B, Materials and Methods). With 20 cells from each cell type, the Pearson’s correlation between BIRD-predicted and true bulk differential signals were 0.45 and 0.58 for all loci and differential loci respectively, as compared to 0.32 and 0.42 between scATAC-seq and true bulk signals (Figure 7A and B). The correlation by BIRD using 20 cells (0.45 and 0.58) was close to the cross-sample correlation observed in bulk RNA-seq-based predictions (Figure 2E, mean *r*_C_ = 0.51). Importantly, it was better than the performance of the state-of-the-art technology scATAC-seq when the cell number was held the same, consistent with our analyses in Figures 5, 6 and Supplementary Figure S6. This analysis indicates that BIRD is capable of predicting cell type differences and hence can be used to study heterogeneity of a sample consisting of multiple cell types.

In order to test whether BIRD is robust to batch effects in the scRNA-seq data, we obtained new GM12878 and H1 scRNA-seq datasets from different labs (35). Cells in these new datasets were obtained using Fluidigm C1 microfluidics chips (Supplementary Table S1). BIRD applied to scRNA-seq data generated by different labs for the same cell type produced similar prediction accuracy, and the difference between labs was much smaller than the difference between BIRD and scATAC-seq (Supplementary Methods andSupplementary Figure S7). This indicates that BIRD is relatively robust to batch effects.

### Predicting chromatin accessibility using 10x genomics single-cell RNA-seq data

In the previous section, the scRNA-seq data were generated using single-cell technologies with relatively low throughput in terms of cell number. Recently, the Chromium Single Cell Gene Expression Solution provided by the 10x Genomics has enabled efficient scRNA-seq analysis for 10^3^–10^4^ cells (36), although the data for each cell becomes sparser (Supplementary Table S1: the average number of genes detected per cell = ∼2000 for 10x Genomics versus 10 000–20 000 for Fluidigm and micropipette datasets). During the revision of this article, 10x Genomics scRNA-seq data for human bone marrows became available in the Human Cell Atlas (HCA) (18). Human bone marrow contains hematopoietic stem cells (HSC) which develop into multipotent progenitor cells (MPP). MPP generates lymphoid-primed multipotent progenitor cells (LMPP) and common myeloid progenitor cells (CMP). LMPP will evolve to common lymphoid progenitor cells (CLP). CMP will differentiate to granulocyte-macrophage progenitor cells (GMP) and megakaryocyte-erythroid progenitor cells (MEP). GMP will further differentiate to monocyte cells and granulocyte cells. MEP will further differentiate to erythroid cells and megakaryocyte cells. CLP will further differentiate to T cells, B cells and natural killer cells (Supplementary Figure S8A). Thus, the bone marrow represents a heterogeneous cell population consisting of multiple cell types at different stages of hematopoiesis. In parallel to the HCA scRNA-seq data, a separate lab has used fluorescence-activated cell sorting (FACS) to purify multiple cell types in hematopoiesis and has generated bulk and single-cell ATAC-seq (Fluidigm C1) data for these sorted cell types (31,32). These data made it possible to evaluate BIRD on 10x Genomics scRNA-seq data and compare it with scATAC-seq. At the same time, ENCODE has released a larger set of 167 samples with both DNase-seq and RNA-seq data, and we have updated BIRD by retraining the model using these latest training data. Using these newly available single-cell data and the latest BIRD, we evaluated the ability of BIRD to make predictions using 10x Genomics scRNA-seq data.

Bone marrow scRNA-seq data from two different donors (BM1: 5915 cells; BM6: 5869 cells) were downloaded from HCA. Data from each donor represent a heterogeneous cell population, and the cell type for each individual cell is *a priori* unknown. These two donors were profiled by scRNA-seq in two different batches (1 donor per batch). Since the 10x Genomics scRNA-seq data are highly sparse, we first applied a recently developed imputation method scVI (25) to each donor to reconstruct gene expression values for single cells. BIRD was then applied to the scVI-reconstructed expression values to make predictions.

For evaluation, one needs to know the cell type for each cell. However, this information is unavailable for the bone marrow scRNA-seq data since the cells are unsorted. To address this, we first clustered cells using scRNA-seq (Supplementary Figure S8B and C). Next, we obtained bulk RNA-seq data from 13 FACS isolated pure cell types in hematopoiesis generated by (31). Using these bulk data, we computationally annotated the cell type for each cell cluster (Supplementary Methods). After intersecting scRNA-seq, bulk RNA-seq, scATAC-seq, and bulk ATAC-seq data, we obtained 7 cell clusters that simultaneously satisfied the three conditions below: (i) the cluster was unambiguously annotated by bulk RNA-seq and consistently annotated with the same cell type in both donors, (ii) the annotated cell type had both scRNA-seq and bulk and single-cell ATAC-seq data to allow evaluation and (iii) the annotated cell type was not included in the training data (Supplementary Methods). These clusters were used for evaluation. The other cell clusters that did not satisfy one or more conditions above were not included in our evaluation. The seven cell clusters that can be evaluated correspond to three different cell types: CMP, GMP and MEP. Note that multiple clusters can be annotated with the same cell type. Thus, we consolidated these clusters into three cell types by merging clusters annotated as the same cell type (Supplementary Figure S8D and E). Using these three cell types, we compared BIRD predictions with scATAC-seq, and bulk ATAC-seq was used as the gold standard. As before, to evaluate predictions for a cell type, we pooled different numbers of cells within the cell type and then applied BIRD to the pooled sample. BIRD and scATAC-seq were compared by holding the cell number the same.

**Figure 8.**
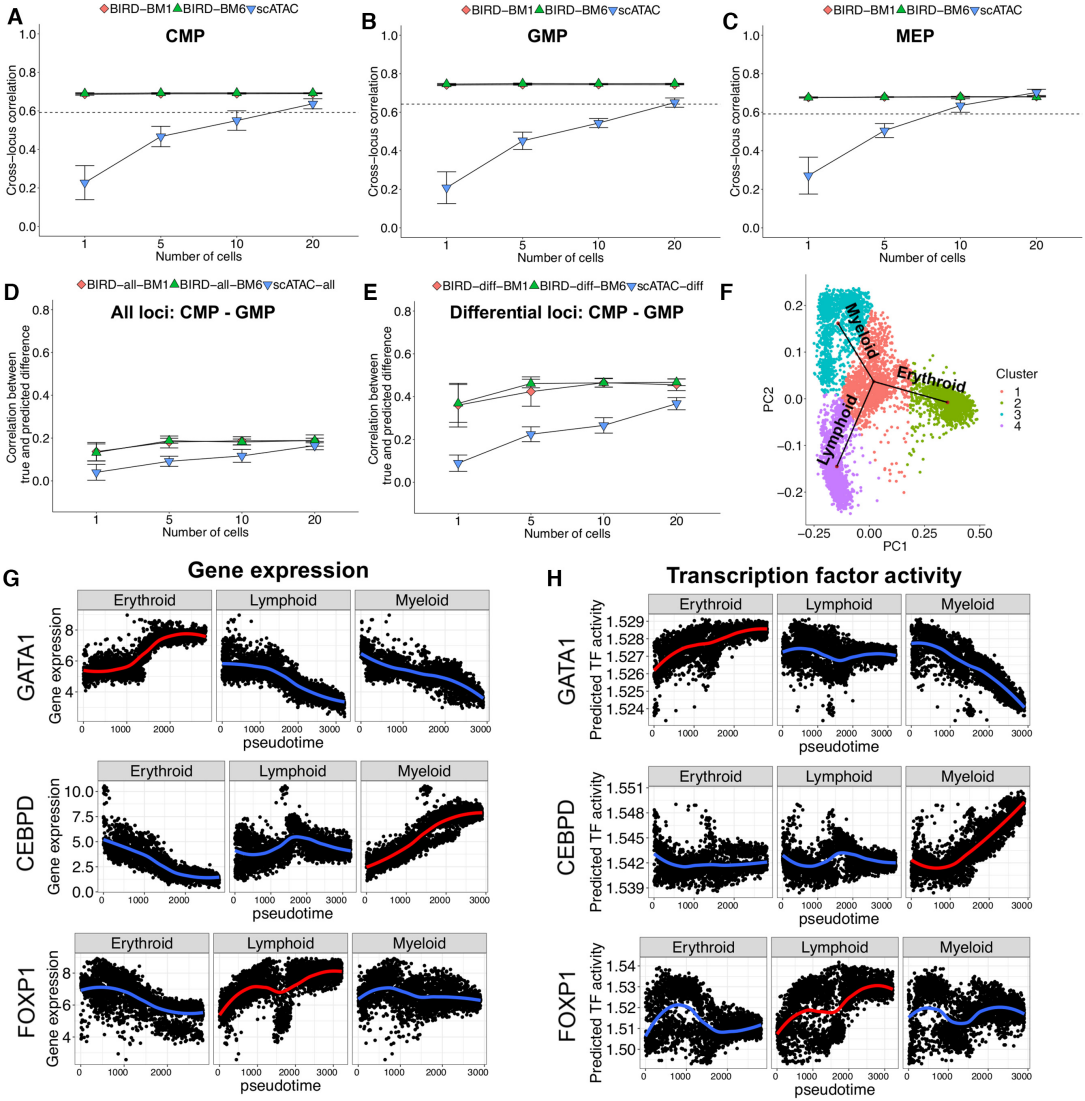
Predicting chromatin accessibility using 10x Genomics bone marrow scRNA-seq data. (**A**–**C**) Pearson’s correlation between the true bulk ATAC-seq signal and BIRD-predicted or scATAC-seq-measured chromatin accessibility across all DHSs by pooling different number of cells for (A) CMP, (B) GMP, and (**C**) MEP, respectively. BIRD prediction is based on scVI-imputed gene expression profiles from two donors, BM1 and BM6, respectively. (D)-(E) Pearson’s correlation between the true bulk CMP-GMP differential ATAC-seq signals and BIRD-predicted or scATAC-seq-measured differential signals by pooling different number of cells across (**D**) all DHSs and (**E**) differential DHSs. (**F**) The first two principal components of the scRNA-seq data in BM1 show three distinct lineages in TSCAN pseudotime analysis. (**G**) Gene expression of lineage-specific TFs, GATA1, CEBPD, and FOXP1 along pseudotime of the erythroid, lymphoid, and myeloid lineages in BM1. Each dot is a cell. Curves are the loess fit of the data. (**H**) The predicted TF binding activity of GATA1, CEBPD and FOXP1 along pseudotime of the three lineages in BM1. Pseudotime analysis for BM6 is in Supplementary Figure S11.

It should be pointed out that the evaluation here intrinsically favors scATAC-seq over BIRD because the bulk and single-cell ATAC-seq data were generated by the same lab using FACS sorted cells which represent relatively pure cell types. By contrast, the HCA bone marrow data were generated by a different lab using unsorted bone marrow samples from independent donors, and the cell type annotation was computationally inferred and may contain annotation noises (i.e. incorrectly annotated cells). Thus, one would expect that the bulk ATAC-seq correlates better with scATAC-seq than BIRD. Despite this intrinsic bias in favor of scATAC-seq, BIRD prediction in single cells and in pooled samples with a small number of cells outperformed scATAC-seq and showed stronger correlation with bulk ATAC-seq (Figure 8A–C). Similarly, BIRD also predicted differential chromatin accessibility between cell types more accurately than scATAC-seq when the cell number was small, as demonstrated by the correlation between the predicted and true values across all DHSs (Figure 8D and Supplementary Figure S9A and C) and across differential DHSs (Figure 8E and Supplementary Figure S9B, D). In these analyses, scATAC-seq only started to perform comparable to or better than BIRD when pooling 20 cells, despite the intrinsically biased comparison in favor of scATAC-seq.

Since the two donors were profiled by scRNA-seq in two different batches, their differences represent a combination of biological variability across human subjects and batch effects. Figure 8A–E and Supplementary Figure S9 show that the differences between BIRD predictions from the two donors were much smaller than the differences between BIRD and scATAC-seq. This again suggests that BIRD is relatively robust to batch effects.

For the sparse 10x Genomics scRNA-seq data, mitigating the sparsity by imputation before applying BIRD helped with improving the prediction accuracy. Supplementary Figure S10 shows that BIRD without applying scVI-imputation performed worse than applying BIRD after scVI-imputation. Unlike the 10x Genomics data, the scRNA-seq data obtained from Fluidigm C1 and micropipette in the previous section were less sparse. For those data, applying scVI-imputation did not significantly change the results (Supplementary Figure S7: imputation performed slightly better within each cell type, but performed slightly worse when predicting differences between two cell types).

BIRD may be combined with other methods developed for scRNA-seq data to analyze gene regulation. For example, in both donors, the bone marrow cells displayed three branches corresponding to three major lineages of hematopoietic differentiation, with undifferentiated and multipotent progenitor cells in the middle of the star topology and differentiated cells on the branch ends (Figure 8F: BM1, Supplementary Figure S11A: BM6). Using pseudotime analysis by TSCAN (37), we constructed cells’ pseudo-temporal trajectories along these three major branches. GATA1, an erythroid lineage-specific transcription factor, showed increased gene expression in the erythroid lineage as cells differentiate. This increased expression was erythroid lineage-specific and was not observed in the lymphoid or myeloid lineage (Figure 8G and Supplementary Figure S11B). Using BIRD, we inferred chromatin accessibility in each cell for DHSs that contained GATA1 motif. In each cell, the GATA1 motif binding activity was then inferred as the mean predicted chromatin accessibility across all GATA1-motif-containing DHSs. Consistent with GATA1 gene expression, the BIRD predicted GATA1 motif binding activity increased in the erythroid lineage but did not increase in the lymphoid or myeloid lineage (Figure 8H and Supplementary Figure S11C). Similarly, CEBPD, a myeloid lineage-specific regulator, and FOXP1, a lymphoid-specific regulator, also demonstrated lineage-specific increase in gene expression and BIRD-predicted motif binding activity in the myeloid lineage and lymphoid lineage, respectively (Figure 8G, H and Supplementary Figure S11B, C). Thus, the BIRD-predicted motif binding activities were consistent with the known biology (38,39) and the lineage-specific TF expression. This further illustrates how BIRD may be applied to scRNA-seq to infer chromatin dynamics.

## DISCUSSION

To summarize, our analyses show that predicting chromatin accessibility using RNA-seq can provide a new approach for regulome mapping in bulk, small-cell-number, and single-cell samples. We compared multiple state-of-the-art technologies for mapping regulome in low-input and single-cell samples including ATAC-seq, scATAC-seq, MOWChIP-seq and BIRD. Our results indicate that for analyzing low-input and single-cell samples, BIRD based on RNA-seq can offer competitive performance compared to ATAC-seq and scATAC-seq. In particular, using 10 folds fewer cells, BIRD based on small-cell-number RNA-seq reached the same accuracy as ATAC-seq for predicting bulk chromatin accessibility. BIRD based on fewer cells offered competitive or better performance compared to MOWChIP-seq using more cells. Moreover, when making predictions in single cells or pooling a small number of cells using scRNA-seq, BIRD was able to more accurately reconstruct bulk chromatin accessibility than using scATAC-seq by pooling the same number of cells.

Our analyses in GM12878 and H1 showed that BIRD prediction based on scRNA-seq from ≤10 cells can robustly recover chromatin accessibility at individual regulatory elements in a bulk sample. By contrast, averaging scATAC-seq from 20 to 50 cells predicted bulk chromatin accessibility worse than the trivial prediction based on the mean DH profile in both the GM12878 and H1 analyses. This highlights the limitation of the sparse data generated by the current scATAC-seq technology and the needs for further improving it (Figure 1). Compared with scATAC-seq, scRNA-seq data are less discrete as each gene can have more than two copies of transcripts in a cell. As such, it may provide additional information on regulome. In fact, in our study BIRD predictions based on scRNA-seq from a single cell were better than predictions based on the mean DH profile. Our analyses of the HCA bone marrow samples further demonstrate that BIRD can be applied to scRNA-seq data generated by the 10x Genomics platform and also provide competitive or better performance compared to scATAC-seq. The 10x Genomics platform can efficiently analyze a large number of cells, although the data is sparse. For such sparse data, using imputation to reconstruct gene expression before applying BIRD can help improve prediction.

In real applications, RNA-seq samples with small cell numbers may arise from cell sorting (e.g. FACS) which retrieves a subset of cells of the same cell type from a heterogeneous bulk sample. In this case, BIRD applied to the sorted cells will predict the chromatin accessibility of the sorted cell type. Small-cell-number RNA-seq samples may also arise when analyzing precious clinical samples or embryonic tissues. In those situations, each sample may contain multiple cell types and the number of available cells is limited even without any cell sorting. BIRD applied to such a low-input bulk sample will predict its bulk chromatin accessibility (i.e. the pooled chromatin profile of multiple cell types). In order to predict chromatin landscape of pure cell types, one needs to either sort the cells or use scRNA-seq. If the majority of cells in a small-cell-number sample has the same cell type, then BIRD prediction may still capture the chromatin landscape of that cell type, although the prediction accuracy for that cell type will deteriorate as the proportion of cells from other cell types (i.e. noise) increases. In Supplementary Methods andSupplementary Figure S12, we evaluated how intrinsic heterogeneity of a sample may affect the prediction accuracy. By pooling different proportions of GM12878 and H1 cells, the analyses suggest that when the proportion of the dominant cell type in a heterogeneous sample is large, one may still obtain reasonably accurate predictions for the dominant cell type.

Single-cell RNA-seq allows one to computationally cluster cells in a heterogeneous sample. Each cell cluster represents a relatively homogeneous cell subpopulation. In this way, one could separate different cell subpopulations without cell sorting. BIRD can then be applied to cells within each cell cluster to predict chromatin landscape for each cell subpopulation, similar to our bone marrow example. Thus, when applying BIRD to scRNA-seq, the input cells are not required to be sorted experimentally. Instead, one may computationally sort cells and define cell types by clustering, and then infer cell-type-specific chromatin landscape computationally. Our bone marrow analysis also suggests that BIRD may be combined with other scRNA-seq analysis methods such as pseudotime analysis to study gene regulatory programs.

BIRD is a supervised learning approach. In order to apply BIRD to a new sample, BIRD needs to be trained using training samples from the same species. When applying BIRD to a new sample to make prediction, the training data are not required to have the same cell type as the new sample. For example, in our analyses, the test cell types indeed were not included in the training data. However, the similarity between the new sample and the training samples may influence the prediction accuracy. When cell types similar to the new sample exist in the training data, the prediction is more likely to be accurate. When a new RNA-seq sample represents a unique new cell type substantially different from all cell types in the training data (i.e. the new sample falls outside the boundary of the training space), the prediction accuracy may drop due to the instability of extrapolation. In order to provide a practical guidance on when the prediction may be reliable, BIRD provides a function that computes the distance between a new RNA-seq sample and the training RNA-seq samples (training-test distance). In Supplementary Methods andSupplementary Figure S13A, we show that as the training-test distance increases, the prediction accuracy decreases. Overall, the prediction accuracy is relatively high and stable when the distance is smaller than 0.5, and the accuracy starts to drop faster when the distance is between 0.5 and 0.8. Of note, the training-test distances for RNA-seq data used in our analyses were in the range of 0.6 to 0.8 (Supplementary Figure S13B). In real applications, we recommend users to experimentally validate a few predictions whenever possible and use the experimental data or other independent sources of information to more accurately assess the empirical prediction accuracy in their data, particularly when the distance becomes bigger than 0.5.

For users’ convenience, we have released the pre-trained BIRD models for human along with the training data in the BIRD GitHub website. We also plan to release pre-trained models for other model organisms as enough training data for them become available. Users can use these pre-trained models directly without the need to compile their own training dataset and train their own models. On the other hand, if the training-test distance indicates that adding new training cell types into the training data is necessary to improve prediction accuracy for a new sample, users can also use the model training functions provided by BIRD to retrain their own models using the training data compiled by themselves (see tutorials at the BIRD GitHub website).

Our study has important practical relevance to future data analyses. It shows that transcriptome-based regulome prediction can greatly increase the value of the current and future bulk, low-input and single-cell RNA-seq experiments. By adding a new component to the standard RNA-seq analysis pipeline, this approach allows one to use RNA-seq to study not only transcriptome but also regulome. This can greatly influence how to most effectively use the enormous amounts of existing and future RNA-seq data. This is particularly relevant because (i) bulk and single-cell RNA-seq samples far outnumber bulk and single-cell samples from other functional genomic profiling technologies and (ii) many RNA-seq experiments are conducted without accompanying regulome profiling experiments.

Our study also has important implications for future small-cell-number or single-cell experiment design. For example, when a sample contains only a very limited number of cells and when an investigator does not have access to mature multi-omic solutions to measure both transcriptome and regulome in the same cell, the investigator may have to decide how the sample should be wisely used. Should one use all cells for transcriptome profiling by RNA-seq or regulome mapping by ATAC-seq? In that situation, our results suggest that one may divide the samples into two parts, one for RNA-seq or scRNA-seq, and one for ATAC-seq or scATAC-seq. This strategy has two advantages. First, one can obtain information for two different data types instead of only one data type. Second, by spending some cells on RNA-seq, BIRD-hybrid allows one to combine the two data types to produce comparable or better regulome mapping than spending all cells on ATAC-seq. This study also shows that if one decides to use all cells for RNA-seq, one can still obtain information on regulome through prediction. Thus, it is also possible to analyze transcriptome and regulome simultaneously by measuring only transcriptome.

As a proof-of-concept, this study shows that predicting chromatin accessibility using bulk, low-input, and single-cell RNA-seq is feasible. An important future research direction is to develop better methods to improve prediction accuracy. For instance, improving the normalization between scRNA-seq and the training bulk RNA-seq data and better addressing technical biases in these technologies could potentially increase the prediction performance. Another important next step is to explore whether other functional genomic data types such as DNA methylation and long-range chromatin interaction can be predicted in a similar fashion.

## Supplementary Material

gkz716_Supplemental_Files
