# Supplementary material for: Global prediction of chromatin accessibility using small-cell-number and single-cell RNA-seq
Source: Nucleic Acids Res. 2019 Aug 20;47(19):e121. doi: 10.1093/nar/gkz716 (PMC6821224; doi:10.1093/nar/gkz716)
Supplement: gkz716_Supplemental_Files [file gkz716_supplemental_files.zip › Supplementary_materials.pdf]

SUPPLEMENTARY METHODS

DNase-seq data processing

For training the BIRD model, the aligned DNase-seq data (alignment based on hg19) from 70 samples (**Dataset S1**, representing 30 different cell types) were downloaded from the Roadmap Epigenomics project (1) (ftp://ftp.genboree.org/EpigenomeAtlas/Current-Release/experiment-sample/Chromatin_Accessibility/). The analyses in this study were focused on chromosomes 1 to X. Excluding chromosome Y, the genome was divided into 200 base pair (bp) non-overlapping bins. The number of reads mapped to each bin was counted for each DNase-seq sample. To adjust for different sequencing depths, bin read counts for each sample i were first divided by the sample's total read count N_i and then scaled by multiplying a constant N ($N = \min_i\{N_i\}$ = 12,422,306, which is the minimum sample read count of all samples). The normalized read counts were then log2 transformed after adding a pseudocount of 1. The normalized and log2-transformed read counts were used to represent DH levels of genomic bins.

For testing prediction performance, DNase-seq data for GM12878 and H1 were downloaded from the ENCODE project (2) . The data were aligned to human genome hg19 using bowtie (3) (http://hgdownload.cse.ucsc.edu/goldenPath/hg19/encodeDCC/wgEncodeUwDnase). The aligned reads were processed in the same way as the Epigenome Roadmap data to derive DH levels. Note that the ENCODE data contained replicate samples for each cell type. The normalized read counts from replicate samples were first averaged to characterize the DH level for each bin in each cell type. The DH level was then log2 transformed after adding a pseudocount of 1.

Genomic loci filtering

Since most genomic loci are noise rather than regulatory elements, we filtered genomic loci to exclude those without strong DH signal in any training DNase-seq sample. The filtering was done in three steps. First, genomic bins with normalized read count $\leqslant$8 in all samples were excluded. Second, bins with normalized read count larger than 10,000 in $\geqslant$1 sample were considered abnormal and therefore also excluded. Third, a signal-to-noise ratio (SNR) was computed for each bin in each sample, and bins with SNR $\leqslant$ 2 in all samples were considered as noise and filtered out. In order to compute SNR of a genomic bin in a sample, we first collected 500 bins in the neighborhood of the bin in question. The average DH level of these bins was computed and then log2 transformed after adding a pseudocount of 1 to serve as the background. The $\log_2$(SNR) was defined as the difference between the normalized and log2 transformed DH level of the bin in question and the background. SNR $\leqslant$ 2 is equivalent to $\log_2$(SNR) $\leqslant$ 1.

After filtering, 1,136,465 genomic bins (called DNase I hypersensitive sites, or DHSs, hereinafter) with unambiguous DNase-seq signal in at least one sample were identified. Most analyses in this study were performed on these genomic loci, except for the leave-one-out cross-validation analysis in **Figure 2d-e** and analyses involving H1 in **Figure 5d**, **Figure 6d-f** and **Figure 7**. Analyses involving the 10x Genomics bone marrow data from the HCA will be described in separate sections below.

Bulk RNA-seq data processing

The aligned RNA-seq data (alignment based on hg19) for the same 70 Epigenome Roadmap samples were downloaded from ftp://ftp.genboree.org/EpigenomeAtlas/Current-Release/experiment-sample/mRNA-Seq/. Cufflinks (4) was used to compute the expression values (i.e., FPKM: fragments per kilobase of transcript per million mapped fragments) using gene annotations in GENCODE (5) (Release 19 (GRCh37.p13)). 37,335 transcripts (called "genes" hereinafter for simplicity) with FPKM > 1 in at least one sample were identified. These FPKM values were log2 transformed after adding a pseudocount of 1 and then quantile normalized across samples. After normalization, the quantiles of the Epigenome Roadmap training data were stored for future use. When new RNA-seq samples need to be analyzed, they are quantile normalized against these stored quantiles.

For evaluating prediction performance, we also downloaded the following data from GEO: 1) GM12878 bulk RNA-seq data (GSM958728); 2) GM12878 RNA-seq data from small-cell-number samples with 30 cells (GSM1087858, GSM1087859) and 100 cells (GSM1087856, GSM1087857). For evaluating impacts of batch effects, we further obtained bulk RNA-seq data from three different labs for GM12878 (GSM1663002, GSM3612677, GSM958728) and H1 (GSM2680565, GSM1494448, GSM915328). For these samples, reads were mapped to human genome hg19 using Tophat (6). Gene expression values were then computed using Cufflinks in the same way as how we processed the Epigenome Roadmap RNA-seq data. Finally, the gene expression values were quantile normalized with the Epigenome Roadmap RNA-seq data using the stored quantiles.

BIRD model

Details of BIRD are described in Zhou *et al.* (7). Below we review this method for readers' convenience.

Problem formulation:

For a biological sample, let Y_l be the DH level of genomic locus l (=$1, \dots, L$), and let X_g be the expression level of gene g (=$1, \dots, G$). The genome-wide DH profile and gene expression profile are represented by two vectors $\boldsymbol{Y} = (Y_1, \dots, Y_L)^T$ and $\boldsymbol{X} = (X_1, \dots, X_G)^T$ respectively. Here, the superscript T indicates matrix or vector transpose. Both the DH and gene expression profiles are assumed to be normalized and at log2 scale. Our goal is to use $\boldsymbol{X}$ to predict $\boldsymbol{Y}$. BIRD formulates it as a problem of building a regression $Y_l = f_l(\boldsymbol{X}) + \epsilon_l$ for each genomic locus. Here ϵ_l represents random noise, and $f_l(.)$ is the function that describes the systematic relationship between the DH level of locus l (i.e., Y_l) and the gene expression profile (i.e., $\boldsymbol{X}$).

The function $f_l(\boldsymbol{X})$ is unknown. BIRD trains it using $\boldsymbol{X}$ and $\boldsymbol{Y}$ observed from a number of different samples. The training data are organized into two matrices: a gene expression matrix $\mathbb{X} = (x_{gc})_{G \times C}$ and a DH matrix $\mathbb{Y} = (y_{lc})_{L \times C}$. Rows in these matrices are genes and genomic loci respectively. Columns in these matrices are samples. C is the number of training samples. Each column of $\mathbb{X}$ and $\mathbb{Y}$ is a realization of the random vector $\boldsymbol{X}$ and $\boldsymbol{Y}$ in a specific sample.

Let a_l^y and s_l^y be the mean and standard deviation (SD) of the DH signals at locus l (i.e., row l of $\mathbb{Y}$), and let a_g^x and s_g^x be the mean and SD of the gene expression for gene g (i.e., row g of $\mathbb{X}$). Before training, each row of $\mathbb{X}$ in the training data is standardized to have zero mean and unit SD using a_g^x and s_g^x. Similarly, each row of $\mathbb{Y}$ in the training data is standardized to have zero mean and unit SD using a_l^y and s_l^y. The standardized matrices $\tilde{\mathbb{X}}$ and $\tilde{\mathbb{Y}}$ are then used to construct prediction models.

Once the prediction models are trained, they can be applied to new RNA-seq samples to make predictions. Let $\boldsymbol{X}$ be the expression profile of the new sample after being normalized to the stored quantiles of the training data. The expression value of each gene X_g in the new sample is first standardized using $\tilde{X}_g = (X_g - a_g^x)/s_g^x$. Recall that a_g^x and s_g^x are the mean and SD of the gene expression for gene g in the training data, and they have been computed and stored when BIRD was trained. Next, the trained model is applied to the standardized gene expression profile to make predictions. The predicted DH value for each locus, $\tilde{Y}_l$, is transformed back using $\hat{Y}_l = s_l^y * \tilde{Y}_l + a_l^y$, where a_l^y and s_l^y are the mean and SD of the DH signals for locus l in the training data. The unstandardized $\hat{Y}_l$ gives the prediction for Y_l, the DH level of genomic locus l in the new sample.

Model training:

BIRD is constructed in three steps (**Fig. S1a-b**).

- Step 1 – Construct elementary model $\mathrm{BIRD}_{\tilde{\mathrm{X}},\mathrm{Y}}$: BIRD first groups correlated predictors into clusters. This is done by clustering rows of the standardized training data matrix $\tilde{\mathbb{X}}$ into K clusters using k-means clustering (8) (Euclidean distance is used as similarity measure). Based on the clustering result, the gene expression profile $\tilde{\boldsymbol{X}}$ of each sample is converted into a lower dimensional vector $\bar{\boldsymbol{X}} = (\bar{X}_1, \dots, \bar{X}_K)$, where $\bar{X}_k$ is the mean expression level of genes in cluster k. BIRD uses gene clusters' mean expression $\bar{\boldsymbol{X}}$ instead of the expression of individual genes $\tilde{\boldsymbol{X}}$ as predictors to build prediction models. Clustering reduces the dimension and co-linearity of the predictors, and it also makes predictors less sensitive to measurement noise (e.g., dropouts in scRNA-seq) of individual genes. After clustering, the $G \times C$ matrix $\tilde{\mathbb{X}}$ is converted into a $K \times C$ matrix $\bar{\mathbb{X}}$ ($G \approx 10^4$, $K \approx 10^2 \sim 10^3$). The predictor dimension is reduced, but it is still high compared to sample size. We further reduce the predictor dimension by using a fast variable screening procedure: for each DHS locus l, the Pearson's correlation between its DH signal (i.e., row l of $\tilde{\mathbb{Y}}$) and the expression of each gene cluster k (i.e., row k of $\bar{\mathbb{X}}$) across the training samples is computed, and the top N ($\approx 10^1$) clusters with the largest correlation coefficients are selected. Using the selected clusters $(\bar{X}_{l_1}, \dots, \bar{X}_{l_N})$ as predictors, a multiple linear regression $\tilde{Y}_l = \beta_{l0} + \beta_{l1}\bar{X}_{l_1} + \dots + \beta_{lN}\bar{X}_{l_N} + \epsilon_l$ is then fitted. Based on the fitted model, the standardized DH level of locus l in a new sample can be predicted by $\tilde{Y}_l = f_l(\tilde{\boldsymbol{X}}) = \beta_{l0} + \beta_{l1}\bar{X}_{l_1} + \dots + \beta_{lN}\bar{X}_{l_N}$. The procedure above has two tuning parameters *K* (the number of gene clusters) and *N* (the number of predictors). They are chosen based on building models for a random subset (1%) of the 1,136,465 studied loci using different values of *K* and *N* (*K*= 100, 200, 500, 1000, 1500, 2000, 2500, 3000, 3500, 4000, 4500; *N*=1, 2, 3, 4, 5, 6, 7, 8). A five-fold cross-validation is performed within training samples. The best combination of *K* and *N* that optimizes the cross-validation prediction accuracy is then selected. This

parameter combination will be used for all genomic loci. It is also used in the subsequent $\mathrm{BIRD}_{\bar{X},\bar{Y}}$ models. As an example, when all 70 samples were used to train the model, we obtained *K*=3000 and *N*=4.

- Step 2 – Construct cluster-level model $\mathrm{BIRD}_{\bar{X},\bar{Y}}$: We further group correlated responses (i.e., genomic loci with similar DH patterns) into clusters. This is done by clustering rows of the standardized matrix $\tilde{\mathbb{Y}}$ into H clusters using k-means clustering (Euclidean distance is used as similarity measure). Based on the clustering result, the DH profile $\tilde{\boldsymbol{Y}}$ of each sample can be converted into a lower dimensional vector $\bar{\boldsymbol{Y}} = (\bar{Y}_1, \dots, \bar{Y}_H)$, where $\bar{Y}_h$ is the mean DH level of DHSs in cluster h. Instead of predicting the DH level $\tilde{\boldsymbol{Y}}$ of individual loci, $\mathrm{BIRD}_{\bar{X},\bar{Y}}$ uses the cluster-level gene expression $\bar{\boldsymbol{X}}$ to predict cluster-level DH $\bar{\boldsymbol{Y}}$. The prediction models are constructed using linear regression in a way similar to how the regression models are constructed in $\mathrm{BIRD}_{\bar{X},Y}$. For $\mathrm{BIRD}_{\bar{X},\bar{Y}}$, H was set to 1000, 2000 and 5000 respectively. For each H, we repeated the model training procedure.

- Step 3 – Construct the final prediction model BIRD: $\mathrm{BIRD}_{\bar{X},Y}$ is a special case of $\mathrm{BIRD}_{\bar{X},\bar{Y}}$ when DHSs are not clustered (i.e., $H = L$). In $\mathrm{BIRD}_{\bar{X},\bar{Y}}$, one may use the predicted cluster mean as the predicted DH level of each individual locus within the cluster. This will also generate a prediction for each locus. This locus-level prediction may be biased, but it is usually associated with smaller variance because cluster mean reduces variability. In the final BIRD model, multiple $\mathrm{BIRD}_{\bar{X},\bar{Y}}$ models with different H values are combined through model averaging to achieve a better bias-variance tradeoff and improve the prediction accuracy. Consider making predictions for a sample. Let $\mathcal{H}$ be the set of H values used by $\mathrm{BIRD}_{\bar{X},\bar{Y}}$. $\mathcal{H} = \{1000, 2000, 5000, L\}$. For each DHS locus l, let $\hat{Y}_l^{(H)}$ denote the locus-level DH predicted by $\mathrm{BIRD}_{\bar{X},\bar{Y}}$ using cluster number H. $\hat{Y}_l^{(L)}$ is the locus-level DH predicted by $\mathrm{BIRD}_{\bar{X},Y}$. The final BIRD prediction for locus l is a weighted average: $\frac{\sum_{H \in \mathcal{H}} d_l^H \hat{Y}_l^{(H)}}{\sum_{H \in \mathcal{H}} d_l^H}$. Here d_l^H is the weight. For a given cluster number *H*, the weight d_l^H is determined using training data as follows. Let $\tilde{\boldsymbol{y}}_l = (\tilde{y}_{l1}, \dots, \tilde{y}_{lM})$ be the standardized locus-level DH for locus l observed in *M* training samples. Each locus l is associated with a cluster. Let $\tilde{\boldsymbol{y}}_l^{(H)} = (\tilde{y}_{l1}^{(H)}, \dots, \tilde{y}_{lM}^{(H)})$ represent the average of the standardized DH level of all loci within the cluster. d_l^H is the Pearson's correlation between the two vectors $\tilde{\boldsymbol{y}}_l^{(H)}$ and $\tilde{\boldsymbol{y}}_l$. Note that when $H = L$, $\mathrm{BIRD}_{\bar{X},\bar{Y}}$ reduces to $\mathrm{BIRD}_{\bar{X},Y}$, and therefore $\tilde{\boldsymbol{y}}_l^{(L)} = \tilde{\boldsymbol{y}}_l$ and $d_l^L = 1$. Consequently, the weight for $\mathrm{BIRD}_{\bar{X},Y}$ is 1.

Leave-one-out cross-validation

Leave-one-out cross-validation was used to evaluate BIRD prediction accuracy when bulk RNA-seq data were used as predictors. In each fold of the cross-validation, the 30 Epigenome Roadmap cell types (consisting of 70 samples) were partitioned into a training dataset with 29 cell types and a test dataset with 1 cell type. In other words, all samples from one cell type were used as test data, and all samples from the remaining 29 cell types were used as the training data. BIRD was then trained using all samples in the training dataset and applied to predict DH for all samples in the test dataset.

To ensure that the test data are not used in the construction of prediction models, the predictor and genomic loci filtering procedure was applied to each fold of cross-validation by using the training data only. For instance, we identified genes with FPKM > 1 in at least one RNA-seq sample in the training data as predictors. The identified predictors were a subset of the 37,335 genes described before (note: the 37,335 genes were identified using all 70 samples rather than using only the training samples). For different folds of cross-validation, a slightly different set of predictors was identified. Similarly, genomic loci (i.e. DHSs) to be predicted were selected by applying the previously described filtering protocol to the training data only: 1) normalized bin read count ⩾8 in at least one sample; 2) normalized bin read count < 10,000 in all samples; 3) SNR ⩾ 2 in at least one sample. Prediction models were constructed for the identified genomic loci. These loci varied from fold to fold, and they were also slightly different from the 1,136,465 genomic loci derived from all 70 samples. For each fold, the parameters of BIRD were selected based on the training dataset (i.e., samples from 29 cell types in each fold). Since the training data were different in each fold, the parameters also varied from fold to fold. For comparison, the mean DH profile (indicated as "Mean" in **Fig. 2d-e**) from the training dataset was also obtained and then used as the predicted DH profile for the test samples in each fold of the leave-one-out cross-validation.

After predictions were made for all samples, r_L and r_C were calculated between the true and predicted DH profiles. Conceptually, one can organize the predicted DH values into a matrix. Rows of the matrix correspond to genomic loci, and columns of the matrix correspond to samples. The matrix has missing values as not all genomic loci have prediction models in all samples. This is because genomic loci filtering was dependent on the training data. As a result, in each fold of cross-validation, prediction models were built for a slightly different set of genomic loci. To compute r_L and r_C, missing data points in the prediction matrix were excluded, and only data points with predicted DH values were used.

Cross-sample prediction accuracy stratified by different classes of loci

We investigated what factors may influence cross-sample prediction accuracy r_C and may be used to identify loci for which cross-sample variation can be accurately predicted (**Fig. 2f-g**). To this end, we conducted an analysis similar to Zhou *et al.* (7). We grouped DHSs according to the following factors based on their BIRD-predicted DH values (at log2 scale; same below).

1) Signal range (max-min spread): for each locus, the difference between its maximum and minimum DH values in all samples was calculated to characterize its signal range. According to signal range, loci were labeled as low spread (loci with max-min spread below its 1st quartile) and high spread (all remaining loci).

2) Signal variability (coefficient of variation, i.e., CV): for each locus, we calculated the CV of its DH values in all samples. CV is the ratio of the standard deviation to the mean of the DH values. According to signal variability, loci were labeled as low CV (loci with CV below its 1st quartile) and high CV (all remaining loci).

3) Cell-type-specificity (no. of active or inactive cell types): for each locus, the number of cell types in which the locus is active (i.e., DH level on log2 scale was larger than 2) was counted. According to cell-type-specificity,

loci were labeled as high specificity (loci active in ≤1 cell type or inactive in ≤1 cell type) and low specificity (all remaining loci).

Fig. 2f shows the distribution of r_C for loci in each category. This figure shows that loci with low max-min spread, low CV or high cell-type-specificity tend to have lower r_C.

One may use these factors to screen for loci with high r_C. To demonstrate, in **Fig. 2g**, we used BIRD-predicted DH values to compute max-min spread, CV and cell-type-specificity of each locus. We then filtered out loci with low max-min spread, low CV or high cell-type-specificity. **Fig. 2g** shows the r_C distribution for the filtered loci, the retained loci, and all loci, respectively. For the retained loci, the mean r_C increased to 0.65. In contrast, the mean r_C was 0.51 for all loci, and 0.30 for loci that were filtered out.

Comparing BIRD with ChromImpute

We compared BIRD predictions with predictions made by ChromImpute in (9). In order to conduct this comparison, we collected 17 cell types (**Dataset S1**) for which predictions have been made by both methods.

For ChromImpute, the predicted DH signals were based on -log10 p-values ("pval-signal"). We downloaded both the predicted (i.e., imputed) DH signal and the true DH signal (i.e., -log10 p-value of DNase-seq counts relative to expected background calculated by MACS (10)) from the Roadmap Epigenomics compendium (1) and converted the data to 200 bp resolution using the "Convert" program provided by ChromImpute. For BIRD, the predicted signals were based on log-transformed normalized read counts ("read-signal"). The predictions were made by applying the leave-one-cell-type-out cross-validation procedure described in the previous section. The mean DH profile of the training cell types in each fold of the leave-one-out process was obtained as a control (indicated by "Mean"). For each fold of the leave-one-cell-type-out process, we used the protocol described above to filter DHSs. We then compared DH signals predicted by BIRD, ChromImpute and Mean at these genomic sites.

When a cell type has multiple samples, we averaged the predicted DH across replicate samples to serve as the BIRD prediction to be compared with ChromImpute. Similarly, multiple samples from the same cell type were also averaged to derive the true read-signal.

Because ChromImpute-reported signals were based on -log10 p-value ("pval-signal") and BIRD-reported signals were based on log-transformed normalized read counts ("read-signal"), they were not on the same scale. Therefore, we compared BIRD with ChromImpute using Spearman's rank correlation and two different gold standards. First, we used the true read-signal (i.e., log normalized read count from the real DNase-seq data) as the gold standard. For each method, we calculated the Spearman's rank correlation between the gold standard and predicted DH signals across genomic loci for each sample (i.e., r_L, **Fig. S2a**) and across samples for each genomic locus (i.e., r_C, **Fig. S2b**). Second, we used the true pval-signal as the gold standard and calculated the Spearman's rank correlation between the gold standard and predicted signals across genomic loci (i.e., r_L, **Fig. S2c**) and across samples (i.e., r_C, **Fig. S2d**). In all analyses, BIRD and ChromImpute substantially outperformed predictions based on the mean DH profile, and BIRD prediction accuracy was very close to the accuracy of ChromImpute. Here, BIRD predictions were based on RNA-seq alone, whereas ChromImpute predictions were based on multiple functional

genomic data types including ChIP-seq data of multiple histone modifications. Note that compared to RNA-seq, histone modifications are marks of chromatin states, and hence they more directly correlate with DNase I hypersensitivity (11, 12) .

Bulk ATAC-seq data processing

ATAC-seq data for GM12878 with 50,000 and 500 cells were obtained from GEO (GSE47753). The paired-end reads were aligned to human genome hg19 using bowtie with parameters (-X 2000 -m 1) which specify that paired reads (a pair of reads was referred to as a fragment) with insertion up to 2,000 base pair (bp) were allowed to align and only uniquely aligned fragments were retained. Then, PCR duplicates (i.e. fragments that aligned to exactly the same genomic location) were determined using Picard (http://broadinstitute.github.io/picard/) where only one fragment was kept and the others were removed. Next, we measured the bin-level fragment coverage by counting how many fragments covered each 200bp genomic bin. Similar to the DNase-seq data, bin-level fragment coverage for each sample was first divided by the sample's whole-genome fragment coverage (i.e., sum of bin-level fragment coverage across the genome) and then scaled by a constant *N* (=12,422,306, to be consistent with the DNase-seq data). Finally, the normalized bin fragment coverage from different replicate samples were averaged and log2 transformed after adding a pseudocount of 1.

Transcription factor binding site (TFBS) prediction

BIRD models trained using the 70 Epigenome Roadmap samples were applied to predict binding sites for 16 TFs in GM12878 cells. Models trained using 65 Epigenome Roadmap samples after excluding H1 were used to predict binding sites for 10 TFs in H1 cells. For each TF, DNA motif obtained from TRANSFAC (13) and JASPAR (14) (**Dataset S1**) was computationally mapped to the human genome using CisGenome (15) (using default likelihood ratio ≥ 500 cutoff). DHSs (i.e., the 1,136,465 genomic bins) that overlapped with motif sites were retained for subsequent analyses. These motif-containing DHSs were ranked in decreasing order based on the predicted DH level to serve as the predicted TFBSs. As a comparison, DHSs were also ranked based on three other methods: the true DH level at each DHS from the corresponding DNase-seq data ("True"), the DH level predicted based on the mean DH profile of the training samples ("Mean"), and the highest motif mapping score of each DHS (i.e., the maximal CisGenome-reported log likelihood ratio score of all motif sites mapped in a DHS) ("Motif").

To evaluate the prediction performance of different methods, the transcription factor ChIP-seq uniform peaks data for the 16 TFs in GM12878 and 10 TFs in H1 were downloaded from the ENCODE project to serve as the truth (http://hgdownload.cse.ucsc.edu/goldenPath/hg19/encodeDCC/wgEncodeAwgTfbsUniform/). ChIP-seq peaks overlapped with motif sites were used as the gold standard. The percentage of these gold standard peaks that were recovered by the top ranked predicted TFBSs was computed to measure the sensitivity of each prediction method. Different methods were compared by plotting the sensitivity as a function of the number of predicted TFBSs (**Fig. 4a-b**, **Fig. 6a-b,d-e**).

To evaluate statistical significance of the predicted TFBSs, the same BIRD models were applied to a set of randomly sampled genomic bins (n= 984,213, sampled from non-repeat genomic regions) to make predictions.

Using the predicted DH values in the random genomic loci as the null distribution, a *p*-value was computed for each studied DHS to evaluate the significance of its predicted DH level (*p*-value of the predicted DH level at a DHS = [no. of random loci with equal or larger predicted DH levels] / [the total no. of random loci]). To adjust for multiple testing, the *p*-values were converted to *q*-values based on the previously described method (16). *q*-values for BIRD predictions were labeled on top of each sensitivity-rank plot (e.g., **Fig. 4a-b**, **Fig. 6a-b,d-e**).

To generate **Figure 4c** and **Figure 6c,f** that compare different TFBS prediction methods using small-cell-number or single-cell RNA-seq data, we computed the area under the curve (AUC) for each method using the sensitivity rank curves. The AUC of each method was then scaled by (i.e., divided by) the AUC obtained using the true DNase-seq data. To show a clear comparison of different methods for predicting binding sites of each TF, colors in the heatmap (**Fig. 4c** and **Fig. 6c,f**) reflect the transformed AUC values. For instance, values within each row were transformed to the range between 0 and 1 by: [AUC value – min(value)]/[max(value) – min(value)], here min(value) and max(value) represent the minimum and maximum AUC value within each row. Note that within each TF, the minimum AUC was transformed to 0 and the maximum AUC was transformed to 1. As a reference, the untransformed minimum AUC value from all methods was shown for each TF using a blue bar beside the TF name in **Figure 4c** and **Figure 6c,f**.

To measure the overall prediction performance of each method, we calculated the average rank score (shown using red bars under the name of each method in **Fig. 4c** and **Fig. 6c,f**) across all test TFs. First, for each TF, different methods were ranked according to their AUC values. For instance, in **Figure 4c**, the best performing method has rank 1 and the worst performing method has rank 10. Then, we calculated the average rank across all test TFs for each method. Smaller average rank indicates better overall prediction performance.

Histone modification ChIP-seq and MOWChIP-seq data processing

H3K27ac and H3K4me3 MOWChIP-seq data for GM12878 with 100 and 600 cells were obtained from GEO (GSE65516: GSM1666202, GSM1666203, GSM1666204, GSM1666205, GSM1666206, GSM1666207, GSM1666208, GSM1666209). For both histone marks, the processed signal files provided by the MOWChIP-seq authors were downloaded from GEO. These signal files contained normalized read counts for the whole genome divided by 100 bp bins (17). The data were first converted to 200 bp bin resolution by merging the adjacent two 100 bp bins (adding the normalized read counts of the two 100 bp bins).

Due to nucleosome displacement, the spatial distribution of histone modification signal surrounding each regulatory element (e.g., transcription factor binding site) may differ from the peak of the DNase-seq and ATAC-seq signal (18). Therefore, we first explored different ways to summarize the histone modification signal in order to maximize its correlation with the bulk DNase-seq data. To do so, we considered a *W*-bp long window centered at each genomic locus (200bp bin). The normalized read counts of all 200bp bins covered by the window were averaged to serve as the summary of the histone modification signal at the locus. The summarized signals from replicate samples were averaged and log2 transformed after adding a pseudocount of 1. We then tested different window sizes (*W*=200, 600, 1000, 1400, 1800, 2200, 2600 bp) to find the optimal *W* that maximizes the correlation between the summarized histone modification signal and the bulk DNase-seq signal (i.e., DH level at 200-bp

resolution as described before) across all genomic loci (**Fig. S5a**). For H3K27ac with 100 and 600 cells, the optimal *W* was 2200. For H3K4me3 with 100 cells, the optimal *W* was 2200. For H3K4me3 with 600 cells, the optimal *W* was 1800. The summarized MOWChIP-seq signals using these optimal *W* were then compared with BIRD and ATAC-seq in **Figures 3, S4** and **S5d.**

H3K27ac and H3K4me3 ChIP-seq data for bulk GM12878 samples were obtained from ENCODE (http://hgdownload.cse.ucsc.edu/goldenPath/hg19/encodeDCC/wgEncodeBroadHistone/). For each 200bp genomic bin, reads from the bulk ChIP and input control samples were counted. Bin read counts in each sample were normalized by the sample's total read count and then scaled by multiplying 1,000,000. Signals were then calculated as the difference between the ChIP and input samples. Similar to MOWChIP-seq, we used the average signal of a *W*-bp long window (*W*=200, 600, 1000, 1400, 1800, 2200, 2600 bp) centered at each genomic locus to represent its summarized histone modification signal. The summarized signals from replicate samples were then averaged and log2 transformed after adding a pseudocount of 1. The optimal *W* was determined by maximizing the correlation with the bulk DNase-seq signal. For bulk H3K27ac and H3K4me3, the optimal *W* was 1000 and 1400 respectively (**Fig. S5c**). The summarized ChIP-seq signals using these optimal *W* were then used for generating **Figure S5d.**

For TFBS prediction, we also first optimized the window size *W* for each MOWChIP-seq dataset. For each histone mark and cell number, we obtained the scaled AUC (scaling is done by dividing the AUC of using true DNase-seq data to predict TFBSs) for all test TFs using different window sizes. For each TF, different window sizes were then ranked based on the scaled AUC. The average rank of each window size *W* across all test TFs was computed (**Fig. S5b**). *W* with the best average rank was identified. For H3K27ac MOWChIP-seq with 100 and 600 cells, the optimal *W* was 1800. For H3K4me3 MOWChIP-seq with 100 cells, the optimal *W* was 2200. For H3K4me3 MOWChIP-seq with 600 cells, the optimal *W* was 1800. The summarized MOWChIP-seq signals using these optimal *W* were then compared with BIRD and ATAC-seq in **Figure 4.** We note that since TFBSs of different TFs may be associated with different histone modification signatures (e.g., different types of histone modifications), a better way to predict TFBS might be to use multiple types of histone modification data jointly and develop a prediction model specific for each TF. However, that would make the TFBS prediction more difficult to apply in practice because one would need to collect more MOWChIP-seq data and have knowledge on the histone modification signature for each TF. For this reason, our analyses here were primarily focused on evaluating the performance of using one data type (i.e., H3K27ac, H3K4me3, ATAC-seq, or RNA-seq) and a common prediction procedure for all TFs. This makes the comparison among MOWChIP-seq, ATAC-seq (ATAC-b500) and BIRD (BIRD-b30) relatively fair in the sense that different TFBS prediction methods have similar level of complexity in terms of data collection and computational analysis.

Predicting chromatin accessibility based on 10x Genomics single-cell RNA-seq data

During the revision of this article, we obtained the 10x Genomics bone marrow scRNA-seq data from the Human Cell Atlas (HCA) (19), and we added analyses of this new dataset. At the same time, we obtained a larger training dataset from the ENCODE which consists of 167 samples with both DNase-seq and RNA-seq data (**Dataset S1**,

downloaded from https://www.encodeproject.org/), and BIRD was updated by retraining the model using these latest training data. For these latest training data, the aligned DNase-seq reads (aligned to hg19) and processed RNA-seq gene expression values (FPKM) were downloaded from the ENCODE and processed similar to the old training data. The updated BIRD trained using these latest training data was applied to analyze the HCA bone marrow dataset.

The HCA human bone marrow scRNA-seq data from two different donors (i.e., BM1 and BM6) were downloaded from the HCA website (https://preview.data.humancellatlas.org/). The raw data were processed and aligned to reference genome hg19 using CellRanger (20) to obtain read count for each gene. Cells with at least 1000 expressed genes (expressed genes are genes with non-zero read count) were retained. For each donor, the scVI (21) software was run with its default setting to obtain the imputed gene expression values for each single cell. The scVI imputed values were then used as the input for BIRD to predict chromatin accessibility.

In order to benchmark BIRD, one needs to have cells' cell type information. Because the true cell type label for each cell is unknown in this dataset, we inferred it computationally as follows.

First, we jointly clustered cells from the two donors into relatively homogeneous cell subpopulations. To do so, for each donor we computed the mean and variance of each gene across all cells. We then fitted a generalized additive model using all genes to describe the variance-mean relationship. Residuals from the fitted model represent genes' variance that cannot be explained by the mean. Using the third quartile of these variance residuals, we identified the top 25% most variable genes similar to (22). We further filtered out genes with low expression (i.e., genes with imputed expression < 1 in more than 90% of cells). The common variable genes found in both donors were used to align cells from the two donors using the mutual nearest neighbor (MNN) approach (23). The first two principal components (PCs) of the aligned dataset were used to cluster cells using k-means clustering (**Fig. S8b-c**). The number of clusters was chosen as the smallest k (k=33) that yields 1% unexplained variance (i.e., [within cluster sum of squares]/[total sum of squares] < 1%). Note that MNN was used only to align and cluster cells to facilitate visual comparison between the two donors. BIRD prediction was still based on scVI imputed values before MNN correction. Thus, the biological variation and batch effects were retained in the input gene expression data for BIRD.

Next, we assigned cell type labels to cell clusters by using the bulk gene expression data from 13 purified normal hematopoietic cell types obtained by FACS sorting (24). The processed bulk RNA-seq data (i.e., read counts for each gene) for 13 normal hematopoietic cell types were downloaded from GEO (GSE74246) and converted into FPKM values. Then, marker genes for each cell type were identified using limma (25) to find the top 100 genes most upregulated in each cell type compared to all other cell types. The union of all marker genes was obtained, and this set of genes was used for annotating cell type. For each cell cluster, we first calculated the Spearman's correlation between each single cell and each bulk RNA-seq sample. Here each bulk sample represents a purified cell type. A temporary cell type label was assigned to each cell by the cell type of the bulk sample that was most correlated to it. For each donor, if more than 60% of cells in a cluster share the same temporary cell type, then that cell cluster was temporary labeled as this cell type. If a cell cluster had the same temporary cell type label in both

donors, then the cell cluster was annotated with the cell type. Correspondingly, all cells in the cluster were also labeled as this cell type. If a cell cluster lacked a dominant temporary cell type (i.e., one cannot find a temporary cell type that accounts for 60% or more cells), or if the cluster's temporary cell type label was inconsistent between the two donors, then this cell cluster cannot be unambiguously annotated and will be excluded from our benchmark analysis.

Using the approach described above, and by intersecting scRNA-seq, bulk ATAC-seq and scATAC-seq data, we obtained 7 cell clusters that (1) were unambiguously and consistently annotated by bulk RNA-seq in both donors, (2) had both scRNA-seq and bulk and single-cell ATAC-seq data, and (3) whose corresponding cell type was not included in the training data. These clusters correspond to three cell types, CMP, GMP, and MEP. Since multiple clusters can be annotated with the same cell type, we consolidated the 7 clusters into 3 cell types by merging clusters annotated as the same cell type.

We applied BIRD to predict chromatin accessibility for the single cells from these three cell types based on the scVI-imputed gene expression data and compared the results with scATAC-seq. We first asked how well one can predict the chromatin accessibility in each cell type by using the bulk ATAC-seq signals from the corresponding cell type as the gold standard. The bulk ATAC-seq data (24) were downloaded from GEO (GSE74912) and processed similar to the ATAC-seq data used in the previous sections. The scATAC-seq data (26) were downloaded from GEO (GSE96772) and processed similar to the scATAC-seq dataset 2 (i.e., ATAC2) in the previous sections. Only cells with >1000 aligned sequence reads were retained. For each cell type, we randomly sampled *k* cells (*k*= 1, 5, 10, 20) and calculated the average gene expression profile for the scRNA-seq data or the average chromatin accessibility profile for the scATAC-seq data. Then, BIRD was applied to predict chromatin accessibility based on the gene expression data. The prediction performance was evaluated in terms of the Pearson's correlation between the predicted signals (or scATAC-seq measured signals) and the bulk ATAC-seq signals for each cell type. For *k*=5, 10, and 20, the random sampling was repeated 10 times. The mean and standard deviation (SD) of the results from the 10 analyses were shown in **Figure 8a-c**. For *k*=1, the analysis was performed for each single cell.

We then tested whether differential chromatin accessibility between different cell types can be predicted. We randomly sampled *k* cells (*k*= 1, 5, 10, 20) from each cell type and calculated the average gene expression profile for the scRNA-seq data or the average chromatin accessibility profile for the scATAC-seq data. Then, BIRD was applied to predict chromatin accessibility based on the gene expression data, and the difference in the predicted values between two cell types were computed. The performance was evaluated by computing the Pearson's correlation between the BIRD-predicted or scATAC-seq-measured differential signals and the gold standard bulk ATAC-seq differential signals for all possible pairs of cell types (i.e., CMP vs. GMP, CMP vs. MEP, and GMP vs. MEP). For each *k*, the analysis was repeated 10 times. The mean and SD of the results were shown. **Figures 8d** and **S9a,c** show the differential analysis based on all DHSs. **Figures 8e** and **S9b,d** show the differential analysis based on differential DHSs only. Here, differential DHSs were defined as loci with |true log2-scale bulk ATAC-seq signal difference between the two cell types| > 1, after filtering out loci with log2 ATAC-seq signals smaller than 1 in both cell types.

In order to evaluate the gain of imputation, we have repeated the above analyses using gene expression values without scVI-imputation. The results shown in **Figure S10** indicate that using scVI-impuated gene expression data for prediction improved the prediction performance.

In **Figure 8f-h**, we performed pseudotime analysis on the scRNA-seq data to computationally construct cells' developmental trajectories. For each donor, we applied TSCAN (27) to the scVI-imputed single-cell gene expression data to construct pseudo-temporal trajectories. In the first two principal component space, the pseudo-temporal trajectories showed three clear lineages, corresponding to the differentiation from HSC and MPP to erythroid, myeloid and lymphoid lineages respectively (**Figs. 8f, S8b**: HSC and MPP were enriched in cell clusters in the middle of the PC space and the lineage-specific cell types were enriched in cell clusters at the end of each branch). For each cell, we then applied BIRD to predict chromatin accessibility. Next, we analyzed the predicted chromatin accessibility by SCRAT (28), a software tool for analyzing scATAC-seq data. SCRAT has compiled the motif sites in the human genome for 1044 human TFs. Using these motif sites, SCRAT predicted the binding activity of each TF in each cell by calculating the average chromatin accessibility across the motif sites. The resulting TF binding activities were then quantile normalized across all single cells. We examined three known lineage-specific transcription factors (TFs) involved in hematopoiesis, GATA1 (erythroid), CEBPD (myeloid), and FOXP1 (lymphoid). Their gene expression and BIRD predicted binding activities in the donor BM1 are shown in **Figure 8g** and **8h** respectively. Similar analysis in donor BM6 is shown in **Figure S11**. In both donors, BIRD correctly predicted the lineage-specific increase of TF binding activities.

Predicting chromatin accessibility using RNA-seq data from different batches

In order to evaluated whether BIRD is robust to batch effects, we first collected bulk RNA-seq data from three different labs for GM12878 (GSM1663002, GSM3612677, GSM958728) and H1 (GSM2680565, GSM1494448, GSM915328) (2, 29, 30, 31, 32). We then applied BIRD, the random prediction model ("BIRD-permute") and the mean DH profile of training data ("Mean") to these bulk RNA-seq samples to predict chromatin accessibility. The bulk DNase-seq data in the corresponding cell type was used as the gold standard to evaluate prediction accuracy. **Figure S3** shows the Pearson's correlation between the predicted and gold standard signals across all loci. BIRD performed similarly when applied to data generated by different labs, and BIRD predictions from different labs consistently outperformed predictions by BIRD-permute and mean DH. Notably, the difference in prediction performance between BIRD predictions from different labs was much smaller than the difference between BIRD and the other methods (i.e., BIRD-permute and mean DH). This indicates that BIRD is relatively robust to batch effects.

Next, we evaluated the robustness of BIRD to batch effects using scRNA-seq data. We obtained a new GM12878 and H1 scRNA-seq dataset from GEO (GSE81861), which was generated by a lab (33) different from the labs that generated the GM12878 and H1 scRNA-seq data used in the main article in **Figures 5-7**. This new dataset contained 96 GM12878 cells and 96 H1 cells obtained using the Fluidigm C1 system. We downloaded the processed gene expression (FPKM) data from GSE81861, and applied the same prediction and evaluation procedures to these new GM12878 and H1 cells. The bulk DNase-seq data in the corresponding cell type were

used as the gold standard. **Figure S7a-b** shows the prediction performance (i.e., correlation between the predicted and true chromatin accessibility across loci) as a function of pooled cell number in GM12878 and H1, respectively. BIRD predictions using scRNA-seq data from two different labs performed similarly. Again, the difference between BIRD predictions using data from different labs was much smaller than the difference between BIRD and scATAC-seq. In **Figure S7c-d**, we predicted differential chromatin accessibility between GM12878 and H1. As before, we computed the correlation of the predicted and true differential signals across all loci (**Fig. S7c**) and across differential loci (**Fig. S7d**) respectively. Again, the difference in predictions from different labs was much smaller than the difference between BIRD and scATAC-seq.

Finally, in the analysis of the HCA bone marrow scRNA-seq data generated by the 10x Genomics platform, the two donors (BM1 and BM6) were processed in two different batches. Their difference contains both biological difference between the donors and technical difference due to batch effects. We used data from the two donors to separately make predictions. **Figures 8a-e** and **S9** show that the difference in predictions from different donors was smaller than the difference between BIRD and scATAC-seq.

For small-cell-number RNA-seq, since we did not find data in the same cell type generated by different labs, we were unable to evaluate the impact of batch effects.

Collectively, our results indicate that BIRD is relatively robust to batch effects.

The influence of input sample heterogeneity on predicting chromatin landscape of a pure cell type

If a small-cell-number RNA-seq sample is not obtained by cell sorting, it may contain more than one cell type. Such a sample may arise when analyzing precious clinical samples or embryonic tissues. BIRD applied to such a sample will predict its bulk chromatin accessibility (i.e. the pooled chromatin profile of multiple cell types). In order to predict chromatin landscape of pure cell types, one needs to either sort the cells or use scRNA-seq.

If the majority of cells in a small-cell-number sample has the same cell type, BIRD prediction may still capture the chromatin profile of that cell type. In order to understand how the intrinsic heterogeneity in a small-cell-number sample affects the prediction accuracy for the dominant cell type, we performed an analysis by pooling different numbers of GM12878 and H1 single cells together to simulate heterogeneous small-cell-number samples. We first randomly sampled 0, 1, 2, …, 10 GM12878 cells and 10, 9, …, 0 H1 cells from the scRNA-seq data used in the main article in **Figures 5-7** (GSE44618 and GSE64016). Next, we mixed the GM12878 and H1 cells together by a ratio of 0/10, 1/9, …, and 10/0. For each cell mixture, the average RNA-seq profile across the 10 cells was used to mimic the RNA-seq profile of a heterogeneous sample. BIRD was then applied to this profile to predict chromatin accessibility. The bulk GM12878 and H1 DNase-seq data were used as the gold standard to evaluate the prediction performance. The analysis was performed on 10 random samplings of cells. The mean performance of the 10 runs and the SD were computed.

Figure S12a shows the Pearson's correlation between the true GM12878 DNase-seq signals and the predicted GM12878 chromatin accessibility for different cell mixtures. The correlation increased when there were more GM12878 cells and fewer H1 cells in the mixtures. With 30% noisy cells (i.e. 3 H1 and 7 GM12878 cells), 84%

(=([current accuracy] – [min accuracy])/([max accuracy] – [min accuracy])=(0.68- 0.632)/(0.689- 0.632), **Fig. S12a**) of the prediction accuracy was retained. **Figure S12b** shows the Pearson's correlation between the true H1 DNase-seq signals and the predicted H1 chromatin accessibility for different cell mixtures. The correlation decreased when there were more GM12878 cells and fewer H1 cells in the mixtures. With 30% noisy cells (i.e. 3 GM12878 and 7 H1 cells), 89% (=(0.749-0.62)/(0.765-0.62), **Fig. S12b**) of the prediction accuracy was retained.

We also predicted differential chromatin accessibility between GM12878 and H1 using different cell mixtures and compared the prediction with the true GM12878-H1 differential DNase-seq signals. To predict differential chromatin, a cell mixture with x (=1, …, 10) GM12878 cells and $10-x$ H1 cells was compared to a pure H1 sample (by pooling another set of 10 H1 cells together). We calculated the Pearson's correlation between the predicted and true difference across all loci (**Fig. S12c**) and differential loci (**Fig. S12d**), respectively. Similar to **Figure S12a**, the correlation increased when there were more GM12878 cells in the mixtures. With 30% noisy cells (i.e. 3 H1 and 7 GM12878 cells in the mixed sample), 88% (=(0.403-0.156)/(0.436-0.156), **Fig. S12c**) and 91% (=(0.535-0.234)/(0.565-0.234), **Fig. S12d**) prediction accuracy was retained in the all loci and differential loci analyses, respectively.

In summary, when the dominant cell type accounted for more than 70% of the cells in the sample, the prediction remained relatively accurate compared to what one would expect from the pure cell type.

Prediction accuracy as a function of the similarity between the test and training samples

To evaluate how the similarity between the test and training samples influences BIRD prediction accuracy, we defined a distance measure to characterize the similarity as follows. First, using training data, co-expressed genes are grouped into 1000 clusters as in BIRD. For each RNA-seq sample, the average expression of genes in each cluster is computed. In this way, the normalized gene expression profile of each RNA-seq sample is converted into a 1000-dimensional feature vector consisting of the mean expression of each and every gene cluster. This step summarizes information from co-expressed genes and can mitigate the sparsity in the scRNA-seq data. Next, using the feature vector defined in this way, the Pearson's correlation between any two samples can be computed. For a new test RNA-seq sample, we will identify the top 10% samples in the training RNA-seq data that are most correlated with the test sample. The distance between the new test sample and the training data, also called "training-test distance", is defined as 1 – [the average of the correlation between the test sample and the top 10% most correlated samples in the training data]. An increase in distance implies a decrease in the similarity between test and training samples. A function to calculate training-test distance is provided in the latest BIRD software.

We then examined the relationship between the training-test distance and prediction accuracy. To this end, a five-fold cross-validation analysis was run using the latest 167 ENCODE training samples. In each fold of the analysis, BIRD trained using the training data was applied to make predictions on the test samples, and the training-test distance between each test sample and the training data was computed. **Figure S13a** shows prediction accuracy (i.e., correlation between the predicted and true values across all loci) for each test sample versus its training-test distance. As the training-test distance increases, the prediction accuracy decreases. Overall, the prediction accuracy is relatively high and stable when the distance is smaller than 0.5, and the accuracy starts to

drop faster when the distance is between 0.5 and 0.8. Of note, the training-test distances for RNA-seq data used in our analyses were in the range of 0.6 to 0.8 (**Fig. S13b**).

References

1. Roadmap Epigenomics Consortium. (2015) Integrative analysis of 111 reference human epigenomes. *Nature,* **518,** 317-330.

2. ENCODE Project Consortium. (2012) An integrated encyclopedia of DNA elements in the human genome. *Nature,* **489,** 57-74.

3. Langmead,B., Trapnell,C., Pop,M. and Salzberg,S.L. (2009) Ultrafast and memory-efficient alignment of short DNA sequences to the human genome. *Genome Biol.,* **10,** R25.

4. Trapnell,C., Williams,B.A., Pertea,G., Mortazavi,A., Kwan,G., Van Baren,M.J., Salzberg,S.L., Wold,B.J. and Pachter,L. (2010) Transcript assembly and quantification by RNA-seq reveals unannotated transcripts and isoform switching during cell differentiation. *Nat. Biotechnol.,* **28,** 511-515.

5. Harrow,J., Frankish,A., Gonzalez,J.M., Tapanari,E., Diekhans,M., Kokocinski,F., Aken,B.L., Barrell,D., Zadissa,A., Searle,S., et al. (2012) GENCODE: The reference human genome annotation for the ENCODE project. *Genome Res.,* **22,** 1760-1774.

6. Kim,D., Pertea,G., Trapnell,C., Pimentel,H., Kelley,R. and Salzberg,S.L. (2013) TopHat2: Accurate alignment of transcriptomes in the presence of insertions, deletions and gene fusions. *Genome Biol.,* **14,** r36.

7. Zhou,W., Sherwood,B., Ji,Z., Xue,Y., Du,F., Bai,J., Ying,M. and Ji,H. (2017) Genome-wide prediction of DNase I hypersensitivity using gene expression. *Nature Communications,* **8,** 1038.

8. Hartigan,J.A. and Wong,M.A. (1979) Algorithm AS 136: A k-means clustering algorithm. *Applied Statistics,* **28,** 100-108.

9. Ernst,J. and Kellis,M. (2015) Large-scale imputation of epigenomic datasets for systematic annotation of diverse human tissues. *Nat. Biotechnol.,* **33,** 364-376.

10. Zhang,Y., Liu,T., Meyer,C.A., Eeckhoute,J., Johnson,D.S., Bernstein,B.E., Nusbaum,C., Myers,R.M., Brown,M., Li,W., et al. (2008) Model-based analysis of ChIP-seq (MACS). *Genome Biol.,* **9,** r137. Epub 2008 Sep 17.

11. Thurman,R.E., Rynes,E., Humbert,R., Vierstra,J., Maurano,M.T., Haugen,E., Sheffield,N.C., Stergachis,A.B., Wang,H. and Vernot,B. (2012) The accessible chromatin landscape of the human genome. *Nature,* **489,** 75-82.

12. Wang,Z., Zang,C., Rosenfeld,J.A., Schones,D.E., Barski,A., Cuddapah,S., Cui,K., Roh,T.Y., Peng,W., Zhang,M.Q., et al. (2008) Combinatorial patterns of histone acetylations and methylations in the human genome. *Nat. Genet.,* **40,** 897-903.

13. Matys,V., Kel-Margoulis,O.V., Fricke,E., Liebich,I., Land,S., Barre-Dirrie,A., Reuter,I., Chekmenev,D., Krull,M., Hornischer,K., et al. (2006) TRANSFAC and its module TRANSCompel: Transcriptional gene regulation in eukaryotes. *Nucleic Acids Res.,* **34,** 108.

14. Mathelier,A., Zhao,X., Zhang,A.W., Parcy,F., Worsley-Hunt,R., Arenillas,D.J., Buchman,S., Chen,C.Y., Chou,A., Ienasescu,H., et al. (2014) JASPAR 2014: An extensively expanded and updated open-access database of transcription factor binding profiles. *Nucleic Acids Res.,* **42,** 142.

15. Ji,H., Jiang,H., Ma,W., Johnson,D.S., Myers,R.M. and Wong,W.H. (2008) An integrated software system for analyzing ChIP-chip and ChIP-seq data. *Nat. Biotechnol.,* **26,** 1293-1300.

16. Storey,J.D., Bass,A.J., Dabney,A. and Robinson,D. (2015) Qvalue: Q-value estimation for false discovery rate control. *R Package Version 2.2.2.*

17. Cao,Z., Chen,C., He,B., Tan,K. and Lu,C. (2015) A microfluidic device for epigenomic profiling using 100 cells. *Nature Methods,* **12,** 959-962.

18. He,H.H., Meyer,C.A., Shin,H., Bailey,S.T., Wei,G., Wang,Q., Zhang,Y., Xu,K., Ni,M., Lupien,M., et al. (2010) Nucleosome dynamics define transcriptional enhancers. *Nat. Genet.,* **42,** 343-347.

19. Regev,A., Teichmann,S.A., Lander,E.S., Amit,I., Benoist,C., Birney,E., Bodenmiller,B., Campbell,P., Carninci,P., Clatworthy,M., et al. (2017) The human cell atlas. *Elife,* **6,** 10.7554/eLife.27041.

20. Zheng,G.X., Terry,J.M., Belgrader,P., Ryvkin,P., Bent,Z.W., Wilson,R., Ziraldo,S.B., Wheeler,T.D., McDermott,G.P., Zhu,J., et al. (2017) Massively parallel digital transcriptional profiling of single cells. *Nat. Commun.,* **8,** 14049.

21. Lopez,R., Regier,J., Cole,M.B., Jordan,M.I. and Yosef,N. (2018) Deep generative modeling for single-cell transcriptomics. *Nat. Methods,* **15,** 1053-1058.

22. Guo,G., Pinello,L., Han,X., Lai,S., Shen,L., Lin,T., Zou,K., Yuan,G. and Orkin,S.H. (2016) Serum-based culture conditions provoke gene expression variability in mouse embryonic stem cells as revealed by single-cell analysis. *Cell Reports,* **14,** 956-965.

23. Haghverdi,L., Lun,A.T.L., Morgan,M.D. and Marioni,J.C. (2018) Batch effects in single-cell RNA-sequencing data are corrected by matching mutual nearest neighbors. *Nat. Biotechnol.,* **36,** 421-427.

24. Corces,M.R., Buenrostro,J.D., Wu,B., Greenside,P.G., Chan,S.M., Koenig,J.L., Snyder,M.P., Pritchard,J.K., Kundaje,A., Greenleaf,W.J., et al. (2016) Lineage-specific and single-cell chromatin accessibility charts human hematopoiesis and leukemia evolution. *Nat. Genet.,* **48,** 1193-1203.

25. Ritchie,M.E., Phipson,B., Wu,D., Hu,Y., Law,C.W., Shi,W. and Smyth,G.K. (2015) Limma powers differential expression analyses for RNA-sequencing and microarray studies. *Nucleic Acids Res.,* **43,** e47.

26. Buenrostro,J.D., Corces,M.R., Lareau,C.A., Wu,B., Schep,A.N., Aryee,M.J., Majeti,R., Chang,H.Y. and Greenleaf,W.J. (2018) Integrated single-cell analysis maps the continuous regulatory landscape of human hematopoietic differentiation. *Cell,* **173,** 1548.e16.

27. Ji,Z. and Ji,H. (2016) TSCAN: Pseudo-time reconstruction and evaluation in single-cell RNA-seq analysis. *Nucleic Acids Res.,* **44,** e117.

28. Ji,Z., Zhou,W. and Ji,H. (2017) Single-cell regulome data analysis by SCRAT. *Bioinformatics,* **33,** 2930-2932.

29. Cusanovich,D.A., Daza,R., Adey,A., Pliner,H.A., Christiansen,L., Gunderson,K.L., Steemers,F.J., Trapnell,C. and Shendure,J. (2015) Multiplex single-cell profiling of chromatin accessibility by combinatorial cellular indexing. *Science,* **348,** 910-914.

30. Sarma,M., Lee,J., Ma,S., Li,S. and Lu,C. (2019) A diffusion-based microfluidic device for single-cell RNA-seq. *Lab. Chip,* **19,** 1247-1256.

31. Li,Y., Brauer,P.M., Singh,J., Xhiku,S., Yoganathan,K., Zuniga-Pflucker,J.C. and Anderson,M.K. (2017) Targeted disruption of TCF12 reveals HEB as essential in human mesodermal specification and hematopoiesis. *Stem Cell. Reports,* **9,** 779-795.

32. Sperber,H., Mathieu,J., Wang,Y., Ferreccio,A., Hesson,J., Xu,Z., Fischer,K.A., Devi,A., Detraux,D., Gu,H., et al. (2015) The metabolome regulates the epigenetic landscape during naive-to-primed human embryonic stem cell transition. *Nat. Cell Biol.,* **17,** 1523-1535.

33. Li,H., Courtois,E.T., Sengupta,D., Tan,Y., Chen,K.H., Goh,J.J.L., Kong,S.L., Chua,C., Hon,L.K., Tan,W.S., et al. (2017) Reference component analysis of single-cell transcriptomes elucidates cellular heterogeneity in human colorectal tumors. *Nat. Genet.,* **49,** 708-718.

SUPPLEMENTARY DATASET

Dataset S1. Information of data used for model training and testing. This dataset contains four sheets. (**a**) List of DNase-seq and RNA-seq data from the Epigenome Roadmap used for training and testing BIRD model. The data contains 70 samples representing 30 different cell types. The table shows the cell types, GEO accession numbers (GSM number) of the DNase-seq and RNA-seq samples, and the donor identifiers. One cell type can contain multiple samples from different donors. (**b**) Epigenome identifiers (IDs) in ChromImpute and the corresponding cell type names and GSM number of the samples in BIRD for the 17 cell types used to compare ChromImpute and BIRD. (**c**) Transcription factor binding motifs and their TRANSFAC or JASPAR accession numbers used in this study. (**d**) List of DNase-seq and RNA-seq data from the ENCODE used for training the latest BIRD model. The data contains 167 samples from 74 cell types.

SUPPLEMENTARY TABLE

Table S1. Cell isolation and sequencing platform information for the test bulk RNA-seq, small-cell-number RNA-seq, single-cell RNA-seq, bulk ATAC-seq, and single-cell ATAC-seq data used in this study.

Dataset	Cell isolation method	Sequencing platform	Cell number	Number of gene detected per cell (count > 0): mean (min,max)	Used in Figures	Ref.
GM12878 scRNA-seq (GSE44618)	glass micropipette	Illumina HiSeq 2000	28	11527 (7724,17506)	5, 6, 7, S6, S7, S12, S13	main ref (3)
GM12878 scRNA-seq (GSE81861)	Fluidigm C1	Illumina HiSeq 2000	96	14260 (10574, 17220)	S7, S13	main ref (35)
H1 scRNA-seq (GSE64016)	Fluidigm C1	Illumina HiSeq 2500	62	21489 (15699, 22588)	5, 6, 7, S7, S12, S13	main ref (34)
H1 scRNA-seq (GSE81861)	Fluidigm C1	Illumina HiSeq 2000	96	15391 (13073, 17441)	S7, S13	main ref (35)
HCA-BM1 scRNA-seq (https://preview.data.humancellatlas.org)	10x Genomics	Illumina Hiseq X 10	5915	2280 (1000, 8350)	8, S8, S9, S10, S13	main ref (18)
HCA-BM6 scRNA-seq (https://preview.data.humancellatlas.org)	10x Genomics	Illumina Hiseq X 10	5869	2191 (1000, 7196)	8, S8, S9, S10, S11, S13	main ref (18)
GM12878 bulk RNA-seq (GSM958728)	NA	Illumina Genome Analyzer	NA	28008	3, S3, S4, S13	main ref (23)
GM12878 bulk RNA-seq (GSM1663002)	NA	Illumina NextSeq 500	NA	23207	S3, S13	main ref (9)
GM12878 bulk RNA-seq (GSM3612677)	NA	Illumina Hiseq X 10	NA	10268	S3, S13	suppl ref (30)
H1 bulk RNA-seq (GSM915328)	NA	Illumina HiSeq 2000	NA	28980	S3, S13	main ref (23)
H1 bulk RNA-seq (GSM2680565)	NA	Illumina HiSeq 2000	NA	16487	S3, S13	suppl ref (31)
H1 bulk RNA-seq (GSM1494448)	NA	Illumina HiSeq 2000	NA	19301	S3, S13	suppl ref (32)
GM12878 small-cell-number RNA-seq (100 cells, GSM1087856, GSM1087857)	NA	Illumina HiSeq 2000	NA	23692	3, S4, S13	main ref (3)
GM12878 small-cell-number RNA-seq (30 cells, GSM1087858, GSM1087859)	NA	Illumina HiSeq 2000	NA	23160	3, 4, S4, S13	main ref (3)

Dataset	Cell isolation method	Sequencing platform	Cell number	Number of reads per cell: mean (min,max)	Used in Figures	Ref
GM12878 scATAC-seq (GSM1647121)	combinatorial cellular indexing	Illumina MiSeq	222	2610 (508, 19231)	5, 6	main ref (9)
GM12878 scATAC-seq (GSE65360)	Fluidigm C1	Illumina MiSeq	340	10590 (547, 156320)	5, 6, 7, S7	main ref (10)
H1 scATAC-seq (GSE65360)	Fluidigm C1	Illumina MiSeq	90	14706 (633, 147648)	5, 6, 7, S7	main ref (10)
CMP scATAC-seq (GSE96772)	Fluidigm C1	Illumina NextSeq 500	653	21936 (1100, 1022607)	8, S9, S10	main ref (32)
GMP scATAC-seq (GSE96772)	Fluidigm C1	Illumina NextSeq 500	258	13546 (1002, 228406)	8, S9, S10	main ref (32)
MEP scATAC-seq (GSE96772)	Fluidigm C1	Illumina NextSeq 500	194	31400 (1033, 348588)	8, S9, S10	main ref (32)
GM12878 bulk ATAC-seq (50,000 cells, GSE47753)	NA	Illumina HiSeq 2000	NA	18633349	3, 4, 5, S4, S5, S6	main ref (7)
GM12878 bulk ATAC-seq (500 cells, GSE47753)	NA	Illumina HiSeq 2000	NA	5987579	3, 4, S4, S5, S6	main ref (7)

SUPPLEMENTARY FIGURES

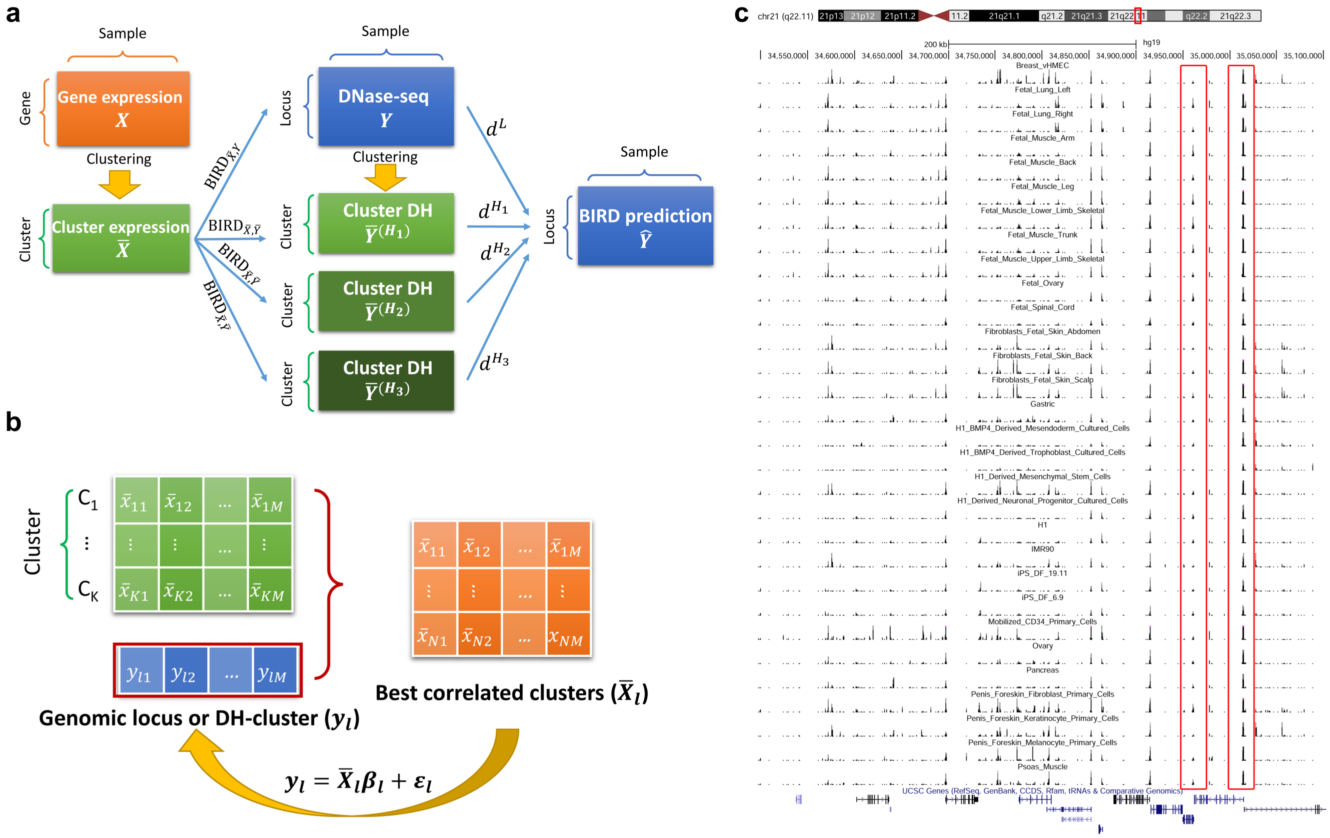

Figure S1. Overview of BIRD prediction model and locus effects in DNase-seq data. (**a**) BIRD groups co-expressed genes into gene-clusters and uses the clusters' mean expression levels as predictors. BIRD also groups genomic loci (i.e., DHSs) with similar cross-sample DH variation patterns into DHS-clusters. Then, BIRD builds regression models both for predicting the DH level at each genomic locus (i.e. $\mathrm{BIRD}_{\bar{X},Y}$) and for predicting the mean DH level of each DHS-cluster (i.e. $\mathrm{BIRD}_{\bar{X},\bar{Y}}$). The locus-level predictions and the cluster-level predictions are combined through model averaging to obtain the final predicted DH value for each locus. (**b**) For both $\mathrm{BIRD}_{\bar{X},Y}$ and $\mathrm{BIRD}_{\bar{X},\bar{Y}}$, predictors (i.e. gene-clusters) that best correlate with the response (i.e., DH level) are selected. The selected predictors are then used to build a linear regression model. (**c**) An example for locus effects in DNase-seq data. DNase-seq signals from a genomic region in 30 different Epigenome Roadmap cell types are shown (only one sample is shown for each cell type). The highlighted regions show that DH levels at some genomic loci tend to be consistently higher than the DH levels at other loci in almost all cell types. As a result, the mean DH level across all cell types can predict cross-locus DH variation in a new sample with good accuracy. We note that permuting sample labels will not change the mean DH level at each locus and therefore will not perturb the locus effects.

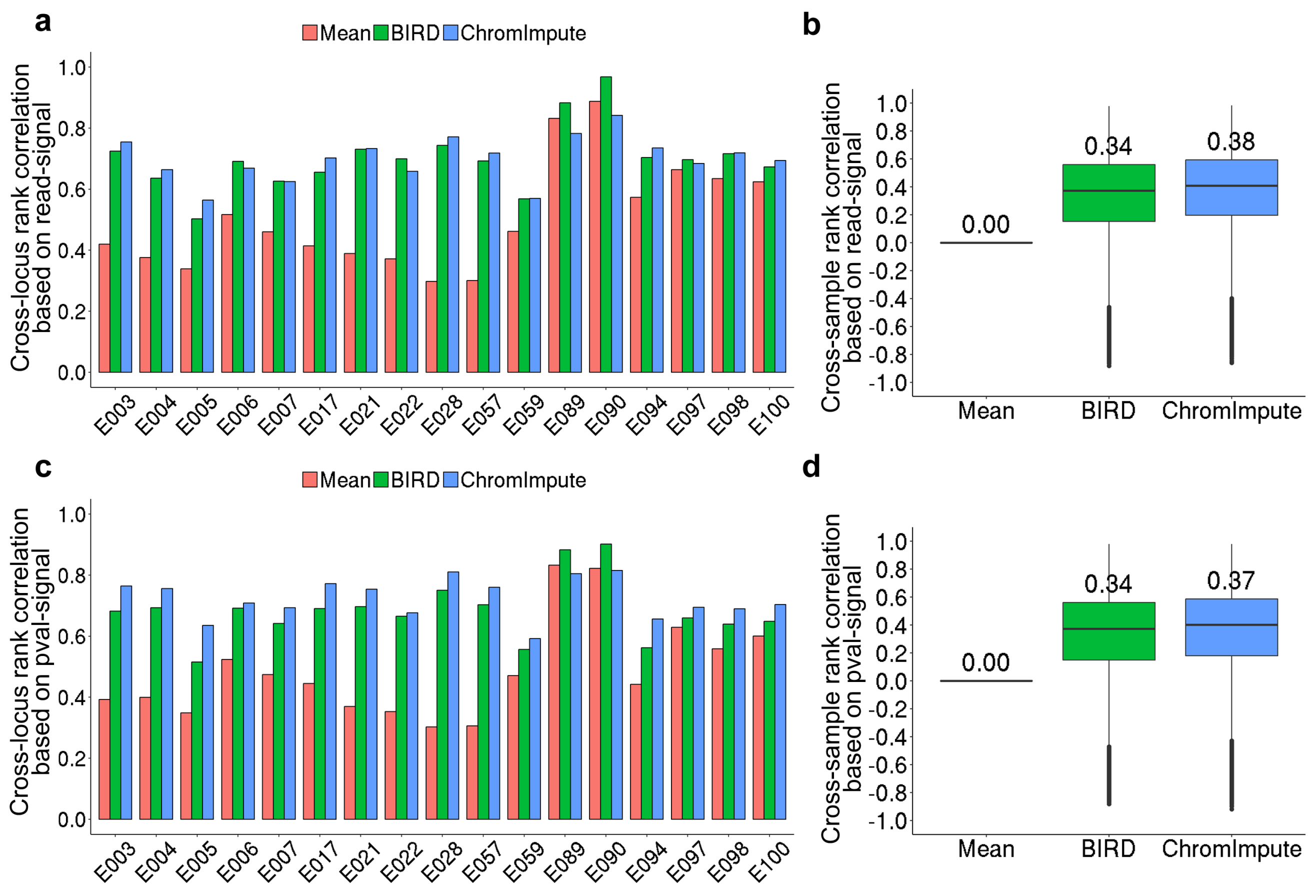

Figure S2. BIRD and ChromImpute comparison in 17 test cell types. (**a**) Spearman's rank correlation between the true read-signal and Mean-, BIRD- or ChromImpute- predicted signal across different loci in each cell type. (**b**) Distribution and mean of the Spearman's rank correlation between the true read-signal and Mean-, BIRD- or ChromImpute- predicted signal across the test cell types for each genomic locus. (**c**) Spearman's rank correlation between the true MACS pval-signal and Mean-, BIRD- or ChromImpute- predicted signal across different loci in each cell type. (**d**) Distribution and mean of Spearman's rank correlation between the true MACS pval-signal and Mean-, BIRD- or ChromImpute- predicted signal across the test cell types for each genomic locus. For all plots, Mean- and BIRD- predicted signals are read signals, whereas ChromImpute-predicted signals are pval-signals. In **a** and **c**, labels in X-axis are epigenome identifiers (IDs) in ChromImpute. The cell type name corresponding to each ID is listed in **Dataset S1**.

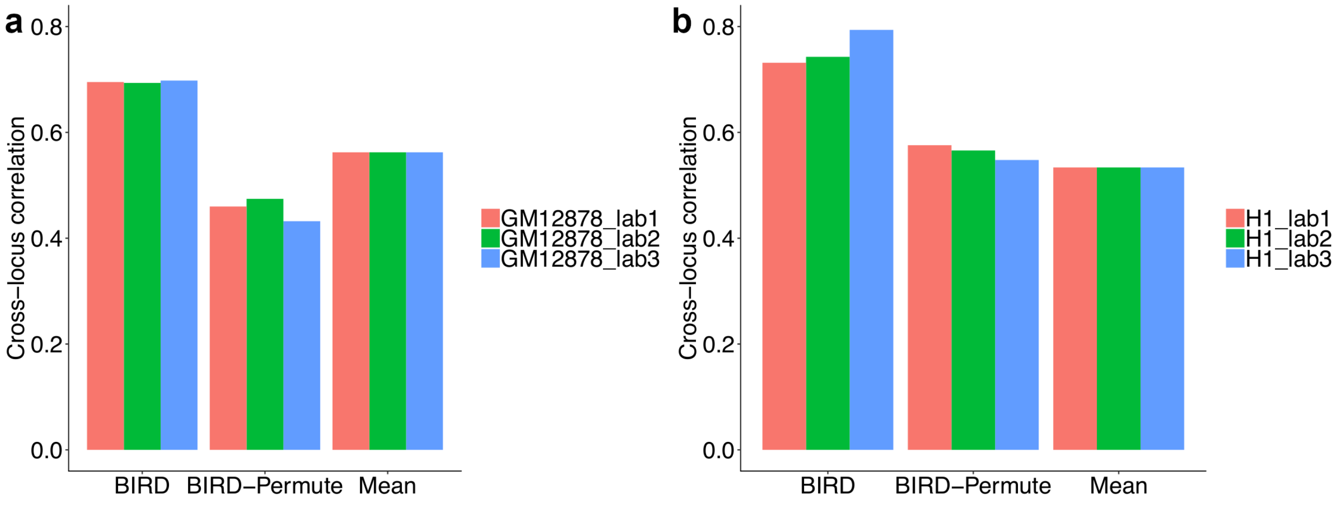

Figure S3. Impacts of batch effects on BIRD prediction accuracy in bulk RNA-seq data. (**a**) Prediction performance using GM12878 bulk RNA-seq data generated by three different labs for three prediction methods: BIRD, random prediction models ("BIRD-permute") and the mean DH profile of training data ("Mean"). (**b**) Prediction performance using H1 bulk RNA-seq data generated by three different labs for BIRD, BIRD-Permute, and Mean. Prediction performance is measured by the Pearson's correlation between the predicted DH and gold standard DNase-seq signals across all loci.

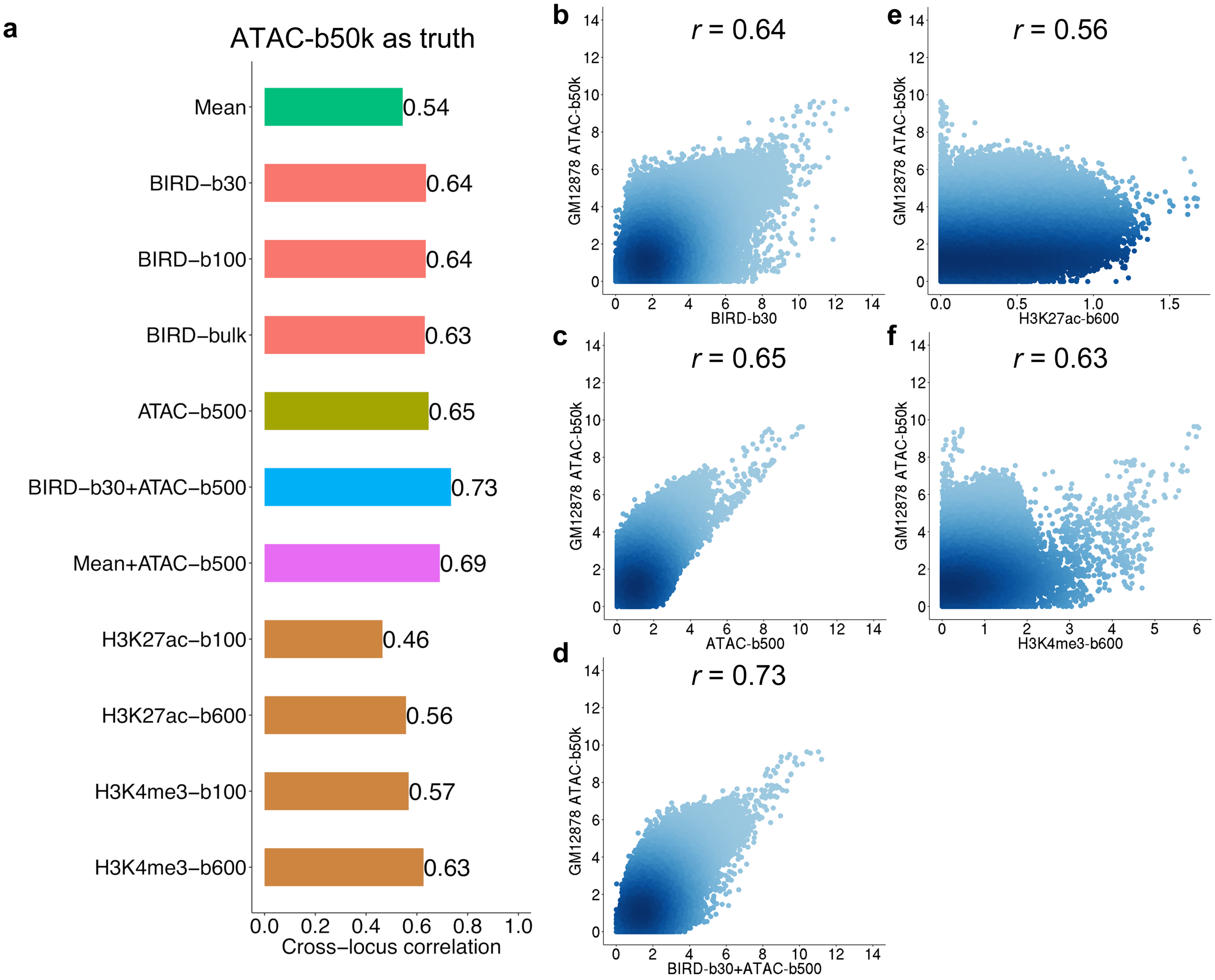

Figure S4. Performance of predicting DH in GM12878 using small-cell-number RNA-seq. The performance is evaluated using ATAC-seq with 50,000 cells as gold standard. (**a**) Pearson's correlation between ATAC-seq with 50,000 cells ("ATAC-b50k") and chromatin accessibility predicted or measured by different methods. "Mean": mean DH profile of training samples. "BIRD-b30" and "BIRD-b100": BIRD-predicted DH based on small-cell-number RNA-seq samples with 30 and 100 cells. "BIRD-bulk": BIRD-predicted DH based on bulk RNA-seq. "ATAC-b500": ATAC-seq with 500 cells. "BIRD-b30+ATAC-b500: average of BIRD-predicted DH from 30 cells and ATAC-seq from 500 cells. "Mean+ATAC-b500": average of mean DH profile of training samples and ATAC-seq from 500 cells. "H3K27ac-b100", "H3K27ac-b600", "H3K4me3-b100" and "H3K4me3-b600": MOWChIP-seq for histone modification H3K27ac or H3K4me3 with 100 or 600 cells. (**b**)-(**f**) Scatterplots comparing ATAC-b50k with chromatin accessibility predicted or measured by BIRD-b30, ATAC-b500, BIRD-b30+ATAC-b500, H3K27ac-b600 and H3K4me3-b600. Each dot is a genomic locus. Pearson's correlation is shown on top of each plot.

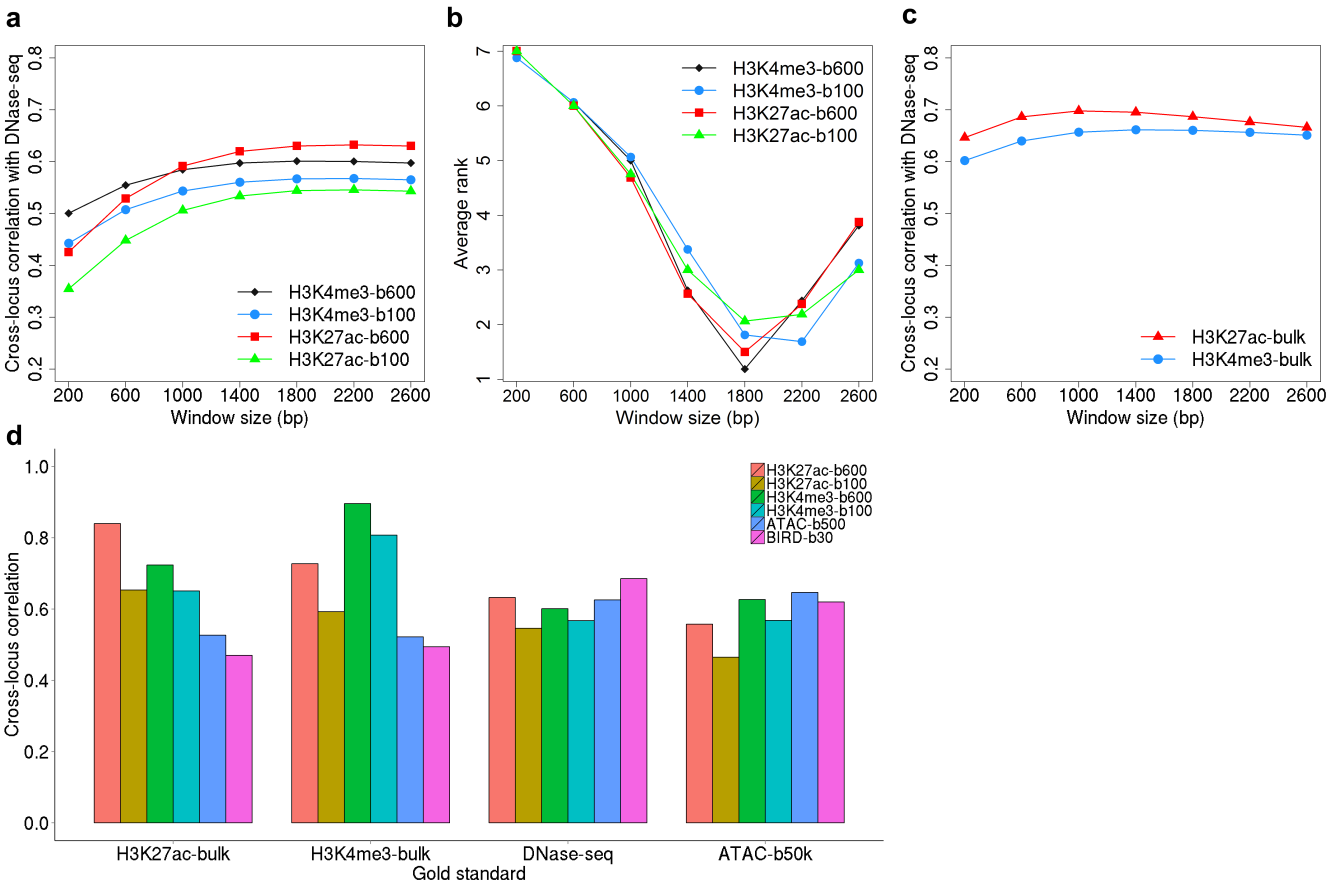

Figure S5. Window size selection for analyzing histone modification MOWChIP-seq and ChIP-seq data and correlation based on different gold standards. (**a**) Pearson's correlation between MOWChIP-seq and bulk DNase-seq signal for different window sizes (*W*). The optimal *W* that maximizes the correlation was 2200 for H3K27ac with 100 and 600 cells ("H3K27ac-b100", "H3K27ac-b600"), 2200 for H3K4me3 with 100 cells ("H3K4me3-b100") and 1800 for H3K4me3 with 600 cells ("H3K4me3-b600"). (**b**) TFBS prediction performance for different window sizes using MOWChIP-seq. The optimal *W* that maximizes the TFBS prediction performance (in terms of the smallest average rank) was 1800 for H3K27ac-b100 and H3K27ac-b600, 2200 for H3K4me3-b100 and 1800 for H3K4me3-b600. (**c**) Pearson's correlation between bulk ChIP-seq and bulk DNase-seq signal for different window sizes. The optimal *W* that maximizes the correlation was 1000 for bulk H3K27ac ("H3K27ac-bulk") and 1400 for bulk H3K4me3 ("H3K4me3-bulk"). (**d**) Correlation between gold standard signal in GM12878 and signals from small-cell-number MOWChIP-seq, ATAC-seq and BIRD. Different types of gold standard are used. Each group of bars corresponds to one type of gold standard. The gold standard signals include bulk H3K27ac ChIP-seq data ("H3K27ac-bulk"), bulk H3K4me3 ChIP-seq data ("H3K4me3-bulk"), bulk DNase-seq data ("DNase-seq") and ATAC-seq data from 50,000 cells ("ATAC-b50k"). Compared signals include MOWChIP-seq data for H3K27ac and H3K4me3 obtained from 100 and 600 cells ("H3K27ac-b100", "H3K27ac-b600", "H3K4me3-b100" and "H3K3me3-b600"), ATAC-seq from 500 cells ("ATAC-b500"), and BIRD predictions based on RNA-seq from 30 cells ("BIRD-b30").

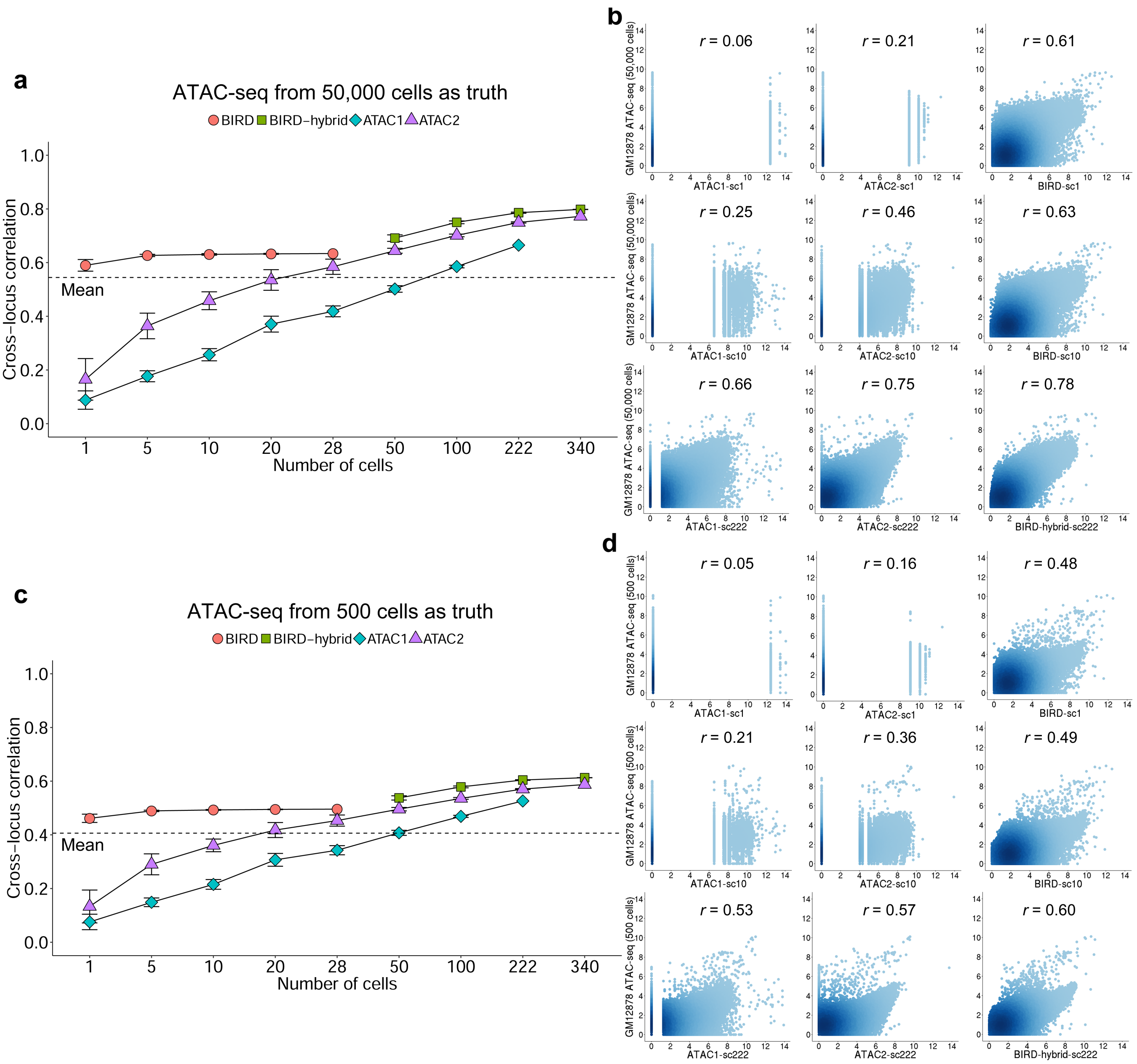

Figure S6. Performance for predicting chromatin accessibility using pooled single-cell RNA-seq in GM12878 evaluated using ATAC-seq with 50,000 cells ("ATAC-b50k") or 500 cells ("ATAC-b500") as gold standard. (**a**) Pearson's correlation between ATAC-seq with 50,000 cells and chromatin accessibility predicted or measured by different single-cell methods. The correlation is shown as a function of pooled cell number. Error bars are standard deviation based on 10 independent samplings of cells (**Methods**). "ATAC1": scATAC-seq dataset 1. "ATAC2": scATAC-seq dataset 2. "BIRD": BIRD-predicted DH using single-cell RNA-seq. "BIRD-hybrid": the average of BIRD-predictions based on 28 cells and pooled ATAC-seq from scATAC-seq dataset 2 (here x-axis is the total number of cells used by scRNA-seq and scATAC-seq). Prediction performance using the mean DH profile of training samples ("Mean") is shown as a dashed line. (**b**) Scatterplots comparing ATAC-seq with 50,000 cells with signals from ATAC1 and ATAC2 using 1 cell ("ATAC1-sc1", "ATAC2-sc1"), 10 cells ("ATAC1-sc10", "ATAC2-sc10") and 222 cells ("ATAC1-sc222", "ATAC2-sc222"), BIRD prediction using 1 cell ("BIRD-sc1"), 10 cells ("BIRD-sc10") and BIRD-hybrid using 222 cells ("BIRD-hybrid-sc222"). (**c**)-(**d**) The same as (**a**)-(**b**) but using ATAC-seq with 500 cells as gold standard.

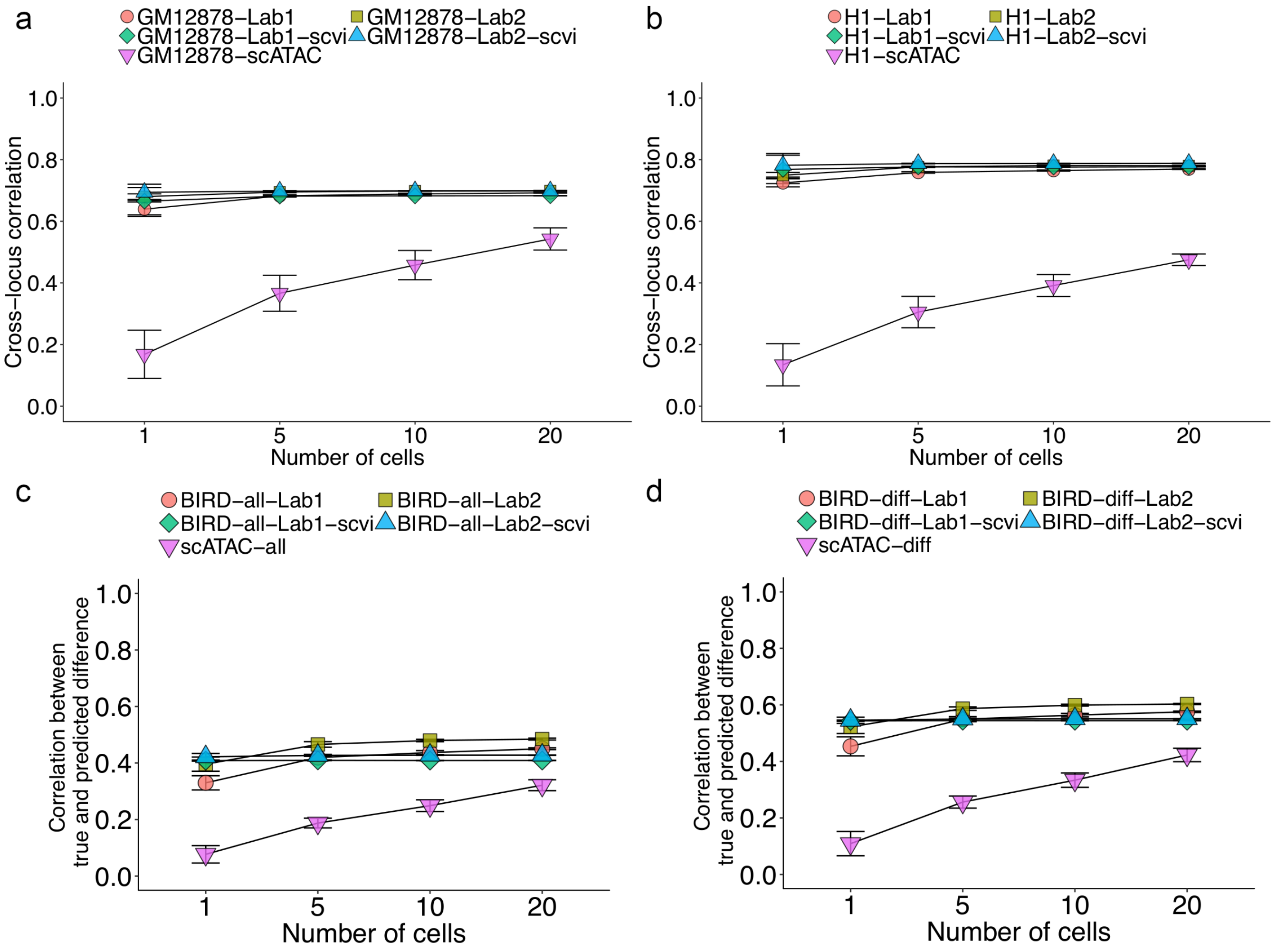

Figure S7. Impacts of batch effects and imputation on BIRD prediction accuracy in GM12878 and H1 scRNA-seq data. (**a**) Prediction performance of BIRD using GM12878 single-cell RNA-seq data generated by two different labs and a comparison to GM12878 single-cell ATAC-seq by pooling different number of cells. BIRD prediction is performed using the original data without imputation (GM12878-Lab1 and GM12878-Lab2) or data after scVI-imputation (GM12878-Lab1-scvi and GM12878-Lab2-scvi). (**b**) Prediction performance of BIRD using H1 single-cell RNA-seq data generated by two different labs and a comparison to H1 single-cell ATAC-seq data by pooling different number of cells. BIRD prediction is performed using the original data without imputation (H1-Lab1 and H1-Lab2) or data after scVI-imputation (H1-Lab1-scvi and H1-Lab2-scvi). (**c**) Comparison between BIRD (using scRNA-seq data generated by two different labs) and scATAC-seq for predicting GM12878-H1 differential DNase-seq signals across all DHSs by using different number of cells. BIRD prediction is performed using the original data (BIRD-all-Lab1 and BIRD-all-Lab2) or data after scVI-imputation (BIRD-all-Lab1-scvi and BIRD-all-Lab2-scvi). (**d**) Comparison between BIRD (using scRNA-seq data generated by two different labs) and scATAC-seq for predicting GM12878-H1 differential DNase-seq signals at differential DHSs by using different number of cells. BIRD prediction is performed using the original data (BIRD-diff-Lab1 and BIRD-diff-Lab2) or data after scVI-imputation (BIRD-diff-Lab1-scvi and BIRD-diff-Lab2-scvi).

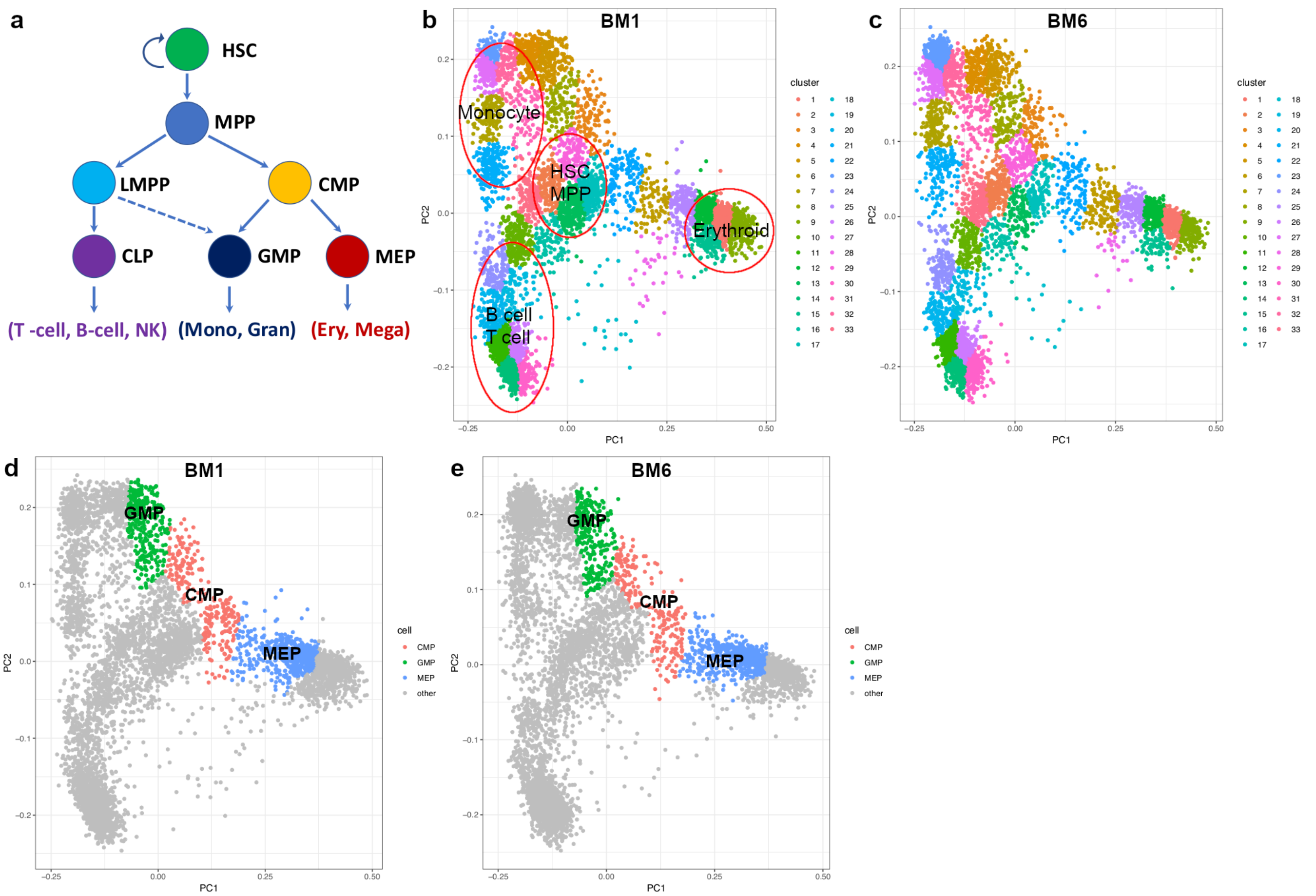

Figure S8. Cell type assignment for the HCA bone marrow dataset. (**a**) A schematic view of the differentiation hierarchy of cell types in human hematopoiesis. (**b-c**) The single cells are clustered into 33 clusters for (**b**) the BM1 donor sample and (**c**) the BM6 donor sample, jointly using the MNN aligned data. (**d-e**) CMP, GMP, and MEP are unambiguously and consistently annotated in both (**d**) BM1 and (**e**) BM6.

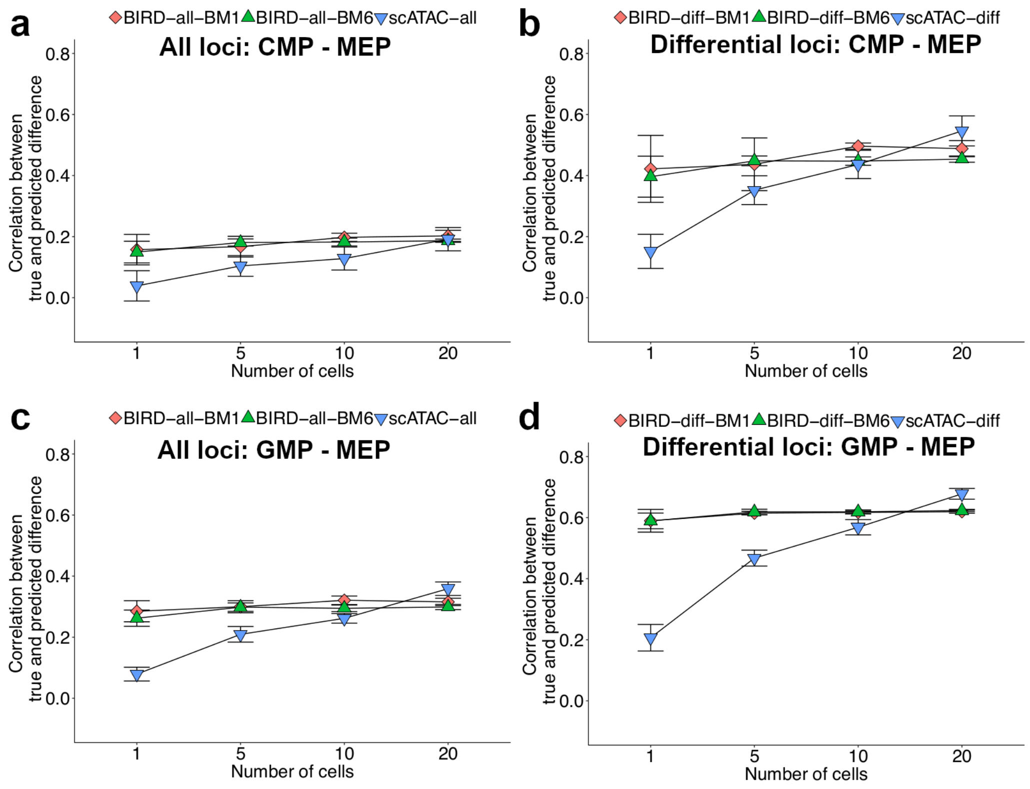

Figure S9. Pearson's correlation between the true bulk CMP-MEP (**a,b**) and GMP-MEP (**c,d**) differential ATAC-seq signals and BIRD predicted differential signals from scRNA-seq or scATAC-seq-measured signals across (**a,c**) all DHSs and (**b,d**) differential DHSs.

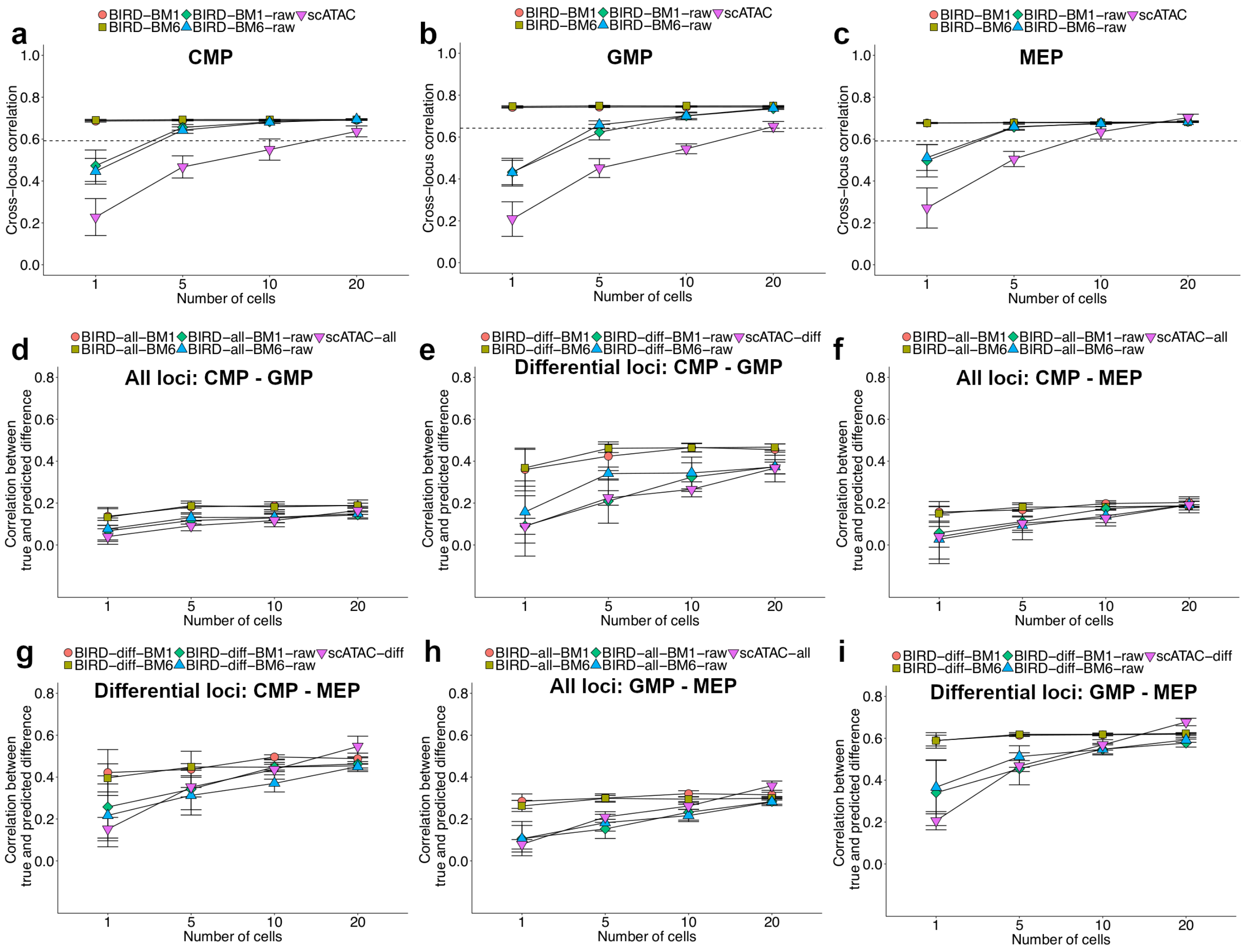

Figure S10. Comparison of BIRD prediction accuracy based on unimputed (raw) or scVI-imputed gene expression profiles in the HCA bone marrow dataset. (**a-c**) Pearson's correlation between the true bulk ATAC-seq signal and chromatin accessibility predicted by BIRD based on the raw and imputed gene expression by pooling different number of cells for (**a**) CMP, (**b**) GMP, and (**c**) MEP, respectively. (**d-i**) Pearson's correlation between the true bulk CMP-GMP, CMP-MEP, and GMP-MEP differential ATAC-seq signals and BIRD predicted differential signals across (**d,f,h**) all DHSs and (**e,g,i**) differential DHSs. Performance based on scATAC-seq measured signals is also included for comparison in each analysis.

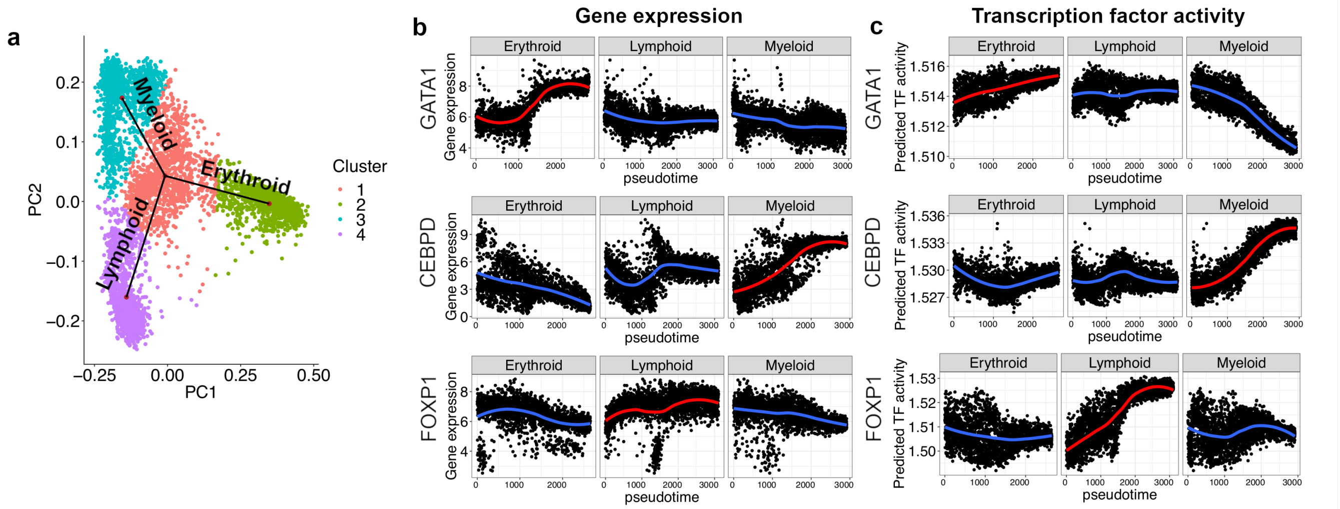

Figure S11. Pseudotime analysis for the HCA bone marrow sample BM6. (**a**) The first two principal components show three distinct lineages. (**b**) Gene expression of lineage-specific TFs, GATA1, CEBPD, and FOXP1 along pseudotime of the erythroid, lymphoid, and myeloid lineages. (**c**) The predicted TF activity of GATA1, CEBPD, and FOXP1 along pseudotime of the three lineages.

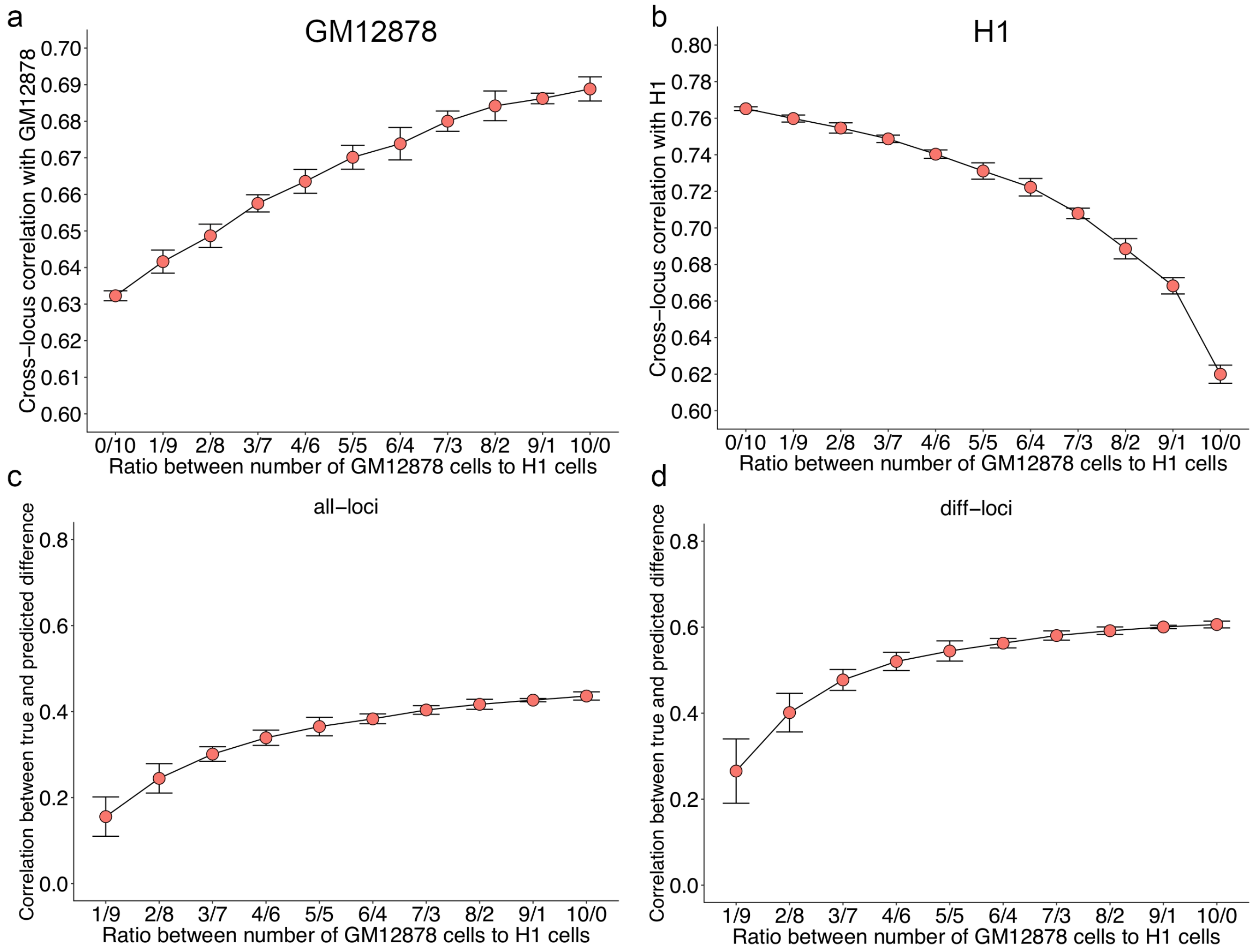

Figure S12. The influence of input sample heterogeneity on predicting chromatin landscape of a pure cell type. (**a**) Pearson's Correlation between the predicted chromatin accessibility and bulk DNase-seq signals in GM12878 for cell mixtures with different proportion of GM12878 (0, 1, 2…, and 10) and H1 (10, 9, 8…, and 0) single cells. (**b**) Pearson's correlation between the predicted chromatin accessibility and bulk DNase-seq signals in H1 for cell mixtures with different proportion of GM12878 and H1 single cells. (**c**) Pearson's correlation between the true bulk GM12878-H1 differential DNase-seq signals and the predicted differential signals (GM12878/H1 cell mixtures – pure H1) across all DHSs. (**d**) Pearson's correlation between the true and the predicted differential signals across differential DHSs. For each cell mixture, cells are randomly sampled from the corresponding cell type and pooled together. The average correlation and standard deviation from 10 independent random samplings of cells are shown.

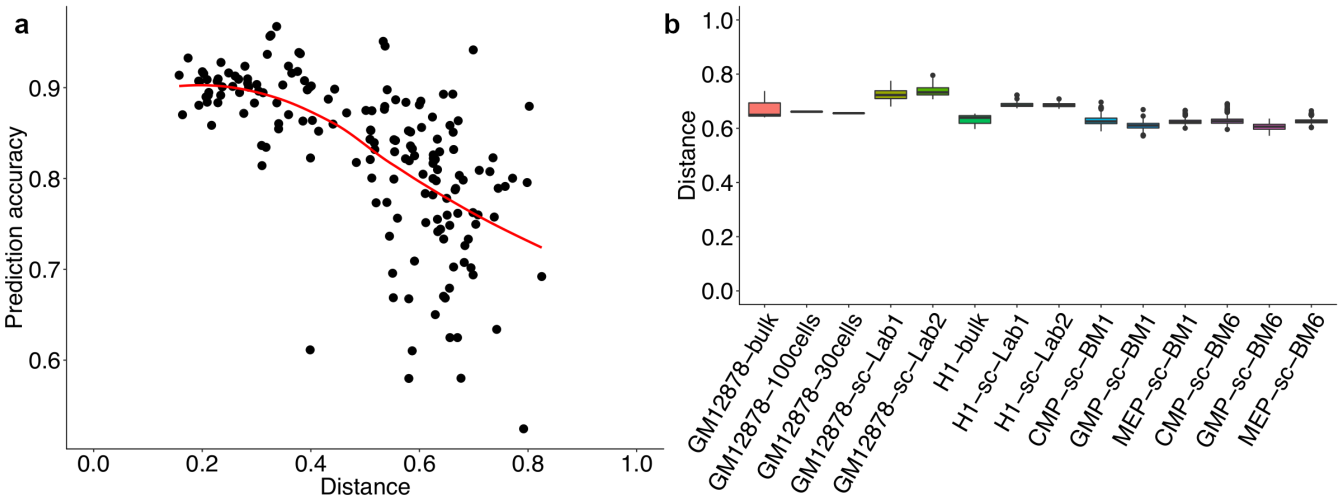

Figure S13. The impact of training-test distance on prediction accuracy. (**a**) Scatterplot showing the relationship between the prediction accuracy (i.e., Pearson's correlation between the predicted DH and true DNase-seq signals across all DHSs) and the training-test distance in a cross-validation analysis. Each dot is a sample. The red curve is the loess fit of the data. (**b**) Training-test distance for the bulk, small-cell-number, and single-cell RNA-seq samples used in this study.
